# An automated Genomes-to-Natural Products platform (GNP) for the discovery of modular natural products

**DOI:** 10.1038/ncomms9421

**Published:** 2015-09-28

**Authors:** Chad W. Johnston, Michael A. Skinnider, Morgan A. Wyatt, Xiang Li, Michael R. M. Ranieri, Lian Yang, David L. Zechel, Bin Ma, Nathan A. Magarvey

**Affiliations:** 1 Department of Biochemistry & Biomedical Sciences, M. G. DeGroote Institute for Infectious Disease Research; McMaster University, Hamilton, Ontario, Canada L8N 3Z5; 2 Department of Chemistry & Chemical Biology, M. G. DeGroote Institute for Infectious Disease Research, McMaster University, Hamilton, Ontario, Canada L8N 3Z5; 3 The David R. Cheriton School of Computer Science, University of Waterloo, Waterloo, Ontario, Canada N2L 3G1; 4 Department of Chemistry; Queens University, Kingston, Ontario, Canada K7L 3N6

## Abstract

Bacterial natural products are a diverse and valuable group of small molecules, and genome sequencing indicates that the vast majority remain undiscovered. The prediction of natural product structures from biosynthetic assembly lines can facilitate their discovery, but highly automated, accurate, and integrated systems are required to mine the broad spectrum of sequenced bacterial genomes. Here we present a genome-guided natural products discovery tool to automatically predict, combinatorialize and identify polyketides and nonribosomal peptides from biosynthetic assembly lines using LC–MS/MS data of crude extracts in a high-throughput manner. We detail the directed identification and isolation of six genetically predicted polyketides and nonribosomal peptides using our Genome-to-Natural Products platform. This highly automated, user-friendly programme provides a means of realizing the potential of genetically encoded natural products.

## Abstract (web summary)

Microbial natural products represent a large reservoir of potential pharmaceutical agents. Here, Johnston *et al*. describe a computer-automated programme for connecting genome sequences with identified and isolated natural products.

## Graphical abstract

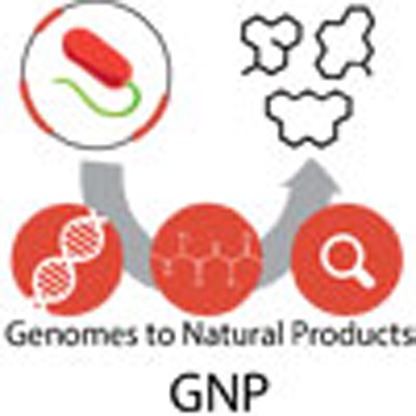

Natural products are valuable small molecules, whose unique and diverse chemical scaffolds have made them an important source of human therapeutics^1^ and industrial agents^2^. Polyketides and nonribosomal peptides are two of the most important^3^ and diverse^4^ classes of these secondary metabolites, and are constructed by assembly line-like enzymes known as polyketide synthases (PKSs) and nonribosomal peptide synthetases (NRPSs)^5^. With the advent of rapid and inexpensive bacterial genome sequencing, a wealth of orphan NRPS and PKS gene clusters have been uncovered in publicly accessible genomes (>25,000 c. 2015) of both well-6 and under-studied^7^,^8^ microbes, prompting renewed enthusiasm for discovery of new natural products^9^. Chemical structures or key monomers of polyketides and nonribosomal peptides can be postulated from genetic information^10^,^11^,^12^,^13^,^14^,^15^, but available computational tools for identifying compounds within complex mass spectral data generally require extensive knowledge and manual annotation of specific organisms^16^,^17^,^18^, metabolites^19^ and mass spectrometry (MS) data^20^,^21^,^22^. Moreover, current tools available to partially automate these processes may require formal training in bio- or chemoinformatics or computer science to achieve results. The development of workflows to connect genomic to metabolomic data has significantly advanced the study of natural products, but now, highly automated and user-friendly software is required to access the wealth of genetically encoded natural products in both new and old microbial producers in a high-throughput context. Here we present the Genomes-to-Natural Products platform (GNP) as an accessible and automated tool that can generate and utilize natural product predictions to directly identify desired small molecules in liquid chromatography–MS/MS (LC–MS/MS) data, to facilitate the re-engagement of microbial libraries for discovering targeted molecules *en masse*, and for uncovering the remaining majority of genetically encoded polyketides and nonribosomal peptides. GNP is available as a web application and can be accessed at http://magarveylab.ca/gnp/.

## Results

### Development of GNP

**Figure 1:**
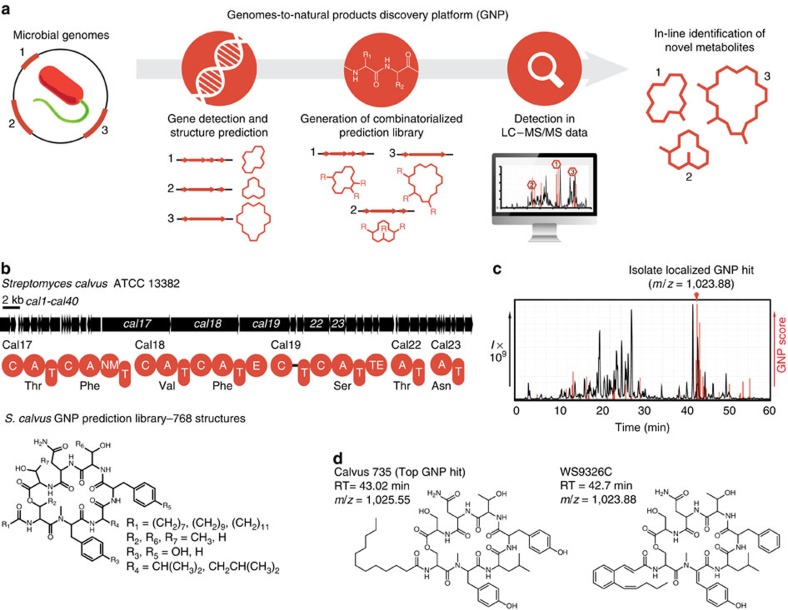
The GNP. (**a**) The automated GNP pipeline processes submitted sequences to identify NRPS and PKS gene clusters and yield predicted structures. These predictions can be combinatorialized and elaborated to account for biosynthetic promiscuity, creating libraries of hypothetical structures that are used to search LC–MS/MS data and automatically reveal the true genetically encoded natural product. (**b**) A novel nonribosomal peptide biosynthetic gene cluster identified within *S. calvus,* alongside the combinatorialized GNP-generated structure prediction. (**c**) LC–MS/MS chromatogram of an *S. calvus* culture extract with GNP result indicating a localized genetically predicted structure. (**d**) Chemical structures of the top-scoring GNP-generated structure prediction (left) and the corresponding natural product, WS9326C (right), depicted with corresponding retention times (RTs) and mass to charge ratios (*m*/*z*).

To expedite the discovery of genetically encoded polyketides and nonribosomal peptides, we developed GNP to automatically predict and locate these metabolites within LC–MS/MS data of crude microbial extracts (Fig. 1a and Supplementary Figs 1–4). GNP predicts chemical structures by identifying gene clusters, modules, domains and domain substrate specificities with a series of hidden Markov models and curated BLAST databases (Supplementary Fig. 1), which are well-suited for defining biosynthetic gene clusters^23^, adenylation domains^13^ and other substrate-specific enzymes. Automatically generated predictions are forwarded to GNP's browser-rendered structure combinatorialization interface^24^,^25^ where scaffolds may be modified to construct libraries of hypothetical products, to account for biosynthetic promiscuity, variation or inaccurate predictions (Supplementary Fig. 2). Combinatorial libraries of predicted chemical scaffolds are loaded alongside our previously published structure library^26^, and are fragmented *in silico* along a series of well-documented fragmentation pathways, including water losses, amide cleavages and ester cleavages. Natural product identification is achieved by matching *in silico* fragments of these known and predicted metabolites to real MS/MS fragments, using validated scoring algorithms^26^ to locate molecules in LC–MS/MS chromatograms (Fig. 1a and Supplementary Figs 3 and 4). This profiling of parent and fragment ions from *in silico* and real LC–MS/MS data allows GNP to identify putative substructures and probability scores to directly locate the products of orphan NRPS and PKS gene clusters. In addition to a browser-rendered spreadsheet, a deconvoluted prediction-guided discovery chart is provided with each GNP report, displaying hits for user-defined predicted structures and confidence scores for predicted structures alongside their retention times in a pseudo-chromatogram (Supplementary Fig. 4). To validate that this automated discovery tool could use genes to find natural products, we investigated orphan NRPS, PKS and hybrid gene clusters from a diverse series of bacterial phyla.

As a test of our automated discovery pipeline, we chose to investigate a novel NRPS gene cluster identified within the genome of *Streptomyces calvus* (American Type Culture Collection (ATCC) No. 13382; Supplementary Table 1). This unusual cluster was found to possess two *trans*-acting adenylation–thiolation didomains, along with an initial acylating condensation domain common to lipopeptides^27^ (Fig. 1b and Supplementary Fig. 5a). GNP generated a predicted product scaffold that could be combinatorialized to automatically generate a library of 768 hypothetical molecules (Supplementary Fig. 5b). This predicted structure library was fragmented *in silico* and used to survey for potential matches in LC–MS/MS data of a *S. calvus* culture extract. GNP identified a series of metabolites eluting after 43 min in the *S. calvus* LC–MS chromatogram, corresponding to the predicted molecule calvus735 (Fig. 1c and Supplementary Figs 5c and 6). Isolation of the indicated metabolites led to the identification of the nonribosomal peptides WS9326A and WS9326C (ref. 28), products of this hitherto undescribed NRPS gene cluster (Fig. 1d and Supplementary Tables 2–3). Conveniently, because GNP uses MS/MS-fragment matching to determine hits, it was capable of assigning large portions of the WS9326 before structure determination by NMR spectroscopy.

### New nonribosomal peptides from *Acidovorax* and *Variovorax*

**Figure 2:**
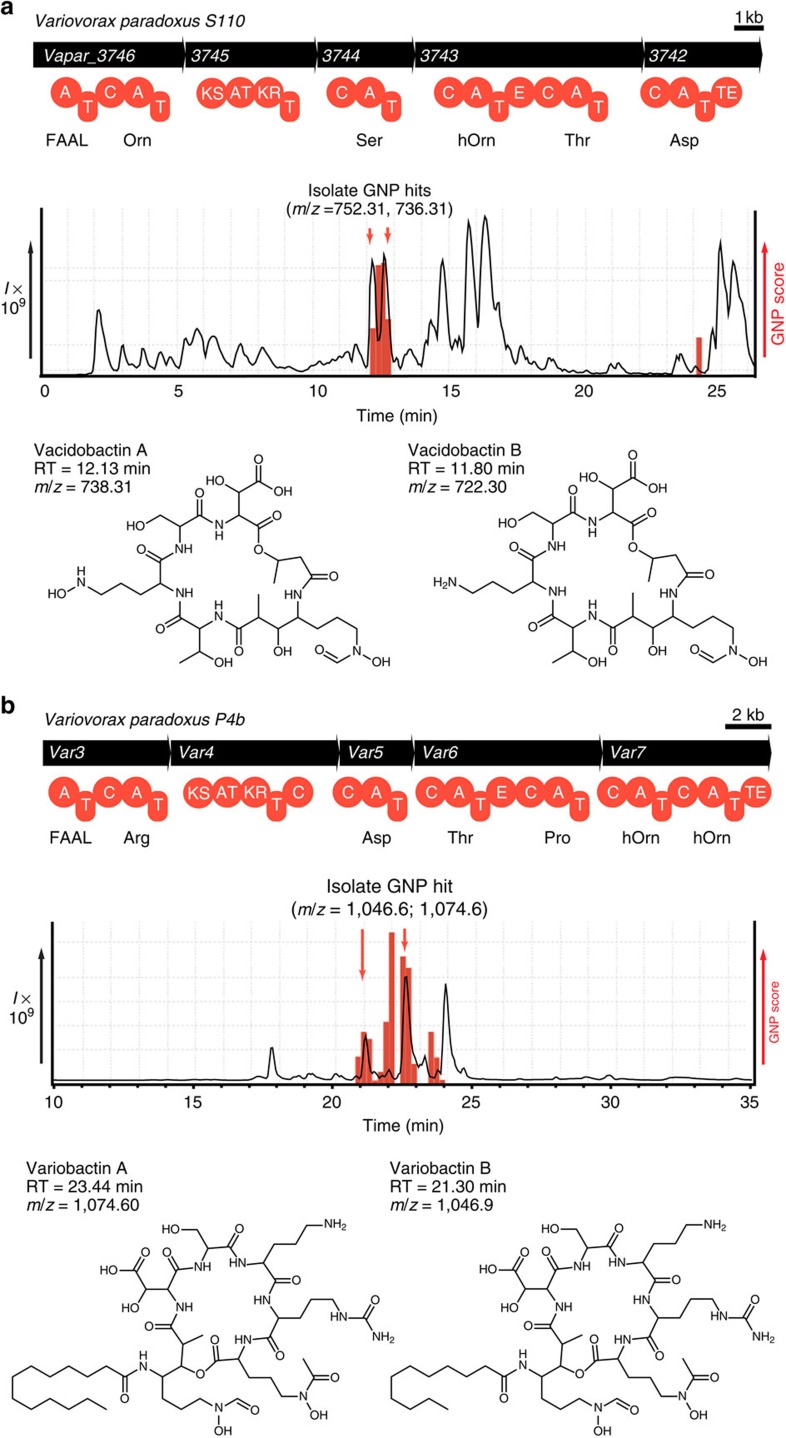
Identifying novel hybrid nonribosomal depsipeptides from unexplored *Variovorax* strains. (**a**) GNP analysis of a novel NRPS–PKS gene cluster in *V. paradoxus* S110 provided a prediction that was combinatorialized and used to query corresponding LC–MS/MS data with GNP, revealing the true genetically encoded natural products. Isolation of the indicated compounds yielded novel metabolites vacidobactin A and B, depicted with corresponding retention times (RTs) and mass to charge ratios (*m*/*z*). (**b**) GNP analysis of a novel NRPS–PKS gene cluster in *V. paradoxus* P4B provided a structure prediction that was combinatorialized and used to query LC–MS/MS data with GNP. Isolation of the indicated compounds yielded novel metabolites variobactin A and B, depicted with corresponding RT and mass to charge ratios (*m*/*z*).

While *Streptomyces* are well-known producers of natural products, extensive microbial genome sequencing has revealed NRPS and PKS machinery in exotic, untouched branches of the microbial tree of life^29^. In light of recent genome-guided discoveries^7^,^8^,^30^, we chose to investigate a series of previously unstudied Proteobacteria. A novel NRPS–PKS cluster was discovered in the genome of *Acidovorax citrulli* (Leibnitz Institute DSMZ-German Collection of Microorganisms and Cell Cultures, DSM no. 17060), with GNP automatically generating a predicted scaffold molecule (Supplementary Fig. 7a and Supplementary Table 4). This prediction was combinatorialized for potential macrocyclization or amino-acid substitutions, and to account for neighbouring *N*-acetyl and *N*-formyltransferases (Supplementary Fig. 7b). The resulting library of 576 potential structures was used to automatically reveal targeted metabolites present in LC–MS/MS data, detecting a series of hits eluting after 12.1 min (Supplementary Fig. 7c). Isolation of the most substantial hits yielded novel polyketide/nonribosomal peptide natural products—the first from this genus—acidobactins A and B, whose final structures were solved by NMR (Supplementary Figs 7d, 8–11 and Supplementary Table 5). The only significant point of variation between the predicted and final structures stems from the unusual initiating adenylation domain, acting as a fatty acyl-AMP ligase (FAAL)^31^, for which no specificity codes have yet been revealed. To correct this, we constructed an extensive FAAL database of domains from established assembly lines to enable substrate prediction. As a test of this improved predictive capacity we investigated an acidobactin-like gene cluster in the related and similarly uninvestigated organism *Variovorax paradoxus* S110 (DSM No. 30034; Fig. 2a and Supplementary Table 6). Automated scaffold generation returned a prediction that was identical to acidobactin but with an enabled FAAL substrate prediction (Supplementary Fig. 12a). Following combinatorialization that was identical to that of the acidobactin scaffold (Supplementary Fig. 12b), GNP located two peaks from the *V. paradoxus* S110 LC–MS/MS sample (Fig. 2a). Isolation of these indicated metabolites provided the novel compounds vacidobactin A and B, which are methylmalonate-incorporating acidobactins and represent the first natural products isolated from *Variovorax* (Fig. 2a, Supplementary Figs 12c, 13–16 and Supplementary Table 5). A second *Variovorax* isolate (*V. paradoxus* P4B (ref. 32)) was found to possess another distinct NRPS/PKS gene cluster, corresponding to a putative lipopeptide (Fig. 2b, Supplementary Fig. 17a and Supplementary Table 7). By altering amino-acid composition, macrocyclization status and tailoring modifications, we generated a library of 32 structures (Supplementary Fig. 17b) for use in GNP analysis of the LC–MS/MS spectrum. The crude extract of *V. paradoxus* P4B was shown to contain a series of candidate hits (Fig. 2b) whose isolation yielded the novel natural products variobactin A and B, in spite of several unforeseen amino-acid substitutions (Fig. 2b and Supplementary Figs 17c, 18–20), as confirmed by MS/MS annotation (Supplementary Fig. 21) and NMR spectroscopy (Supplementary Fig. 18, Supplementary Table 8 and Supplementary Note 1).

### Discovery of glycosylated natural products and polyketides

In addition to the prediction of nonribosomal peptides, GNP is capable of predicting polyketide structures, as demonstrated by the prediction of ketide units within the acidobactins, vacidobactins and variobactins. Although modular (type 1) polyketides are more readily predictable than other classes of microbial natural products^4^,^5^ such as terpenes, their MS/MS fragmentation patterns are less information rich than those of peptidic natural products. However, polyketide biosynthetic gene clusters frequently possess enzymatic machinery for the biosynthesis of highly modified deoxysugar moieties^22^,^33^, whose characteristic masses can facilitate the identification of polyketides in a tandem mass spectrum^34^. To automate the prediction of deoxysugars from genomic information, we constructed a library of hidden Markov models corresponding to important families of deoxysugar biosynthesis genes^22^,^35^ (Supplementary Table 9). We revised and expanded the glycogenomic code developed by Kersten *et al*.^22^, in particular by extending their logic to the biosynthesis of pentose deoxysugars^36^ (Supplementary Table 10; see Methods). Novel logic was required to predict deoxysugars from sequence data, as polyketides often contain multiple sugars^37^, enzymes are shared between biosynthetic pathways^38^, and these pathways may not be physically segregated within a given cluster^38^. We therefore developed an algorithm to predict sugar combinations. The number of deoxysugar glycosyltransferases is determined based on a homology search (Supplementary Fig. 22), and all possible sugar combinations of that size are iteratively evaluated based on an analysis of whether the identified deoxysugar pathway genes are both necessary and sufficient for the biosynthesis of a given combination (see Methods). Subsequent to user-directed combinatorialization, each scaffold in the combinatorial library is then glycosylated with each sugar combination at a random hydroxyl group to produce a glycosylated scaffold library. Glycosidic bond cleavage can then be enabled based on the identification of oxan-2-ol substructures.

**Figure 3:**
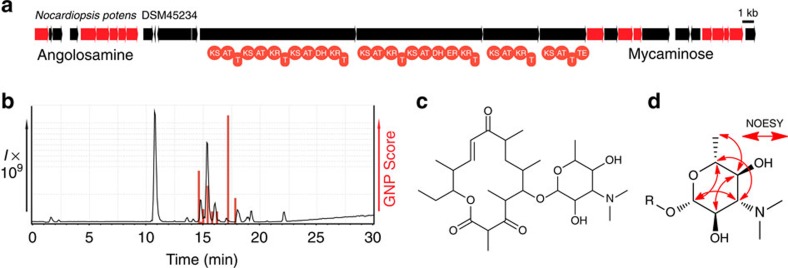
Automated prediction, detection and structure elucidation of a glycosylated polyketide from *N. potens*. (**a**) A biosynthetic gene cluster containing machinery for deoxysugar and polyketide biosynthesis was identified in the genome of *N. potens* (DSM 45234). The GNP-generated polyketide and sugar predictions were combinatorialized to yield a library of 42 hypothetical products. (**b**) GNP analysis of LC–MS/MS data from a *N. potens* extract revealed four related candidate peaks. The most abundant of the detected hits—predicted polyketide 10 (**c**)—was isolated for structure elucidation by NMR. (**c**) Structure of the isolated glycosylated polyketide potensimicin. (**d**) NOESY NMR spectroscopy of the potensimicin deoxysugar demonstrates it is mycaminose.

To test these new models and assess whether GNP could also be used both to predict and detect glycosylated polyketides from crude extracts, we decided to search for the product of a unique deoxysugar and PKS gene cluster found in the genome of *Nocardiopsis potens* (DSM 45234; Fig. 3a, Supplementary Fig. 23a and Supplementary Table 11). Processing this biosynthetic gene cluster with GNP yielded a polyketide backbone, which could be tailored with two predicted deoxysugars, mycaminose (or its diastereomer ravidosamine) and angolosamine. By combinatorializing cyclization, sugar appendages, and methylmalonate or malonate incorporation, we developed a library of 42 hypothetical structures (Supplementary Fig. 23b). To assess whether our hypothetical polyketides could be present in a *N. potens* extract LC–MS/MS data file we enabled fragmentation of potential macrocycle esters, losses of ketoreductase generated hydroxyls, as well as glycosidic cleavages. GNP revealed four related peaks within our extract, including a consensus match for the most abundant hit, predicted polyketide 10 (Fig. 3b,c and Supplementary Figs 23c, 24–26). Isolation and NMR-based structure elucidation of this putative polyketide revealed a planar structure identical to the matched prediction, including a narbonolide-type macrocyclic polyketide scaffold^39^ and an *O*-linked mycaminose or ravidosamine deoxysugar (Fig. 3c, Supplementary Tables 12–13). However, while LC–MS/MS-based techniques can provide useful information to assist in structure elucidation, they cannot readily provide insight into stereochemsitry. Analysis of the potensimicin NOESY NMR spectrum and anomeric carbon coupling constant (*J*_*hz*_=7.06) (ref. 40) demonstrated that the potensimicin deoxysugar was β-mycaminose (Fig. 3d). Potensimicin appears similar to mycaminose-modified narbonolide compounds that have been observed previously^41^,^42^, and shares similar bioactivity profiles (Supplementary Table 14).

### Identification and structure elucidation of thanamycin

**Figure 4:**
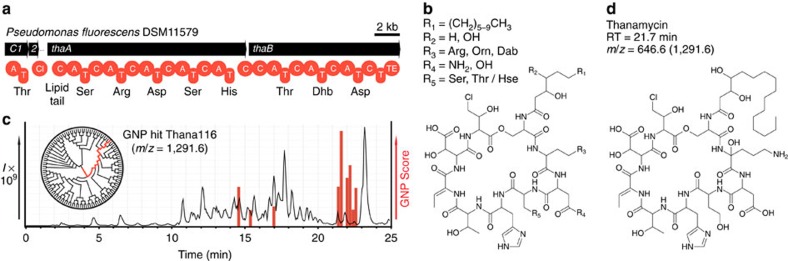
GNP-facilitated detection and structure elucidation of the cryptic nonribosomal peptide thanamycin. (**a**) Biosynthetic gene cluster for thanamycin identified within *P. fluorescens* DSM11579. (**b**) Combinatorialization of the GNP-generated structure prediction. (**c**) GNP prediction-guided discovery chart indicates a series of related thanamycin-like ions from a *P. fluorescens* extract, including the main ion (1,291.6 *m*/*z*), which was found to possess the most structural similarity to predicted structure Thana116 from 120 hypothetical variants, shown as a chemoinformatic tree clustered by chemical similarity. (**d**) Structure of thanamycin with corresponding retention time (RT) and observed mass to charge ratio (*m*/*z*).

Biosynthetic gene clusters are being uncovered at an increasing rate, presenting new opportunities for the discovery of clinically relevant polyketides and nonribosomal peptides. However, low abundance or cryptic metabolites have proven to be a significant bottleneck in genome-guided efforts^43^,^44^,^45^, as they are challenging to detect, and require inordinate processing to yield sufficient quantities of pure material for structure elucidation with NMR^45^. GNP has demonstrated itself to be a quick and sensitive means of detecting natural products, with routine natural product identification occurring with only nanograms of material reaching our mass spectrometer (Supplementary Table 15). In addition, MS filters and fragment matching allow GNP to narrow in on predicted molecules that are highly similar to the final product, providing assistance for downstream structure elucidation efforts. To demonstrate the utility of this approach, we used GNP to investigate the cryptic lipopeptide thanamycin^43^, whose production, isolation and elucidation have proven elusive despite a series of genetic^43^ and MS-based studies^18^. We identified a thanamycin biosynthetic gene cluster in the recently sequenced genome of *Pseudomonas fluorescens*^46^ (DSM No. 11579; Fig. 4a, Supplementary Fig. 27a and Supplementary Table 16). To reveal the physical location of this cryptic metabolite in chromatograms, we used GNP to generate a structure library (Fig. 4b and Supplementary Fig. 27b) that would identify the most similar chemical structure from a complex extract. GNP utilized hypothetical MS-fragment matching to indicate one candidate structure from the library of 120 predictions, revealing a low-abundance molecule, with *m/z*=1,291 [M+H]^+^ (Fig. 4c and Supplementary Fig. 27c). We first sought to verify our GNP match by manual tandem-MS annotation (Supplementary Fig. 28), confirming the partial amino-acid sequence proposed previously^18^, as well as an additional threonine or homoserine residue, as had been predicted by GNP. Next, ^13^C-amino-acid incorporation was used to confirm the presence of the predicted ornithine- (Supplementary Fig. 29) and threonine-derived residues (Supplementary Fig. 30), while providing further evidence for the presence of homoserine. As a conclusive means of assigning its structure, we undertook substantial efforts to obtain sufficient material for NMR experiments. Thanamycin had been initially identified at levels of <0.1 mg l^−1^, which was increased to 0.33 mg l^−1^ through culture and purification optimization, reaching a final yield of 40 mg from 120 l of low-volume cultures, sufficient for accessing the structure of thanamycin by NMR spectroscopy (Fig. 4d, Supplementary Figs 31–32 and Supplementary Table 17). Our observations demonstrated that GNP had incorrectly assigned the third amino acid as asparagine instead of the observed and originally predicted aspartic acid, though it did correctly detect the presence of ornithine as the second amino acid. Surprisingly, the source of the mass discrepancy between the predicted and observed thanamycin structures appears to be an exceptionally rare hydroxylation at the ornithine α-carbon, a modification previously observed in various diketopiperazines^47^,^48^ and the lipodepsipeptide skyllamycin^49^. This unusual modification may be associated with the potent antifungal activity of thanamycin, which is 32 times that of the related natural product syringomycin E. (Supplementary Table 14). This final example illustrates the manner in which GNP provides a rapid means of predicting, locating and partially solving the structures of assembly line-derived natural products, such as modular polyketides, nonribosomal peptides and glycosylated natural products.

## Discussion

The evolved bioactivities and unique chemical structures of microbial natural products have made them a valuable source of pharmaceutical and industrial agents^1^,^2^,^50^. The revelation that even well-studied producers, such as *Streptomyces coelicolor*^6^,^51^, possess gene clusters for many more natural products than they are known to produce has sparked renewed interest in natural product discovery^52^. A number of novel methods have therefore been developed in recent years in an attempt to bridge the gap between genomic potential and natural product discovery. Initial work to reveal and sequence complex nonribosomal peptides in mass spectral data built on advances in *de novo* sequencing techniques of proteomics, allowing researchers to reveal structural information about cyclic peptides^53^. Simultaneously, the development of computational approaches for predicting nonribosomal peptide adenylation domain specificities facilitated accurate prediction of NRPS small-molecule products^10^. By bridging these advances in genomics-based structure prediction and MS-driven sequence annotation, Kersten *et al*. defined a manual peptidogenomic workflow to identify peptidic natural products—both ribosomal and nonribosomal—based on manually generated structure predictions and manually annotated mass tags, marking gaps between MS^2^ fragment ions that could be annotated as amino acids^21^. A similar workflow was developed for the discovery of glycosylated natural products, combining manually annotated mass tags with the manual analysis of biosynthetic gene clusters^22^. Recently, various degrees of computer automation have been applied to the peptidogenomics workflow. In Pep2Path^20^, a series of manually annotated mass tags from a chosen peak of interest can be queried against genomic data processed by antiSMASH 2.0 (ref. 54) and NRPSPredictor2 (ref. 10) using a Bayesian algorithm, to associate user-annotated amino-acid sequences with NRPS gene clusters. Recent advances such as RiPPQuest^55^ and NRPQuest^56^, developed by Mohimani *et al*., represent efforts to integrate lanthipeptide and nonribosomal peptide prediction and detection, based on genomic and MS/MS data, within a single software package. Molecular networking is a spectral alignment tool capable of clustering MS scans with closely related fragmentation patterns^18^,^57^, although determining the identity of clustered ions and the validity of their association requires a high degree of manual interrogation of results^16^,^17^,^18^,^19^, including extensive annotation with known molecules^19^, gene clusters^16^,^17^ or genetic knockouts^18^ to identify ions of interest, typically through their extensive similarity to known compounds. For a summary and comparison of recent genomic- and metabolomic-based natural products discovery methods, see Supplementary Fig. 33.

GNP's structure-prediction engine expands the chemical search space relative to previously published methodologies (Supplementary Fig. 33) by providing an integrated platform for the prediction of nonribosomal peptide, type I polyketide and deoxysugar-containing natural products. Importantly, GNP does not rely on the manual annotation of a mass spectrum^16^,^17^,^18^ or candidate peak^20^, and thereby significantly increases throughput. While the aforementioned methodologies have represented important advances in linking genomic to metabolomic data in the context of natural product discovery, many are distributed as binaries^20^ or with minimal user interfaces^55^,^56^ that may render them inaccessible to many users without formal bioinformatics or computer science training. In contrast, GNP offers a continuous workflow for genomic and metabolomic discovery, integrated into a single web application with a user-friendly interface accessible to chemists or microbiologists without chemo- or bioinformatics training. Further, GNP provides access to data and matched fragments used to generate candidate scores and probable structures for detected natural products, available as easily interpreted, information-rich and toggle-able report tables. Our library of hidden Markov models and BLAST databases represent an advance over support vector machine-based prediction methods^13^, enabling the generation of accurate predictions across bacterial phyla and thereby facilitating the discovery of novel compounds from bacteria that have not previously been known to produce natural products (Fig. 2). As GNP reproduces common MS/MS fragmentation pathways such as amide-, ester- and glycosidic cleavages *in silico* to generate appropriate fragments to match and detect candidate hits, the scope of natural products to which it can be applied is primarily limited by the availability of methods for reliable automated structure prediction. A recent manual workflow for hypothetical structure enumeration and fragment matching described by Zhang *et al*.^58^ also appears to show promise in predicting correct structures of purified ribosomal peptide natural products using custom-tailored high-resolution mass spectral techniques, and suggests the automated combinatorialization and identification programme presented in GNP may be also applicable to these diverse molecules given an appropriate automated structure prediction methodology.

Although LC–MS-based platforms have seen extensive development and use in the past decade, they are susceptible to limitations of MS-centric chemical detection. First, specific chemical configurations cannot be absolutely determined by MS alone. LC–MS/MS-based approaches can provide evidence for a given structure, but NMR remains necessary to reveal bond order and stereochemistry. Second, the sensitivity of MS-based detection is based both on the abundance and ease of ionization of a given molecule, such that not all molecular species will be detected equally. Third, MS^2^ rarely provides a completely comprehensive sampling of plausible fragmentation pathways. Although GNP is capable of generating and matching a diverse series of MS/MS fragments, only a small fraction of these are typically observed and complete coverage of a given molecule is rare, particularly for larger structures. Unlike approaches that utilize manually annotated mass tags^20^,^21^,^22^, GNP does not require user intervention to identify MS/MS fragmentation pathways, and is capable of sampling all potential pathways simultaneously, providing results rapidly and facilitating a high-throughput workflow. Despite the limitations of LC–MS/MS, it remains the primary method for the rapid interrogation of complex mixtures of bacterial metabolites. While NMR spectroscopy will remain a necessity for structure elucidation for the foreseeable future, informatic MS/MS-based platforms such as GNP represent a powerful means of linking metabolomic and genomic data for the discovery of novel assembly line-derived natural products.

In this work, we present a GNP to predict and identify assembly line-derived natural products from a growing and diverse array of sequenced bacterial genomes. By providing an accessible and user-friendly interface, and automating the detection of natural products from biosynthetic assembly lines, GNP represents a high-throughput tool for the discovery of uncovering novel small molecules without the need for genetic knockouts or engineering, bioactivity testing or extensive manual MS annotation. This unique approach highlights the advantages of automated and integrated informatic discovery tools by leveraging advances in genomics and metabolomics to access novel natural products.

## Methods

### General experimental procedures

One-dimensional (^1^H and ^13^C) and two-dimensional (^1^H–^13^C heteronuclear multiple-bond correlation spectroscopy (HMBC), heteronuclear single quantum coherence spectroscopy (HSQC), and ^1^H–^1^H nuclear Overhauser effect spectroscopy (NOESY) and correlation spectroscopy (COSY)) NMR spectra for acidobactins, vacidobactins and variobactin were recorded on a Bruker AVIII 700 MHz NMR spectrometer in D_2_O (D_2_O; Cambridge Isotope Laboratories). NMR spectra for thanamycin and potensimicin were recorded on a Bruker AVIII 700 MHz NMR spectrometer in dimethylsulfoxide (DMSO-d_6_; Cambridge Isotope Laboratories). NMR spectra for potensimicin were also recorded in chloroform (CDCl_3_; Cambridge Isotope Laboratories). NMR spectra for WS9326A were recorded on Bruker Avance 500 and 600 MHz instruments in dimethylsulfoxide (DMSO)-d_6_. High-resolution MS spectra were collected on a Thermo LTQ OrbiTrap XL mass spectrometer (ThermoFisher Scientific, USA) with an electrospray ionization source and using collision-induced dissociation (CID) with helium for fragmentation. LC–MS data were collected using a Bruker AmazonX ion trap mass spectrometer coupled with a Dionex UltiMate 3000 HPLC system, using a Luna C18 column (150 or 250 × 4.6 mm, Phenomenex) for analytical separations, running acetonitrile with 0.1% formic acid and ddH_2_O with 0.1% formic acid as the mobile phase. MS^*n*^ measurements were made by direct infusion to a Bruker AmazonX mass spectrometer. All data required for GNP identification of the compounds identified in this study, including.FASTA files, formatted combinatorial structure libraries and.mzXML files, are available for download from http://magarveylab.ca/data/gnp/.

### Microbial strains

*A. citrulli* AAC00-1, *V. paradoxus* S110 and *N. potens* were ordered from the German Resource Centre for Biological Material (DSMZ, DSM No. 17060, 30034 and 45234) and cultured on Acidovorax Complex Media^30^ (*A. citrulli*, *V. paradoxus*; acidovorax complete media) or Bennett's media (*N. potens*). The environmental isolate *V. paradoxus* strain P4B was found in a soil sample collected from McMaster University in 2010 and maintained on tryptic soy broth (TSB) media. *S. calvus* was obtained from the ATCC (No. 13382) and was cultured on mannitol soy agar. *P. fluorescens* was obtained from DSMZ (DSM No. 11,579) and was regularly maintained on Luria–Bertani agar. All bacteria were cultured at 30 °C.

### Fermentation and small-molecule isolation

*WS9326A*. The production medium consisted of 10 g l^−1^ dextrin, 10 g l^−1^ tryptone, 2 g l^−1^ NaCl, 2 g l^−1^ (NH_4_)_2_HPO_4_, 1.5 g l^−1^ KH_2_PO_4_, 0.5 g l^−1^ K_2_HPO_4_ and 0.25 g l^−1^ MgSO_4_ · 7H_2_O. The pH of the medium was adjusted to 7.2 before autoclaving. After autoclaving 5 ml l^−1^ of the following trace element solution (sterile filtered) was added: 2 g l^−1^ MgSO_4_, 2 g l^−1^ ZnSO_4_·7H_2_O, 2 g l^−1^ FeSO_4_ ·7H_2_O, 2 g l^−1^ MnCl_2_ ·4H_2_O, 2 g l^−1^ CaCl_2_ ·2H_2_O, 2 g l^−1^ NaCl, 0.4 g l^−1^ CuCl_2_ ·2H_2_O, 0.4 g l^−1^ B(OH)_3_, 0.2 g l^−1^ Na_2_MoO_4_, 0.2 g l^−1^ CoCl_2_ and 2.2 g l^−1^ sodium citrate. Single colonies of *S. calvus* grown on mannitol soy agar were used to initiate 25 ml cultures of TSB and grown to high cell density over 3 to 4 days at 28 °C and 180 r.p.m. The starter culture was used to inoculate 4 × 500 ml production medium (1% v/v) in 2 l baffled shake flasks containing steel springs, which were then incubated for 4 days at 180 r.p.m. and 28 °C. The cells were removed by centrifugation and the culture supernatant (1.8 l) was mixed with Diaion HP-20 resin (10 g l^−1^) for 3 h. The resin was collected by vacuum filtration and washed with water (2 × 300 ml) then acetone (3 × 200 ml). The acetone washes were concentrated *in vacuo* and the resulting aqueous residue extracted three times with an equivalent volume of ethyl acetate. The organic fractions were dried over anhydrous Na_2_SO_4_ then concentrated to dryness *in vacuo*. The crude residue was dissolved in methanol (1 ml) then injected onto a Biotage system equipped with a Biotage Flash 25+M reverse phase column (KP-C18-HS, 25 × 150 mm, 48 ml column volume). WS9326A was resolved by eluting the column with 10% acetonitrile/water (0.5% acetic acid) for 150 mL, followed by a gradient of 10% acetonitrile/water (0.5% acetic acid) to 90% acetonitrile/water (0.5% acetic acid) over 350 ml, then holding the final solvent composition for 150 ml, all at a flow rate of 10 ml min^−1^. Fractions containing WS9326A were pooled and concentrated to dryness, yielding 5 mg of pure compound.

*Acidobactins, vacidobactins and variobactin*. Fresh colonies of *A. citrulli* AAC00-1 or *V. paradoxus* S110 were inoculated into 2.8-l glass Fernbach flasks containing 1 l of acidovorax complete media^30^. Environmental strain *V. paradoxus* P4B colonies were used to inoculate 2.8-l glass Fernbach flasks containing 1 l water, 10 g casitone, 1 g MgSO_4_ ·7H_2_O, 1 g CaCl_2_ ·2H_2_O, 50 mM HEPES buffer and 20 g l^−1^ Diaion HP-20 resin with pH adjusted to 7.0 (ref. 20). Cultures were grown at 30 °C, shaking at 190 r.p.m. for 3 days, after which *A. citrulli* AAC00-1 and *V. paradoxus* S110 cells were pelleted by centrifugation at 7,000 r.p.m. for 15 min, adding HP-20 resin to the supernatant at 20 g l^−1^, and subsequently shaking for 2 h at 220 r.p.m. Resins were collected by Büchner funnel filtration and washed with 400 ml of distilled water before eluting three times with 400 ml of methanol. The methanol eluents were evaporated to dryness under rotary vacuum. Acidobactins and vacidobactins were purified using a Luna 5 μm C_18_ column (Phenomenex, 250 × 10 mm). The mobile phase was 2% acetonitrile with 0.1% formic acid, and 98% water with 0.1% formic acid at 2 min, increasing to 9% acetonitrile at 23 min at a flow rate of 6 ml min^−1^. Acidobactin A eluted at 15.5 min, acidobactin B eluted at 15.9 min, vacidobactin A eluted at 15.7 min and vacidobactin B eluted at 16.2 min. Variobactin was purified using a Luna 5 μm C_18_ column (Phenomenex, 250 × 15 mm). The mobile phase was 5% acetonitrile with 0.1% formic acid, and 95% water with 0.1% formic acid at 0 min with a flow rate of 2.5 ml min^−1^ increasing to 8 ml min^−1^ at 1.5 min for an additional 3.5 min. The gradient increased in a linear fashion from 5 to 10 min to 10% acetonitrile then from 10 to 52 min the gradient was linear to 50% acetonitrile. Variobactin A eluted at 38 min.

*Potensimicin*. For production of potensimicin, *N. potens* was first grown in 50 ml of a media containing: 10 g l^−1^ molasses, 10 g l^−1^ meat extract, 10 g l^−1^ peptone and 10 g l^−1^ soluble starch for 2 days at 250 r.p.m. and 28 °C. Following this, 10 ml of starter culture was used to inoculate 1 l of the same media. Cultures were grown for 3 days at 250 r.p.m. and 28 °C before being collected by centrifugation at 7,000 r.p.m., followed by methanol extraction of the cell pellet, and Diaion HP-20 (20 g l^−1^) extraction of the supernatant. Methanol eluent of the HP-20 resin was pooled with the methanol extract of the cell pellet, dried under rotary vacuum and re-dissolved in methanol. The extract was separated on an open gravity column of LH-20 size-exclusion resin (Sephadex) with methanol as a mobile phase. Fractions containing potensimicin were pooled, evaporated to dryness and resuspended in methanol. Potensimicin was isolated by preparative scale LC–MS using a Luna 5 μm C_18_ column (Phenomenex, 250 × 10 mm) with water (0.1% formic acid) and acetonitrile (0.1% formic acid) as the mobile phase, at a flow rate of 8 ml min^−1^. After 3 min, acetonitrile was increased in a linear manner (curve 5) from 5 to 65% at 22 min, followed by a wash of 100% acetonitrile. Potensimicin eluted at 15 min and was isolated at a yield of ∼9 mg l^−1^.

*Thanamycin*. For production of thanamycin, *P. fluorescens* was grown in 400 ml of YM media (3 g l^−1^ malt extract, 3 g l^−1^ yeast extract, 5 g l^−1^ peptone and 10 g l^−1^ glucose) per 2.8-l Fernbach flask for 72 h at 300 r.p.m. and 28 °C until 120 l of culture was accumulated. Cultures were collected by centrifugation at 7,000 r.p.m., followed by methanol extraction of the cell pellet and Diaion HP-20 (20 g l^−1^) extraction of the supernatant. Methanol eluent of the HP-20 resin was pooled with the methanol extract of the cell pellet and dried under rotary vacuum. This extract was dissolved and extracted with a 1:1 mixture of butanol and water. The butanol fraction was isolated, evaporated to dryness, resuspended in a minimal volume of methanol and left to precipitate for 48 h at 4 °C. The precipitant was collected by centrifugation at 4,000 r.p.m. and 4 °C, washed once with methanol and then dissolved in excess DMSO. Thanamycin was isolated using preparative scale LC–MS using a Luna 5 μm C_18_ column (Phenomenex, 250 × 15 mm) with water (0.1% trifluoroacetic acid) and acetonitrile (0.1% trifluoroacetic acid) as the mobile phase, at a flow rate of 10 ml min^−1^. After 2 min, acetonitrile was gradually (curve 7) increased from 5 to 42% at 28 min, then ramping (curve 5) to 51% at 40 min, followed by a wash with 100% acetonitrile. Thanamycin eluted at 35 min.

### Structure elucidation

For methods, tables, figures and spectra pertaining to the NMR and HRMS structure elucidation of isolated natural products, see Supplementary Note 1.

### Incorporation of ^13^C_4_-threonine and ^13^C_5_-ornithine

A fresh colony of *P. fluorescens* was used to inoculate 40 ml of YM media containing 2 mM ^13^C_4_-L-threonine or ^13^C_5_-L-ornithine (Cambridge Isotope Laboratories), which had been added through sterile syringe filtering after autoclaving. Cultures were grown for 48 h before being collected through centrifugation and Diaion HP-20 resin extraction of the supernatant. The SMILES structure of the top GNP thanamycin hit from the first round of GNP was modified with heavy atoms to query threonine incorporation at position 4 (two hypothetical structures) and ornithine incorporation at position 2 (two hypothetical structures). GNP settings identical to those for initial thanamycin detection were used to analyse stable ^13^C incorporation experiment LC–MS/MS data.

### Determination of antimicrobial activity

Minimum inhibitory concentrations for potensimicin and thanamycin were determined using broth microdilution. For potensimicin, activity was determined in cation-adjusted Mueller Hinton broth using *Bacillus subtilis* 168 and *Staphylococcus aureus* Newman as indicator organisms, grown at 28 °C and 37 °C, respectively. Erythromycin was used as an internal control. For thanamycin, activity was determined in YPD media using *Saccharomyces cerevisiae* as an indicator organism, growing at 28 °C, and using syringomycin E as an internal control. The minimum inhibitory concentration (MIC) was determined as the lowest concentration of drug at which no growth was observed after 16 h.

### Genome sequencing

A single colony of *V. paradoxus* P4B was grown in 3 ml TSB overnight at 30 °C and 250 r.p.m. Genomic DNA was collected using a GenElute Bacterial Genomic DNA Kit (Sigma). A single colony of *S. calvus* was used to inoculate a 50-ml culture of GYM media containing 0.5% glycine and grown for 96 h at 30 °C and 250 r.p.m. A volume of 500 μl of culture was centrifuged at 12,000*g* for 5 min and resuspended in 500 μl SET buffer (75 mM NaCl, 25 mM EDTA (pH 8.0), 20 mM Tris HCl (pH 7.5) and 2 mg ml^−1^ lysozyme) to lyse for 2 h at 37 °C. Proteinase K and SDS were added after lysis to final concentrations of 0.5 mg ml^−1^ and 1%, respectively. The lysis mixture was incubated at 55 °C for 2 h before adjusting the concentration of NaCl to 1.25 M and extracting twice with phenol-chloroform. Isopropanol was added (equivalent to 60% the volume of the solution) to precipitate genomic DNA, which was subsequently washed twice with 70% ethanol and resuspended in sterile water for sequencing. Genomic DNA was sent for library preparation and Illumina sequencing at the Farncombe Metagenomics Facility at McMaster University, using an Illumina HiSeq DNA sequencer. Contigs were assembled using the ABySS genome assembly programme^59^ and with Geneious bioinformatic software.

### Bioinformatic methodology and construction of GNP

*File input and options*. GNP has three input modes: genome search, scaffold library generation and LC–MS/MS database-dependent search. Respectively, these modes allow the user to search a genome for natural product biosynthesis clusters, generate a combinatorialized library of predicted scaffold molecules and identify compounds within a LC–MS/MS chromatogram. These three steps are intended to be performed in series to allow the user to isolate natural products based on genomic information.

GNP's genome scan mode searches a sequence file for natural product biosynthesis clusters, and uses its analysis of these clusters to predict nonribosomal peptide and polyketide molecules. The genome search mode can accept whole genomes, condensed DNA sequences (representing either individual clusters or sequence contigs) or translated protein sequences. All sequences must be submitted in FASTA format. In addition to allowing the user to submit a sequence and choose an operating mode, the genome search's web interface allows the user to adjust the maximum length between genes to consider them part of the same biosynthetic cluster, and to adjust the cutoffs for what is considered a statistically significant score for four classes of domains: adenylation, acyltransferase, thiolation or thioesterase, and all other results.

*Genome search*. In whole genome searches, Glimmer 3 (ref. 60) is used to locate putative biosynthetic genes. Glimmer first identifies long, non-overlapping open reading frames within the genome, then uses these sequences to construct an interpolated context model of coding sequences. This model is then used to analyse the genome and predict biosynthetic sequences. For shorter DNA sequences (clusters or contigs), custom code was implemented to detect all possible open reading frames. Finally, translated protein sequences may be submitted as a multi-FASTA file; in the absence of any location information, all sequences will be considered part of the same biosynthetic cluster.

Once putative biosynthetic genes have been identified and translated to protein sequences, they are analysed with a library of hidden Markov models created from multiple sequence alignments of experimentally characterized enzymes relevant to nonribosomal peptide or polyketide biosynthesis. HMMER^61^ is used to perform hidden Markov model searches of the biosynthetic gene protein sequence database. Translated protein sequences are searched with adenylation, condensation and thiolation domain models obtained from PFAM^62^; dehydratase, enoylreductase, ketoreductase, acyltransferase and thioesterase domain models obtained from SMART^63^; and a *N*-methyltransferase domain model obtained from Ansari *et al*.^64^ Putative acyltransferase and adenylation domains are reanalysed using a library of profile hidden Markov models compiled by Khayatt *et al*.^13^ to determine their substrates.

Next, the annotated genes are grouped into putative biosynthetic clusters using a simple greedy algorithm with an adjustable window (set by default, to 10,000 base pairs). Putative clusters are discarded if they do not contain at least one adenylation or acyltransferase domain, and at least one condensation or ketosynthase domain. Within each cluster, biosynthetic modules are next defined. For adenylation modules, all domains between condensation and thiolation domains are considered a module if the intervening region contains an adenylation domain. For acyltransferase modules, all domains between ketosynthase and thiolation domains are considered a module if the intervening region contains an acyltransferase domain. A natural product scaffold is subsequently generated by considering the top-scoring substrate for each biosynthetic module and assumed co-linearity. Finally, the chemical reactions of polyketide reductive loops and *N*-methyltransferase domains are simulated on the scaffold to generate a predicted molecule for each cluster. The predicted natural product is output as a SMILES string, and then converted to a MDL Molfile to be loaded into the scaffold combinatorialization mode.

*Scaffold library generation*. The scaffold library generation web interface allows the user to enumerate combinatorial libraries based on chemical input. The scaffold input mode allows the user to submit a scaffold molecule or database of molecules for combinatorialization directly. Alternatively, the scaffold library generation interface will be automatically loaded on completion of a genome search, with each putative biosynthetic cluster's predicted natural product considered a scaffold. The interface allows users to define sites of variability and input R groups, and select at which sites their R groups may be combinatorialized. SmiLib^25^ is used to enumerate the combinatorial library based on user-submitted input.

A complete guide to the scaffold library generator user interface is available at www.magarveylab.ca/gnp/#!/help.

*LC–MS/MS database-dependent search*. The LC–MS/MS database-dependent search interface implements considerably updated software and options based on those originally reported in Ibrahim *et al*.^26^. GNP includes several new cleavage options, including ester cleavage for depsipeptides and inverse ester and thioether cleavages for abnormal MS/MS cleavage patterns^65^. GNP also has the ability to generate images of each matched fragment or structure to facilitate matched fragment verification.

The prediction-guided discovery chart is generated by identifying all scans where GNP identifies a prediction library compound as the top-ranked hit. This top-ranked P1 value is then divided by the average P1 values of the dummy library (in-house NRP library of all other compounds) for that scan. Since eluting compounds will have multiple scans associated with them in a chromatogram, to identify true compounds, normalized P1 values are summed in 0.25-min buckets and plotted on the prediction-guided discovery chart.

The GNP platform is an Apache Tomcat web application written in Java, and relies on sequence conversion by BioJava^66^ and chemical abstractions developed by the CDK^67^.

*Identification of sugar biosynthesis enzymes*. To connect genomic sequence information to natural product glycosylation patterns, we first developed a library of hidden Markov models corresponding to sugar biosynthesis genes. Using the biosynthetic codes outlined by Kersten *et al*.^22^ as a guide, protein sequences representing 20 families of TDP-sugar biosynthesis were manually collected based on homology to experimentally annotated sequences (Supplementary Tables 9–10). These sequences were aligned using Clustal Omega (version 1.2.0), and hidden Markov models were generated from the resulting alignments using the hmmbuild programme (version 3.1b1), part of the HMMER software package^61^. Appropriate bitscore cutoffs were determined for each hidden Markov model by manual analysis of the results of a hidden Markov model search against the UniProtKB database, using the HMMER web server^61^. The resulting library of hidden Markov models, and the sequences used to construct them, are available at http://magarveylab.ca/sugars/.

The biosynthetic pathways for natural product pentose sugars, including the deoxyaminosugars of calicheamicin and AT2433, and the madurose moiety of maduropeptin, lack a 4,6-dehydratase. Instead, a UDP-glucose dehydrogenase catalyses the four-electron oxidation of glucose to glucuronic acid, followed by oxidative decarboxylation by a UDP-glucose decarboxylase^35^,^36^. To predict pentose sugar moieties from genomic DNA, hidden Markov models specific to these UDP-sugar pathways were additionally constructed.

Many natural products, including vancomycin, avilamycin and BE-7585A, contain both hexose sugars and their deoxygenated derivatives. However, hexose sugars are primary metabolites, and consequently genes associated with hexose biosynthesis are scattered throughout microbial genomes. To develop a strategy to identify hexose sugars from genomic sequence information, we performed a phylogenetic analysis of glycosyltransferase domains (Supplementary Fig. 17). A database of glycosyltransferase domain sequences was manually curated, and each sequence was associated with a sugar substrate. The resulting 82 natural product glycosyltransferase sequences were aligned in MUSCLE (version 3.8.31) and manually refined and masked in Mesquite (version 2.75) at the amino-acid level. A phylogenetic tree was created in RAxML (version 7.4.2), performing 100 bootstraps with a gamma distribution. Maximum likelihood analyses were based on the LG substitution model with empirical base frequencies. The resulting tree was rendered in Figtree (version 1.4.0). Phylogenetic analysis revealed that natural product glycosyltransferases specific to hexose sugars assort into distinct clades in a phylogenetic tree. The glycopeptide mannosyltransferases represent two distinct families of hexose glycosyltransferases, while BE-7585A type glucosyltransferases represent a third. One mixed clade, consisting of both hexose and deoxysugar glycosyltransferases, was observed. These are glycopeptide glycosyltransferases, which are position specific but promiscuous in substrate selection^68^.

These results supported the use of glycosyltransferase domain sequence homology to infer the presence of glucose, mannose, gulose or *N*-acetylglucosamine within a natural product structures. A hidden Markov model was constructed from the glycosyltransferase sequence database using the methods described above. The unaligned sequences were also used to compile a BLAST database. Within GNP, glycosyltransferase domains identified using the hidden Markov model are queried against the BLAST database, and the highest-scoring result is used to determine whether the substrate of a given glycosyltransferase is a hexose sugar.

*Prediction of glycosylated natural product scaffolds*. To connect identified sugar biosynthesis genes to deoxysugar monomers, it was necessary to catalogue the biosynthetic pathways of deoxysugar biosynthesis. The biosynthetic logic developed Kersten *et al*.^22^ was expanded to include UDP-sugar pathway genes, and modified in cases where an insufficient number of sequences were available to build a hidden Markov model. The structures of each of the resulting 67 sugars were compiled as a set of SMILES strings. The amended biosynthetic code, consisting of 67 deoxysugars, their structures and the genes associated with their biosynthesis, is presented in Supplementary Table 10.

Individual sugar biosynthesis genes are common to diverse biosynthetic pathways, and multiple pathways are often present in a cluster. Moreover, separate biosynthetic pathways may not be physically segregated within a cluster. It was therefore necessary to implement new algorithmic logic to predict multiple deoxysugars in a single biosynthetic gene cluster. Within GNP, the number of deoxysugars present in the natural product scaffold is calculated by subtracting the number of glycosyltransferases with hexose substrates from the total number of glycosyltransferases in the cluster. All combinations of the 67 sugars of this size are then computed. For each combination of sugars, both the number of genes present in the cluster but absent from the sugar biosynthesis pathways, and the number of genes present in the sugar biosynthesis pathways but absent from the cluster, are simultaneously minimized. The set of sugars that minimize both sums is retained and presented to the user as the genomically identified combinatorial sugar set. Hexose sugars identified based on glycosyltransferase homology are then added to this set. The predicted sugars are then added to a random hydroxyl group within the predicted natural product scaffold subsequent to user-directed structure combinatorialization. The sugar prediction algorithm is represented by the pseudocode shown in Supplementary Note 2.

*GNP analysis of *S. calvus**. Mining of in-house-sequenced *Streptomyces* genomes revealed a novel nonribosomal peptide biosynthetic gene cluster in *S. calvus* (ATCC No. 13382). The corresponding gene cluster was uploaded to the GNP server to generate an automated structure prediction. The presence of two unusual *trans*-acting adenylation domains led to position swapping for Asn6-Thr7 (and vice versa). In addition, the presence of an initiating condensation domain indicated acylation of the N-terminal amine, indicating a cyclic lipodepsipeptide or linear lipopeptide structure. For combinatorialization, the initiating amine was modified with a C_8_, C_10_ or C_12_ fatty acid. Serine and threonine, valine and leucine/isoleucine, and phenylalanine/tyrosine were considered to be interchangeable. The four combinatorialized scaffolds yielded a library of 768 hypothetical structures. LC–MS/MS data were probed with GNP using an 18-Da precursor window and minimal *P* score cutoffs (*P*1=10; *P*2=10), with 1-2 amide cleavages, 0-1 ester cleavages and 0-1 water losses. The prediction-guided discovery chart was overlaid with the base-peak chromatogram to indicate compounds related to the novel biosynthetic gene cluster.

*GNP analysis of *A. citrulli* AAC00-1*. Bioinformatic analysis of publicly available genome sequences revealed a number of orphan biosynthetic gene clusters, including a novel nonribosomal peptide biosynthetic gene cluster in *A. citrulli* AAC00-1 (DSM No. 17060). To generate an automated structure prediction, the novel gene cluster was uploaded to the GNP server. Extensive similarity observed between the novel biosynthetic gene cluster and that of the delftibactin biosynthetic gene cluster allowed for the correction of monomer incorporation at position 2, as ornithine rather than serine. In addition, the presence of an aspartate β-hydroxylating enzyme led to the conclusion that the predicted position 6 aspartate was β-hydroxyaspartate. This structure was combinatorialized to generate the linear and peptide macrocycle forms, with serines and threonines being interchangeable, the polyketide portion incorporating either a malonate or methylmalonate, and ornithines with side chains either as free amines, *N*-hydroxylated, *N*-formylated, *N*-acetylated or any combination thereof, yielding a library of 576 hypothetical structures. LC–MS/MS data were probed with GNP using a 50-Da precursor window and no *P* score cutoffs (*P*1=0; *P*2=0), with 1-2 amide cleavages, 0-1 ammonia losses and 0-1 water losses. The prediction-guided discovery chart was overlaid with the base-peak chromatogram to indicate compounds related to the novel biosynthetic gene cluster.

*GNP analysis of *V. paradoxus* S110*. Bioinformatic analysis of the publicly available genome of *V. paradoxus* S110 revealed an orphan biosynthetic gene cluster with considerable homology to that previously explored in *A. citrulli* AAC00-1. To test whether improvements in the structure-prediction abilities of GNP would improve detection of related scaffolds in a novel background, the *V. paradoxus* biosynthetic gene cluster was uploaded to the GNP server to generate an automated structure prediction. The scaffold was combinatorialized similarly to the scaffold of acidobactin, albeit with the new FAAL substrate prediction. This structure library included the linear and depsipeptide macrocycle forms, with serines and threonines being interchangeable, the polyketide portion incorporating either a malonate or methylmalonate and ornithines with side chains either as free amines, *N*-hydroxylated, *N*-formylated, *N*-acetylated or any combination thereof, yielding a library of 576 hypothetical structures. Owing to increased confidence in the prediction and possibility of the novel ester bond, LC–MS/MS data were probed with GNP using a 50-Da precursor window and standard *P* score cutoffs (*P*1=20; *P*2=20), with 1-2 amide cleavages, 0-1 ester cleavages and 0-1 water losses. The prediction-guided discovery chart was overlaid with the base-peak chromatogram to indicate compounds related to the novel biosynthetic gene cluster.

*GNP analysis of *V. paradoxus* P4B*. Mining of in-house-sequenced genomes of lytic environmental organisms revealed a novel nonribosomal peptide biosynthetic gene cluster in *V. paradoxus* P4B. The corresponding gene cluster was uploaded to the GNP server to generate an automated structure prediction. The prediction was modified as the presence of an aspartate β-hydroxylating enzyme led to the conclusion that the predicted position 3 aspartate was β-hydroxyaspartate. This structure was combinatorialized to generate the linear lipopeptide and lipodepsipeptide macrocycle forms, with serines and threonines being interchangeable, the polyketide portion incorporating either a malonate or methylmalonate, and predicted *N*-hydroxyornithines being *N*-formylated, *N*-acetylated or not further modified, yielding a concentrated library of 32 hypothetical structures. LC–MS/MS data were probed with GNP using an 18-Da precursor window and minimal *P* score cutoffs (*P*1=10; *P*2=10), with 1-2 amide cleavages, 0-1 ester cleavages and 0-1 water losses. The prediction-guided discovery chart was overlaid with the base-peak chromatogram to indicate compounds related to the novel biosynthetic gene cluster.

*GNP analysis of *N. potens**. Analysis of the *N. potens* (DSM No. 45234) genome revealed a gene cluster for a glycosylated polyketide. The corresponding gene cluster was uploaded to the GNP server to generate an automated structure prediction. GNP indicated the presence of two deoxysugar gene clusters, predicted to encode machinery for producing D-angolosamine and either D-mycaminose or D-ravidosamine (which are diastereomers). For combinatorialization, the polyketide backbone was either macrocyclized on the distal hydroxyl or left linear. The linear polyketide was glycosylated with both angolosamine and mycaminose/ravidosamine, either sugar alone or neither. The lone free hydroxyl of the predicted macrolide was glycosylated with angolosamine or mycaminose/ravidosamine, or left bare. All methylmalonates were also made interchangeable with malonate, resulting in a library of 42 hypothetical structures. Given the comprehensive coverage of hypothetical polyketides, LC–MS/MS data were probed with GNP using a 1-Da precursor window and a reduced *P*1 cutoff to account for the minimal fragmentation observed with polyketides (*P*1=5; *P*2=20), with 0-1 ester cleavages, 0-2 sugar cleavages and 0-3 water losses to account for all plausibly predictable fragmentation. The prediction-guided discovery chart was overlaid with the base-peak chromatogram to indicate compounds related to the novel biosynthetic gene cluster.

*GNP analysis of *P. fluorescens**. Bioinformatic analysis of the in-house-sequenced *P. fluorescens* (DSM No. 11579) genome revealed a nonribosomal peptide biosynthetic gene cluster identical (∼90% sequence identity) to one found in *Pseudomonas sp.* SH-C52 (refs 18, 43), responsible to producing the cryptic natural product thanamycin. Automated structure predictions of both compounds yielded identical predictions, albeit with a *trans*-acting adenylation domain responsible for incorporation of 4-chlorothreonine (inferred by the presence of the conserved halogenase *thaC2*) working either at the N- or C-terminal condensation domain, depending on the position of the free-standing adenylation domain relative to the remaining genes for the NRPS assembly line. This structure prediction was modified based on known similarity to the syringomycin-type molecules, including incorporation of the chlorinated threonine, hydroxylation of the predicted aspartic acid, incorporation of the modified threonine (dehydrobutyrate) at position 7, modification with a 3-hydroxy fatty acid through the N-terminal acylating condensation domain and macrocyclization on the hydroxyl of the conserved and predicted serine. This modified prediction was combinatorialized based on substitutions with similar monomers as well as similar monomers from corresponding positions in syringomycin-type compounds (syringotoxin, syringostatin, syringomycin, pseudomycin and cormycin). Diaminobutyrate, ornithine or arginine was incorporated at position 2, aspartate or asparagine was used at position 3, serine or threonine/homoserine was used at position 4 and fatty acids used ranged from C12 to 16 with one or two hydroxyl groups, as seen on syringostatins, cormycin and the pseudomycins. The library of 120 hypothetical thanamycin structures was used to probe LC–MS/MS data with GNP using an 18-Da precursor window and reduced *P* score cutoffs (*P*1=15; *P*2=15), with 1-2 amide cleavages, 0-1 ester cleavages and 0-1 water losses. The prediction-guided discovery chart was overlaid with the base-peak chromatogram to indicate compounds originating from the thanamycin biosynthetic gene cluster. Identical parameters were used when scoring isotope-labelled thanamycin extracts.

*Generation of chemoinformatic tree of thanamycin structures*. To generate a chemoinformatic tree of predicted thanamycin structures, the combinatorialized library was assembled in SMILES format in a text file and submitted to the online interface of ChemMine Tools (http://chemmine.ucr.edu/tools/launch_job/Clustering/) for hierarchical clustering analysis. The resulting newick tree was rendered in Dendroscope and subsequently in Adobe Illustrator CS6.

*Identification of natural product standards*. Natural product 1,000 × stock solutions were prepared by dissolving 1 mg of pure standard in 1 ml of water, methanol or DMSO. 10 μl of 1,000 × stock was diluted into 1 ml of water to make 1 μg ml^−1^ working concentration solutions. A volume of 10 μl of each stock was injected for LC–MS/MS analysis using standard conditions, including a 10:1 flow splitter for MS acquisition. Samples were routinely visible under these conditions, except for capreomycin, which was only visible at 10 μg ml^−1^ working concentration. LC–MS/MS data files were converted to .mzXML files and analysed with GNP. Minimal *P* score cutoffs of 5 (*P*1) and 5 (*P*2) were implemented to account for minimal fragmentation and signal from the low-abundance standards. Fragmentation settings were as follows: daptomycin and thiostrepton—1-2 amide cleavage, 0-1 ester cleavage and 0-1 water loss; lincomycin—0-1 amide cleavage and 0-3 water loss; novobiocin—0-1 amide cleavage, 0-1 ester cleavage, 0-1 sugar cleavage and 0-2 water loss; nystatin—0-1 ester cleavage, 0-1 sugar cleavage and 0-3 water loss; erythromycin—0-1 ester cleavage, 0-2 sugar cleavage and 0-3 water loss; capreomycin, bacitracin, gramicidin and polymyxin—1-2 amide cleavage and 0-1 water loss; vancomycin—0-1 amide cleavage, 0-2 sugar cleavage and 0-1 water loss; valinomycin—0-2 amide cleavage, 0-2 ester cleavage and 0-1 water loss.

## Additional information

**Accession codes:** Genbank: The accession codes for the biosynthetic gene clusters for WS9326, variobactin and thanamycin are KT362217, KT362218 and KT362216. Other biosynthetic gene clusters can be found from publically available genomes.

**How to cite this article:** Johnston, C. W. *et al*. An automated Genomes-to-Natural Products platform (GNP) for the discovery of modular natural products. *Nat. Commun.* 6:8421 doi: 10.1038/ncomms9421 (2015).

## Supplementary Material

Supplementary Information Supplementary Figures 1-33, Supplementary Tables 1-17 and Supplementary Notes 1-2
