# Supplementary material for: An automated Genomes-to-Natural Products platform (GNP) for the discovery of modular natural products
Source: Nat Commun. 2015 Sep 28;6:8421. doi: 10.1038/ncomms9421 (PMC4598715; doi:10.1038/ncomms9421)
Supplement: Supplementary Information — Supplementary Figures 1-33, Supplementary Tables 1-17 and Supplementary Notes 1-2 [file ncomms9421-s1.pdf]

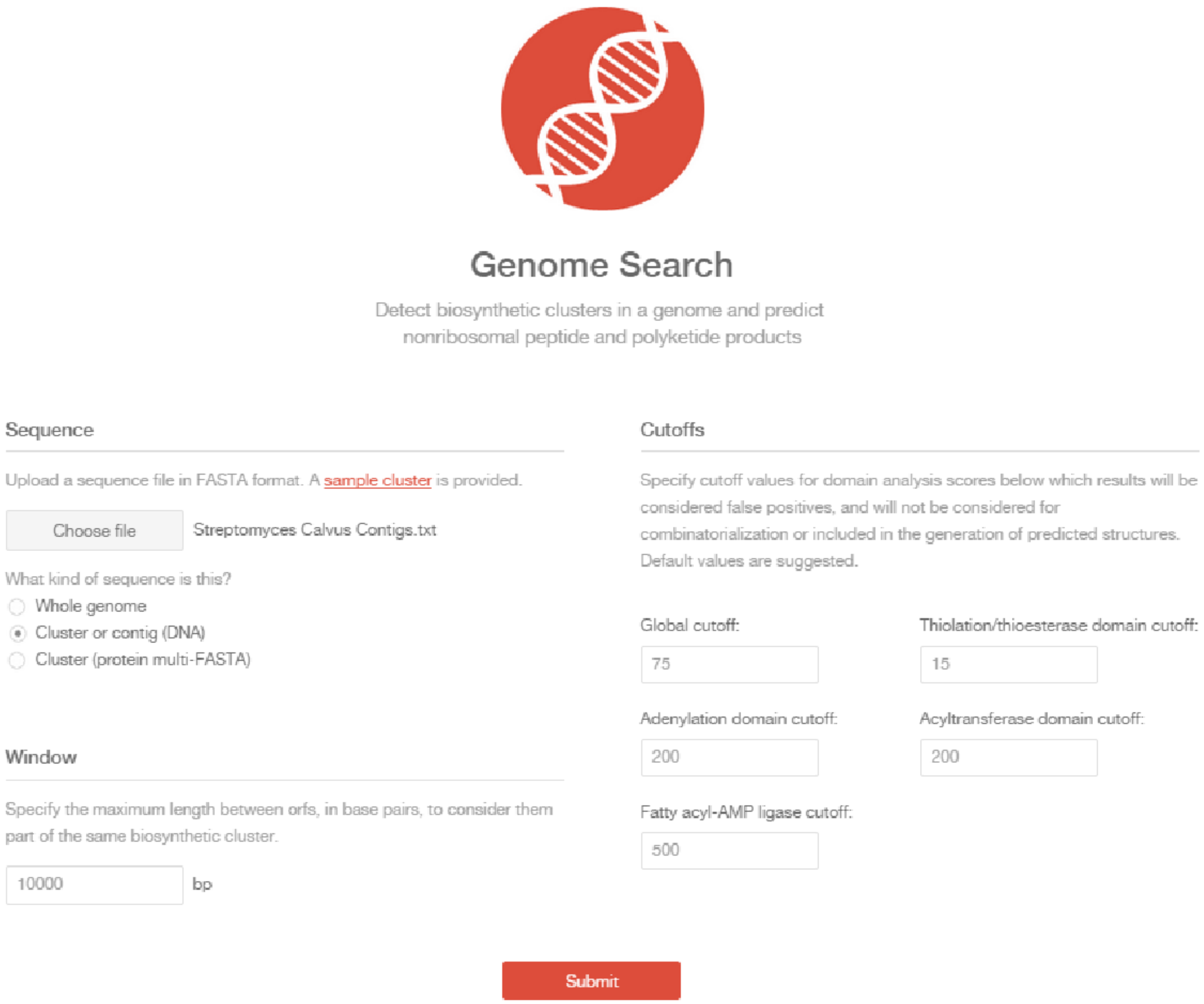

Supplementary Figure 1 GNP's genome search functionality**.** As a standard initiating step for the Genomes-to-Natural products Platform, users are prompted to upload gene sequence files in FASTA format. These genomes are analyzed by GNP, which detects PKS and NRPS gene clusters and generates predicted structures automatically.

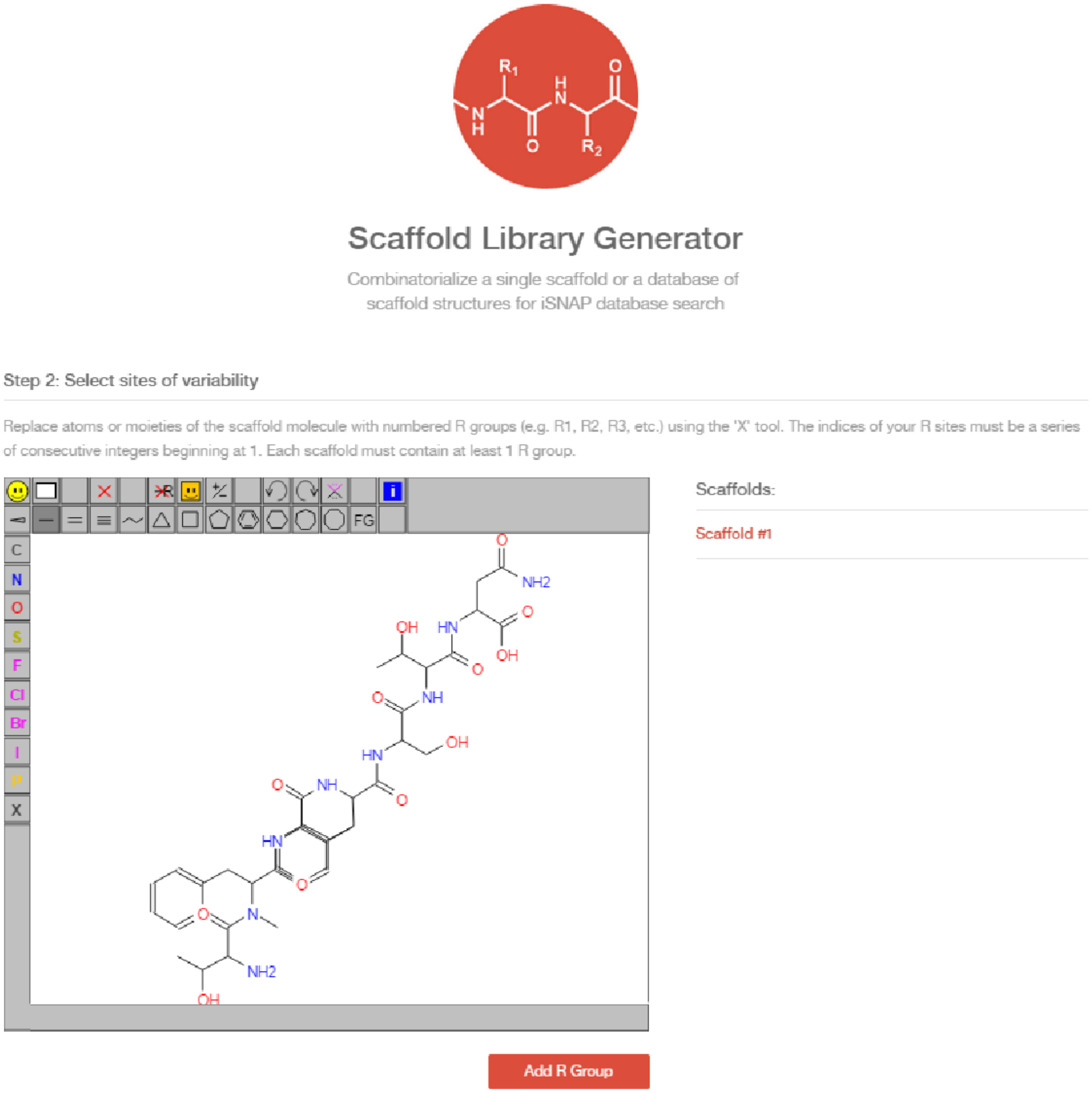

Supplementary Figure 2 GNP's scaffold library generator. Following either the automated generation of predicted scaffolds, or the uploading of a user-defined scaffold, predicted structures are combinatorialized at a number of sites defined by R-groups. Combinatorialized libraries are forwarded onwards for use in detecting predicted structures within LC-MS/MS data.

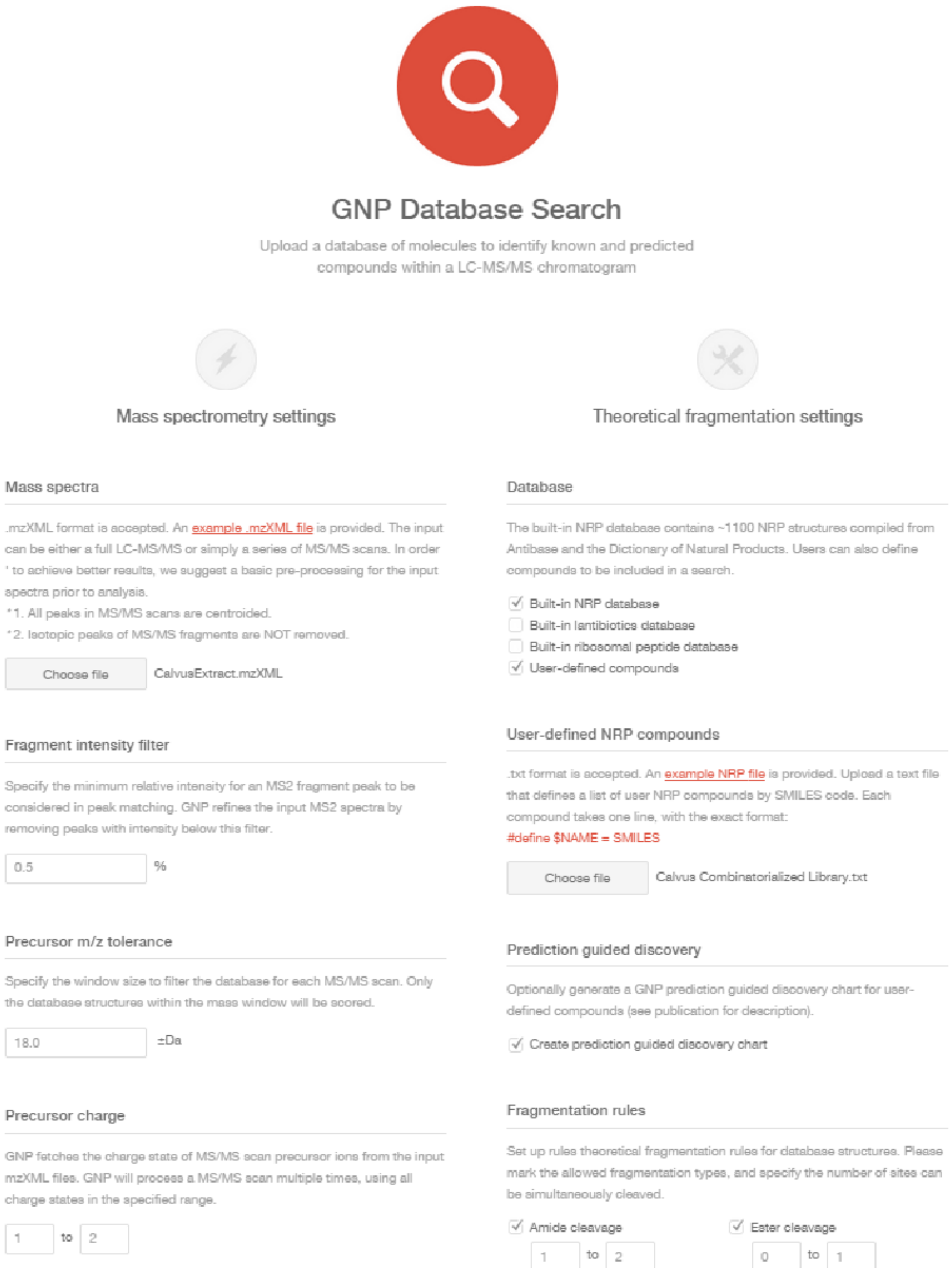

Supplementary Figure 3 GNP's database search functionality. Following the generation of a library of predicted structures, GNP can automatically detect compounds in relevant LC-MS/MS data. Uploaded mzXML files can be analyzed with predicted structure databases (user-defined compounds), using our established NRP database as a dummy to provide statistical scoring required for assessing hits. Various parameters can be adjusted based on the user confidence in the predicted structures, such as precursor m/z tolerance, which sets a window around a detected ion within which candidate predictions can be assessed for fragment ion matching.

Results

Your results will expire on December 21, 2014, and will be deleted.

Upload	Done.
Database Search	Generating report...<br>Report #3070 of 3070
Reports	Download reports:<br>Download NRP search report<br>Download matched fragment report

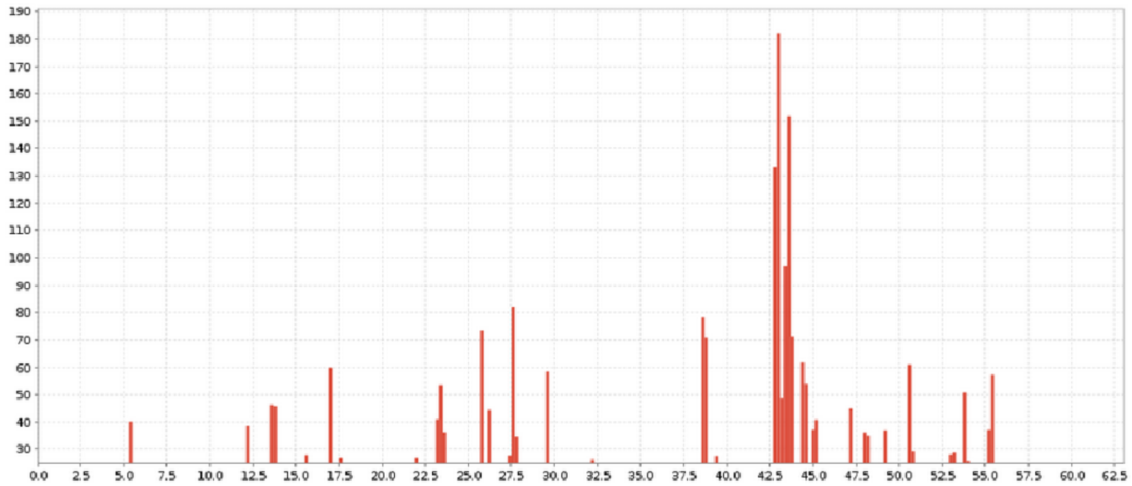

Click labels to sort.

Scan No.	RT	Precursor M/Z	Precursor Charge	Precursor Mass	Result	SMILES ↓	Mass	Matched Peaks	P1	P2
1088	17.33	508.51	2	1015.01	user_#define 1.1_2.1_4.1_6.1_7.1_4.1_5.1_5 1	C%13(C(C(NC(C(NC(C(NC(C%16O)C(NC(C(O)=O)CC(N)=O)=O)=O)C%15O)=O)CC1=CC=C%14C=C1)=O)NC(C(N(C(C(C%11(O))NC%10=O)=O)C)CC2=CC=C%12C=C2)=O)C.C%10CCCCCCCC.[H]%11.O%12.C%13C.[H]%14.C%15.C%16	1012.55	10	13.4	25.6

Supplementary Figure 4 Results of GNP LC-MS/MS analysis**.** GNP analysis of LC-MS/MS data using a library of predicted structures yields a prediction guided discovery chart which indicates the frequency of user defined compounds identified as top-scoring hits over time. These compounds are also listed below in an Excel-compatible spreadsheet listing the relevant scan, retention time, precursor charge and mass, as well as the SMILES code and mass for the predicted structure, the number of matched fragment ions, and the statistical parameter scores. Structures of parent and fragment ions are also viewable online by moving the cursor over the relevant SMILES code, though this option must be selected in database search settings.

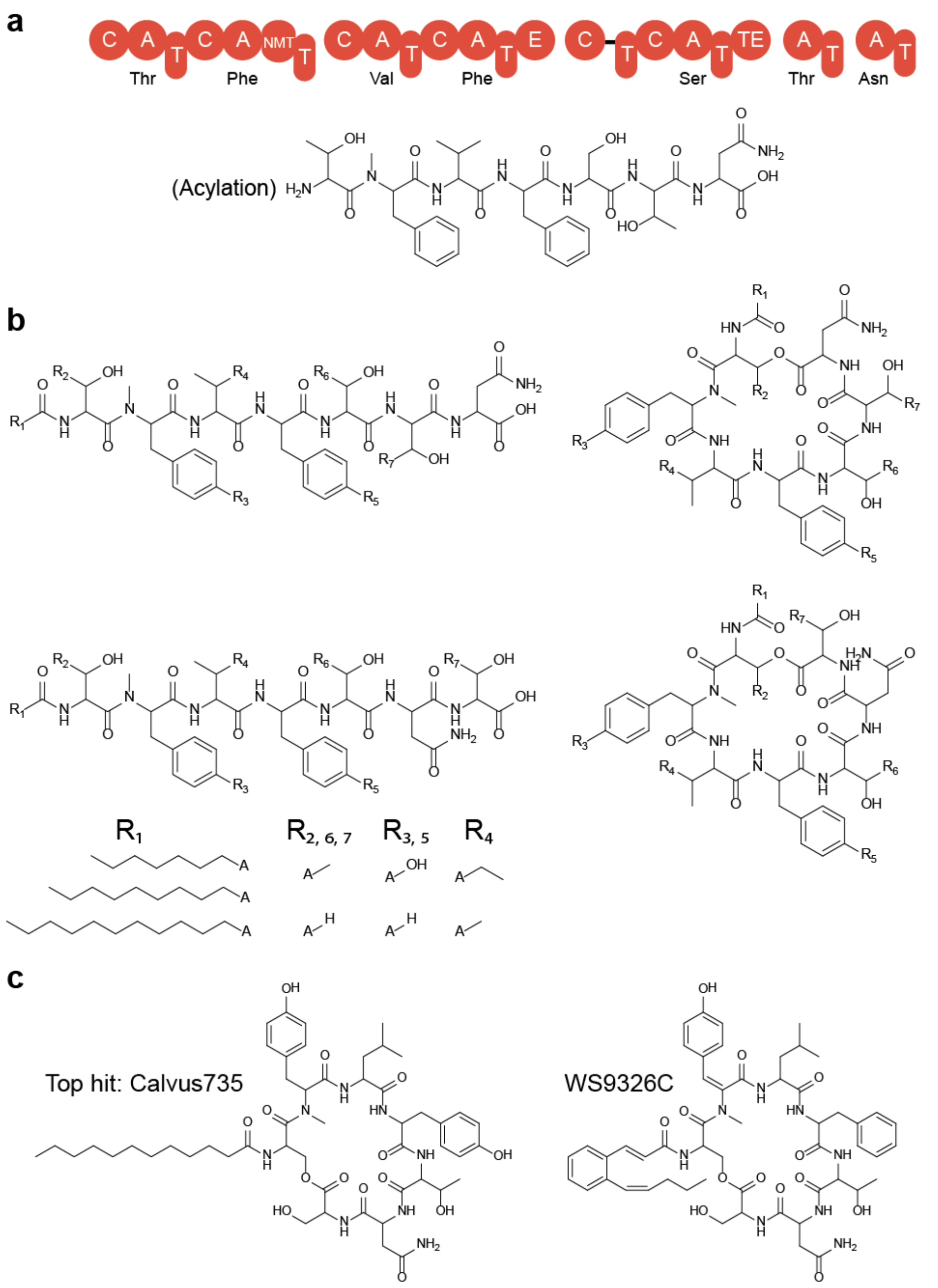

Supplementary Figure 5 Prediction, combinatorialization, and detection of WS9326 metabolites. (**a**) GNP detected NRPS biosynthetic gene cluster from *Streptomyces calvus*, with predicted monomers listed and GNP-generated prediction shown. (**b**) Structures used for combinatorialization, including varied order of the two trans-acting A-T didomains. (**c**) Structure of the most frequent hit detected within the *S. calvus* LC-MS/MS data (*left*), as well as the true associated metabolite, WS9326C (*right*).

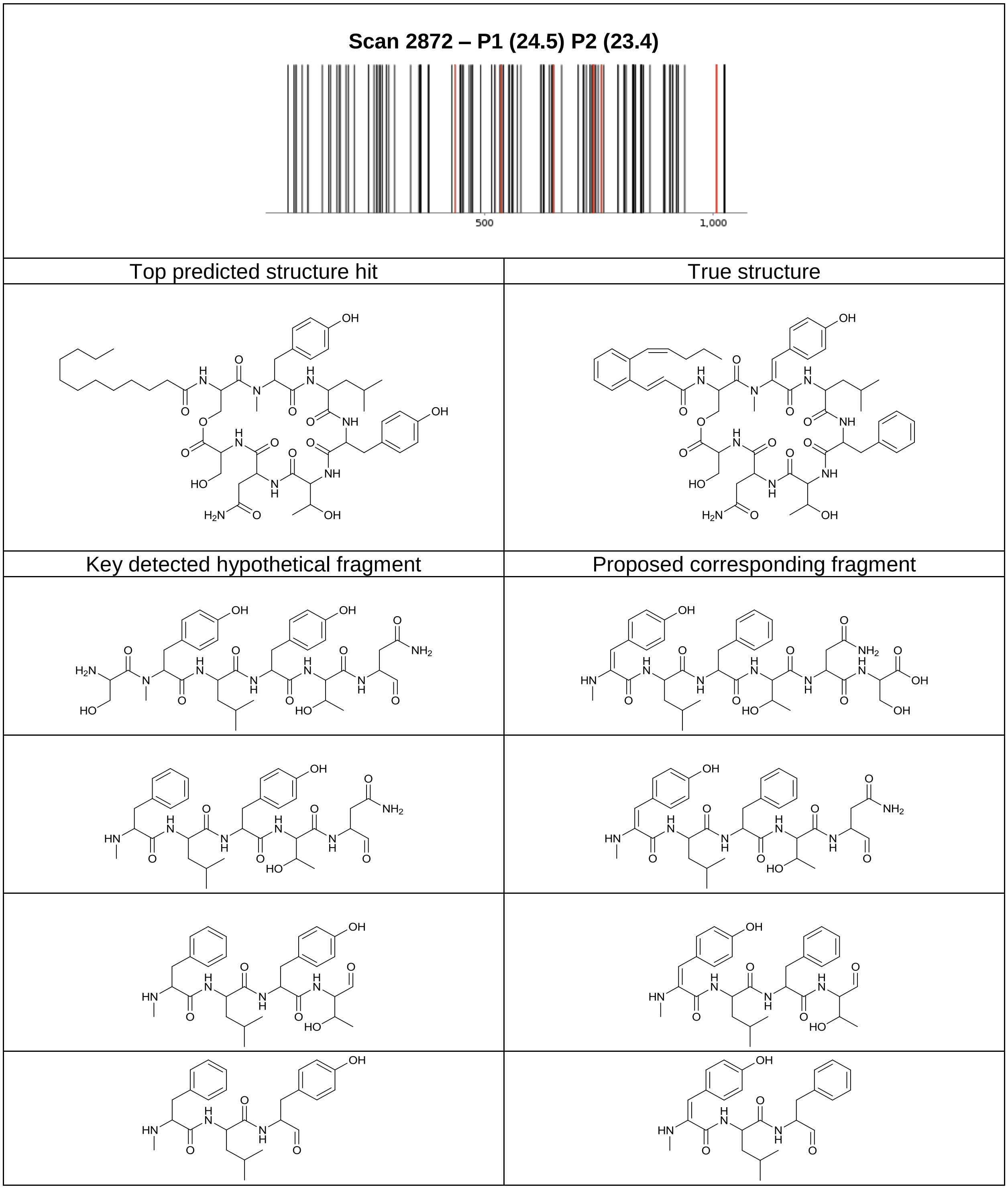

Supplementary Figure 6 Sample matched fragments between a predicted structure hit and WS9326C. Several matched fragments between the hypothetical compound hit and the corresponding true structure are shown. Fragments of WS9326C were generated using the GNP Fragmenter application. Detected hypothetical spectral fragments are shown as lines in the diagram above, with detected masses shown in red. P-scores for the scan are listed above. Full results tables for this scan and experimental run are available at http://magarveylab.ca/data/gnp.

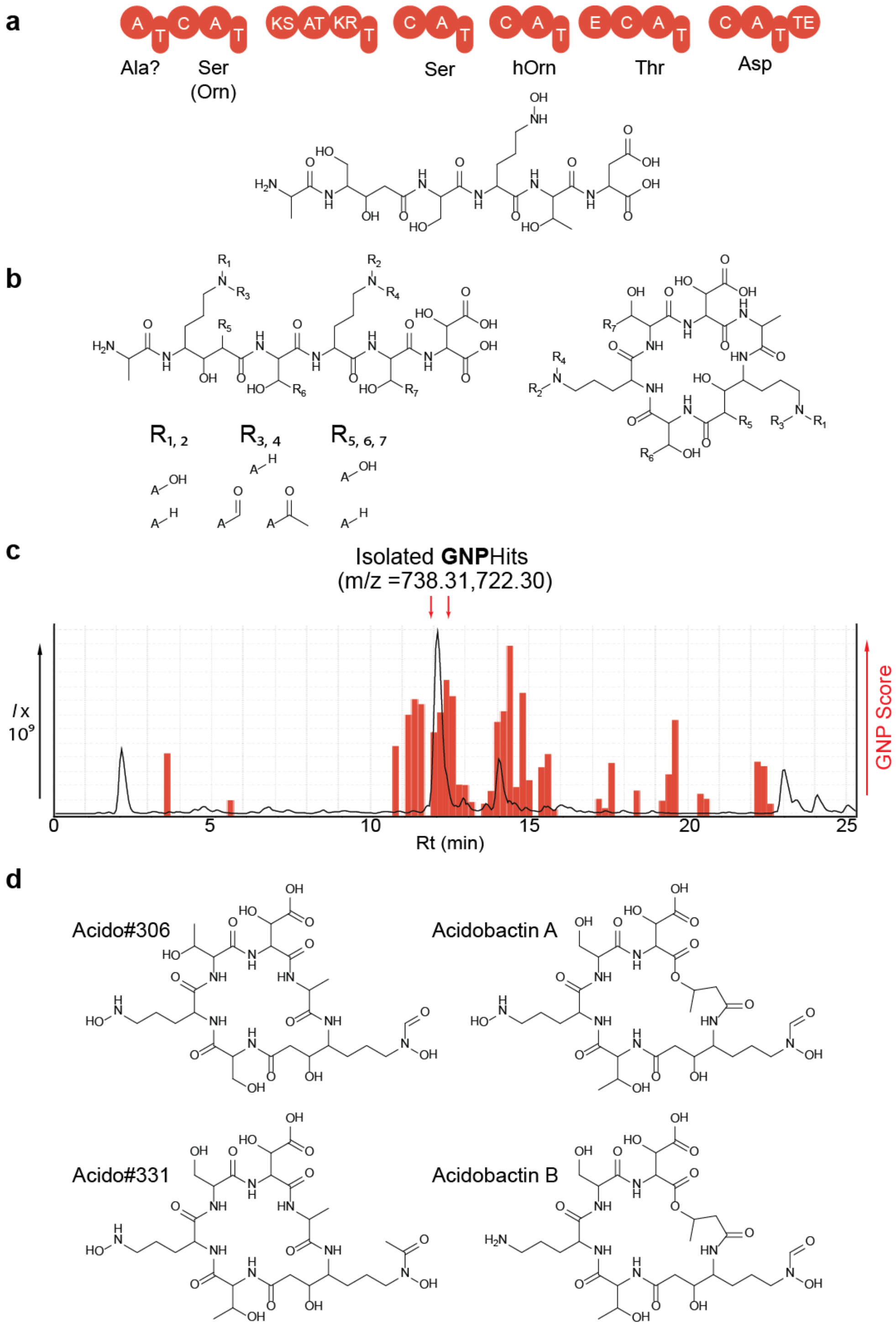

Supplementary Figure 7 Prediction, combinatorialization, and detection of acidobactin metabolites. (**a**) GNP detected NRPS biosynthetic gene cluster from *Acidovorax citrulli*, with predicted monomers listed and GNP-generated prediction shown. (**b**) Structures used for combinatorialization, taking into account the anticipated ornithine incorporation at position 2. (**c**) Prediction guided discovery chart indicating detected natural products related to the combinatorialized structure library. (**d**) Structures of the most frequent hits associated with the major metabolites from the *A. citrulli* LC-MS/MS data (*left*), as well as the true associated metabolites, acidobactin A and B (*right*).

a

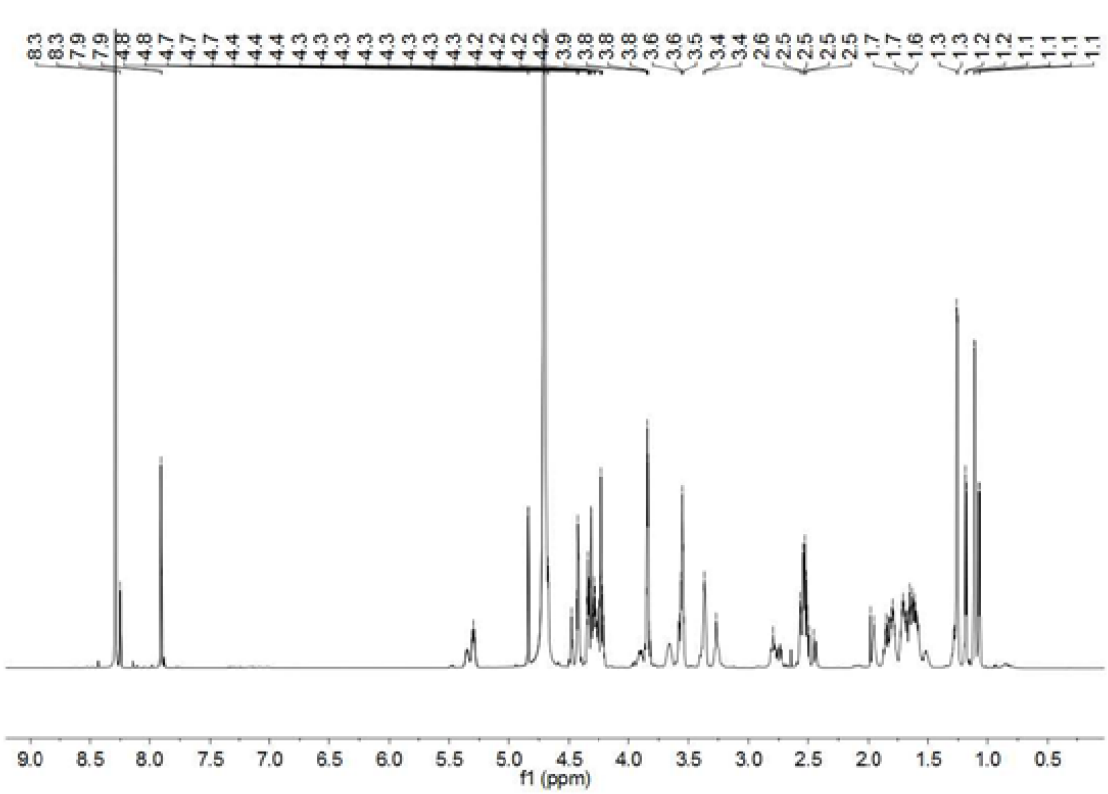

b

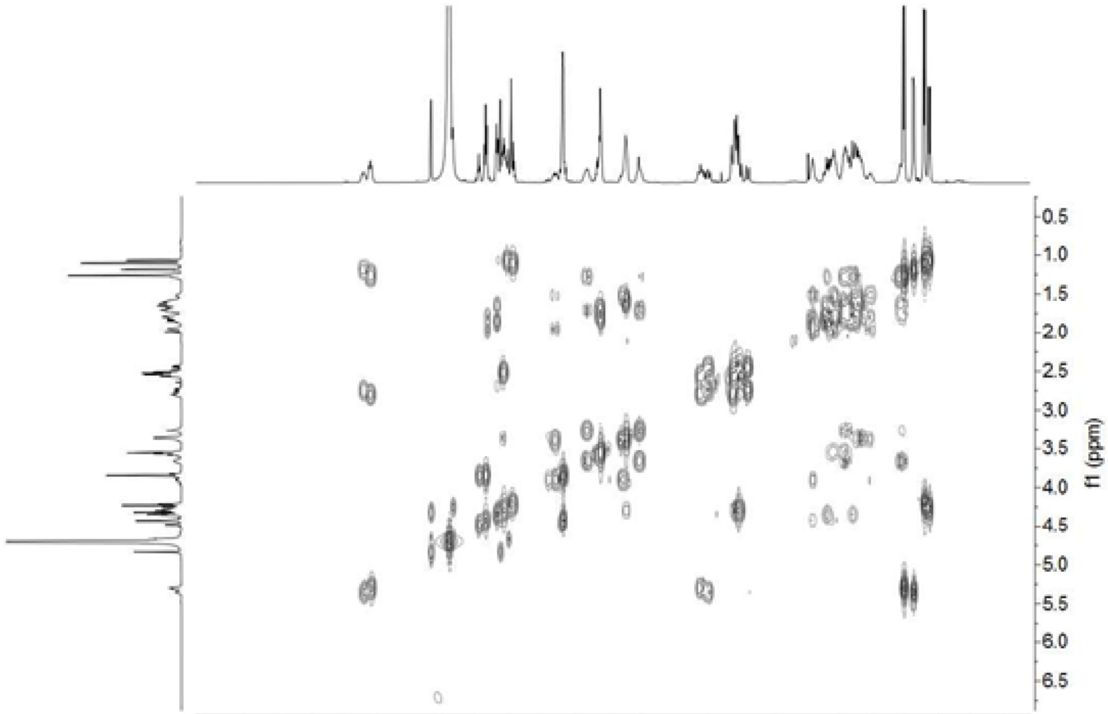

c

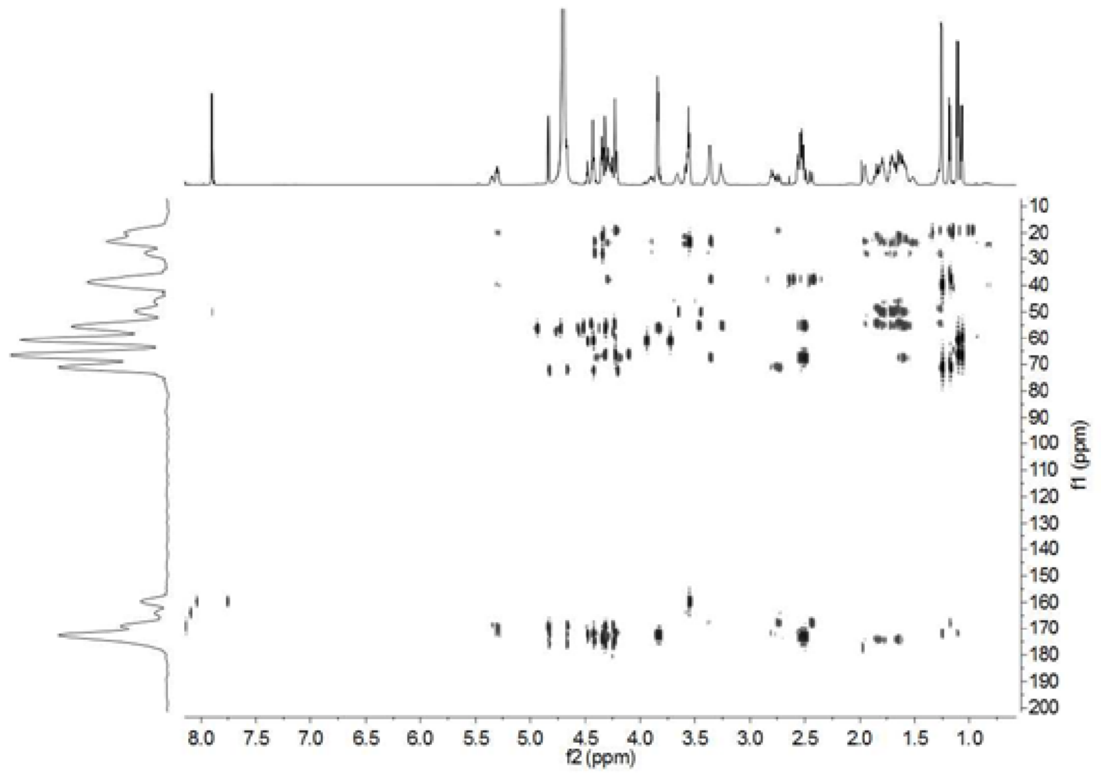

d

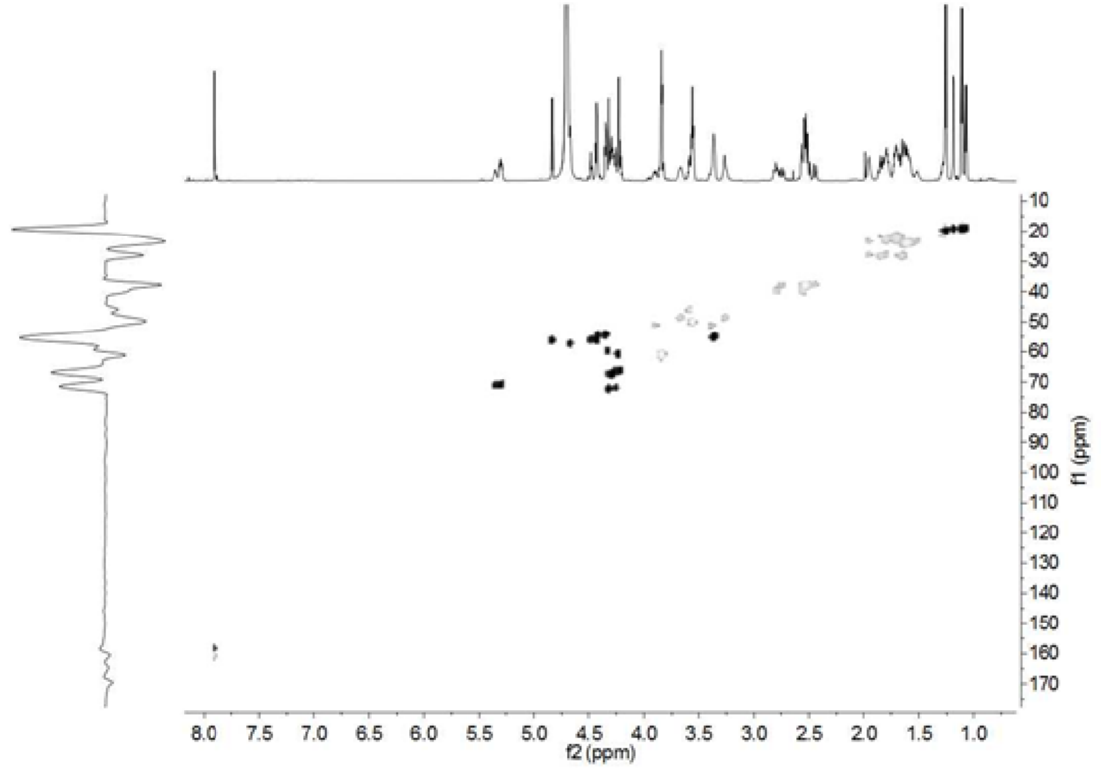

Supplementary Figure 8 NMR analysis of acidobactin A (**2**) in D_2O. (**a**) ^{1}H NMR spectrum. (**b**) ^{1}H-^{1}H COSY spectrum. (**c**) ^{1}H-^{13}C HMBC spectrum. (**d**) ^{1}H-^{13}C HMQC spectrum.

a

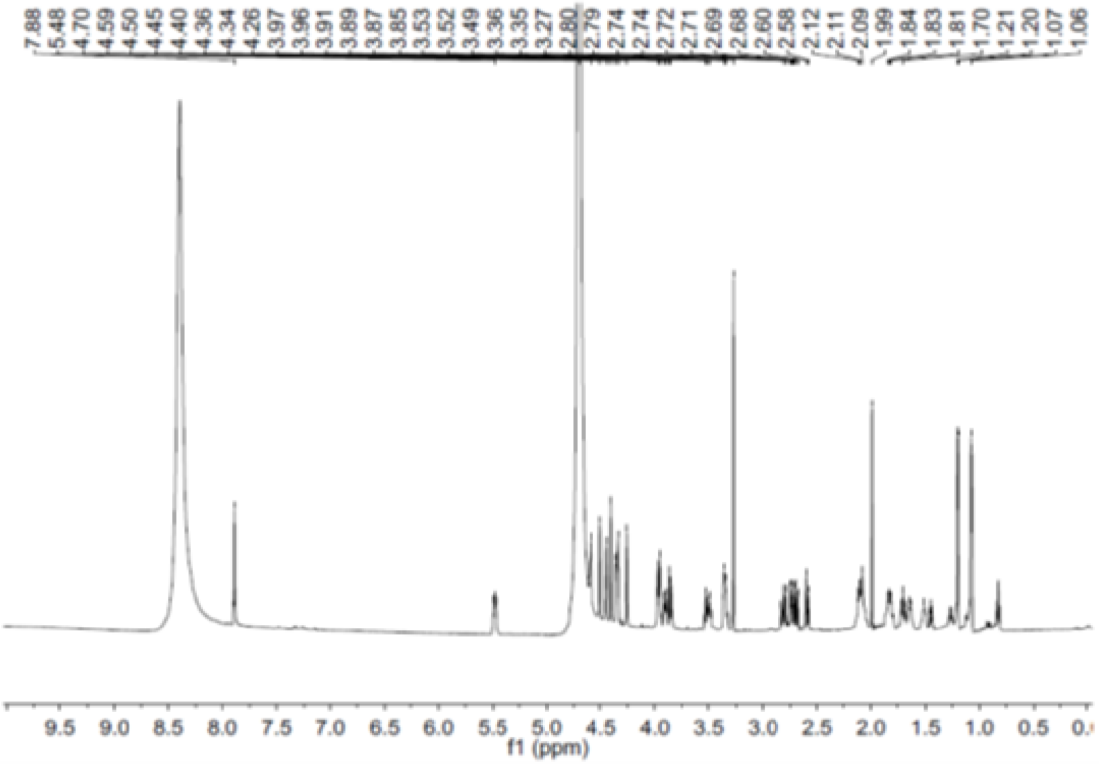

b

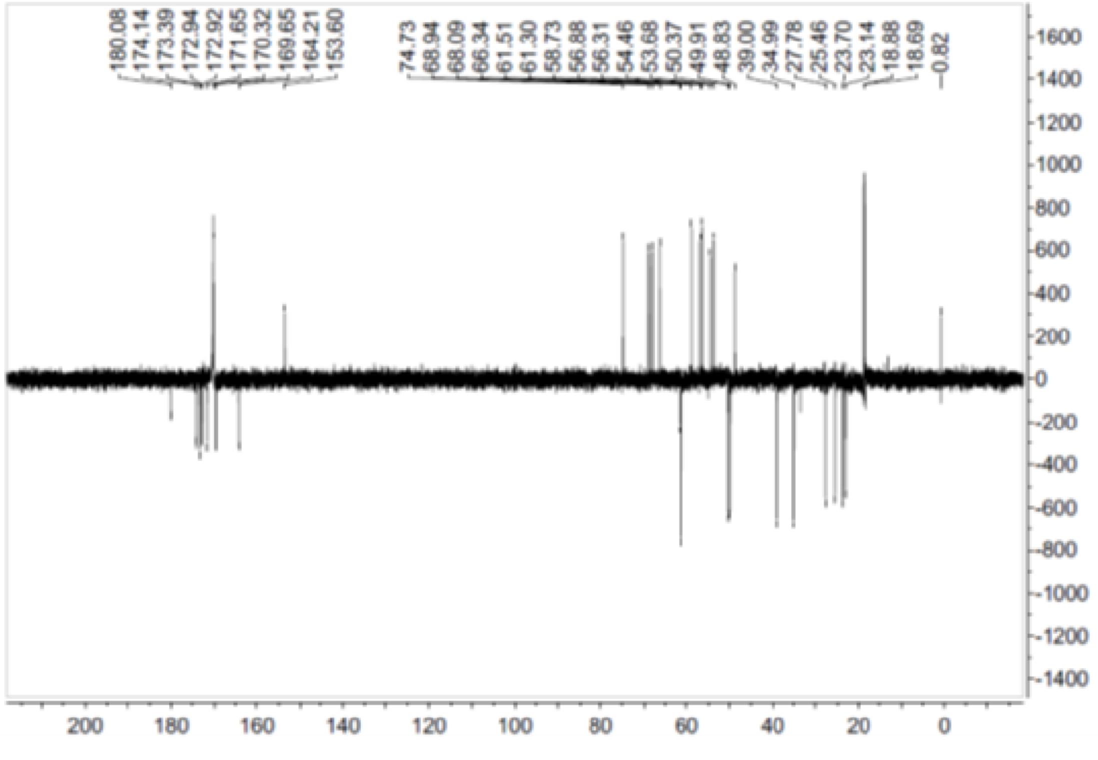

c

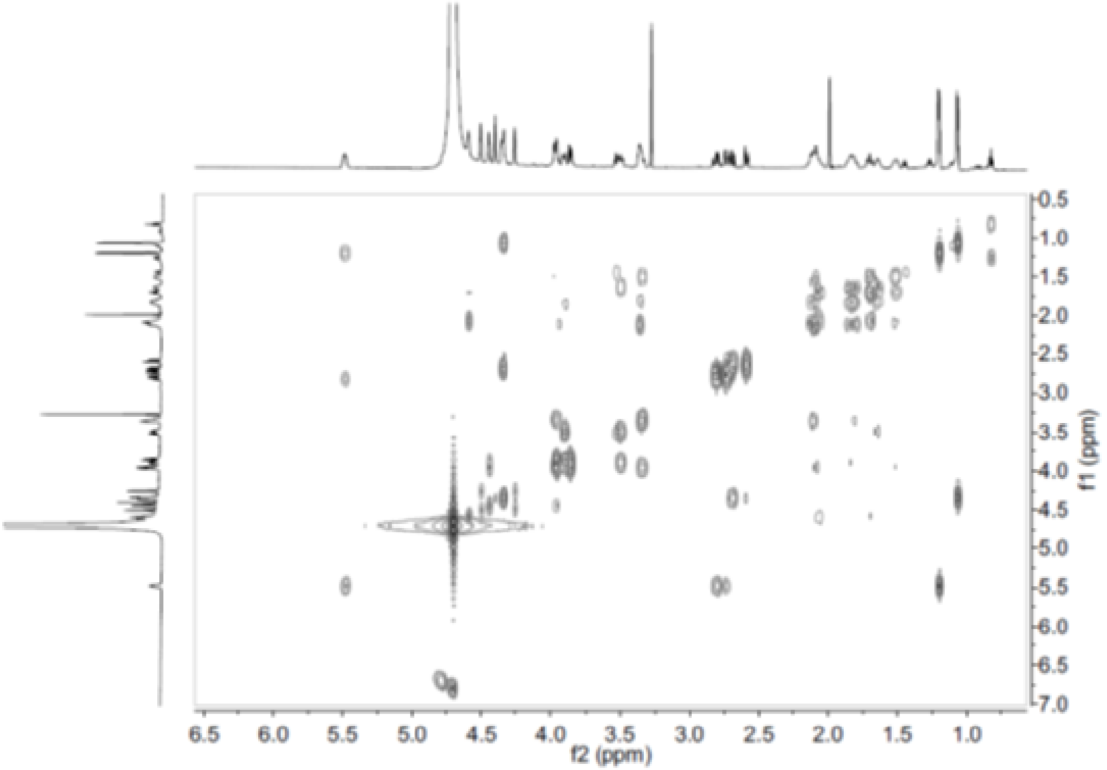

d

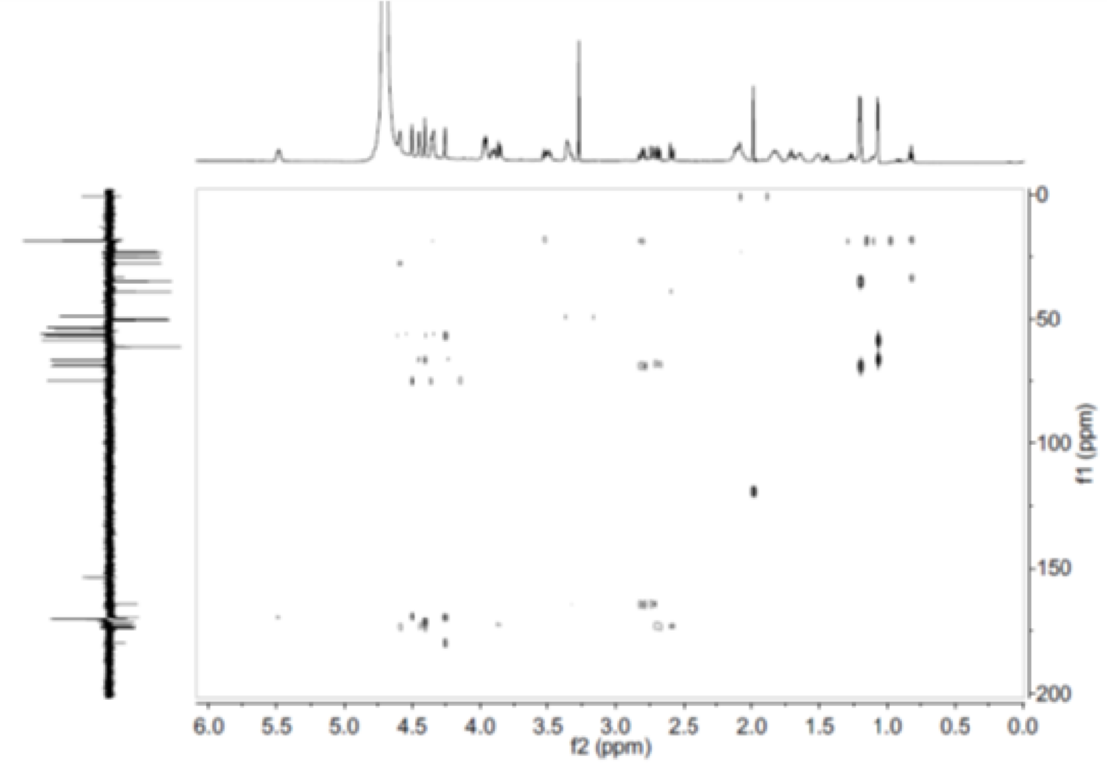

e

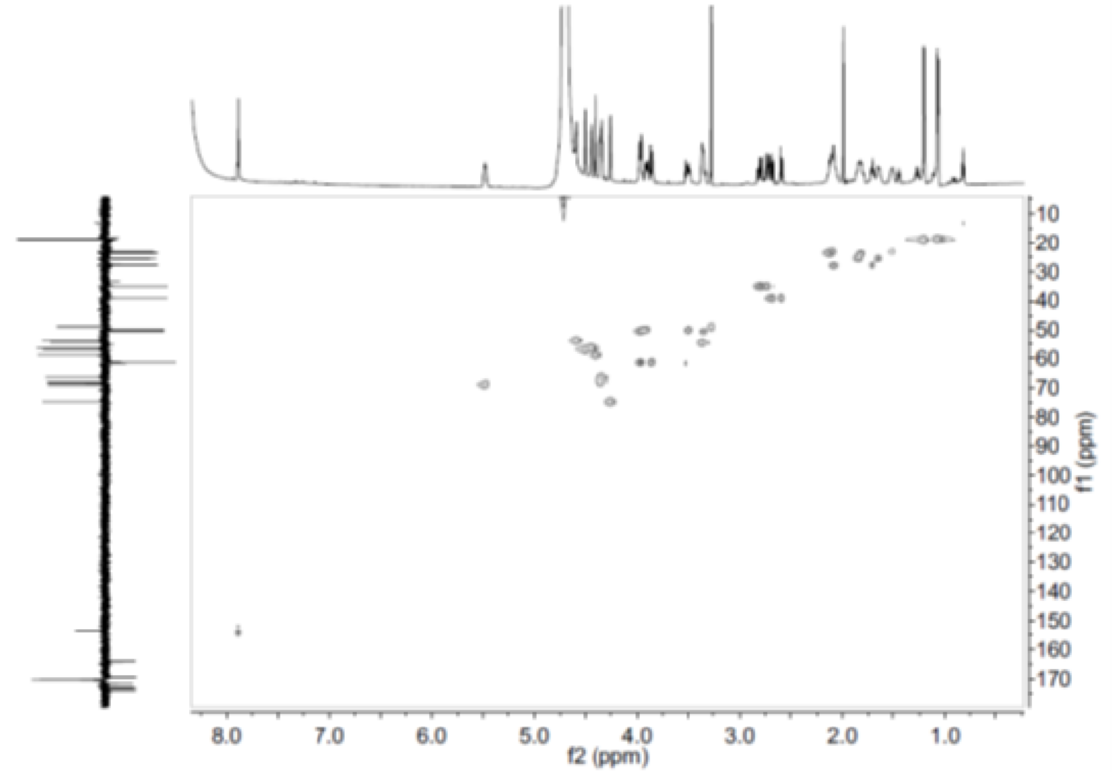

f

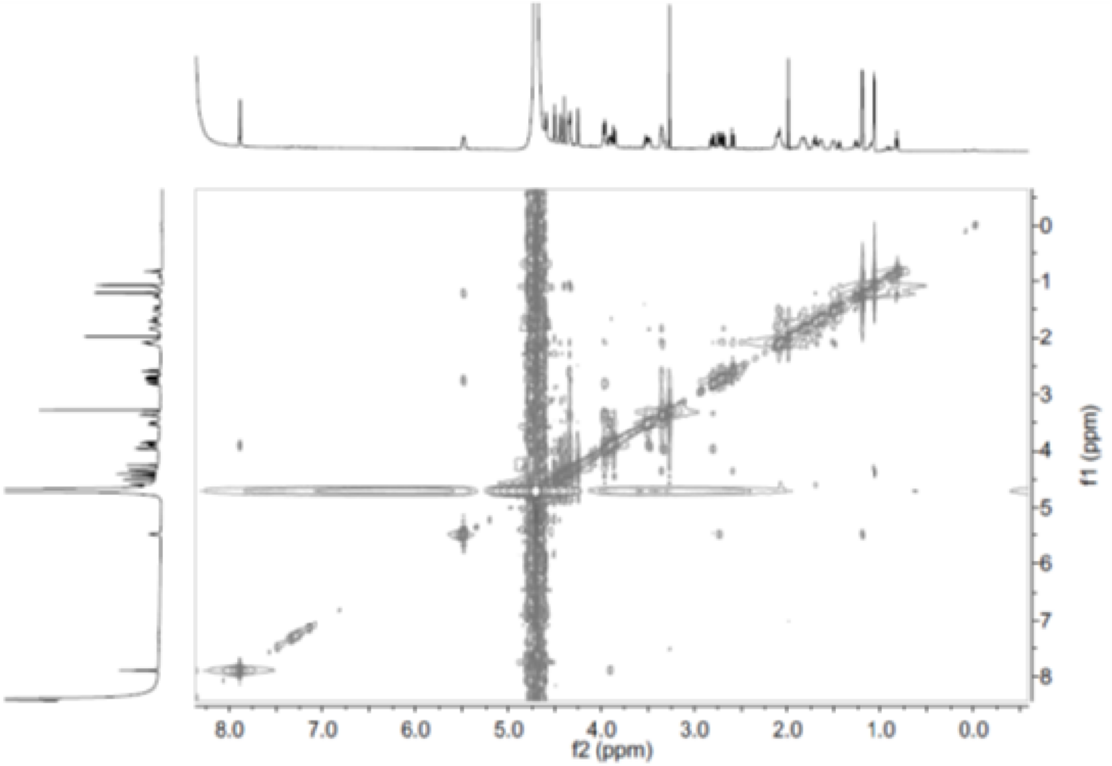

Supplementary Figure 9 NMR analysis of acidobactin B (**3**) in D_2O. (**a**) ^{1}H NMR spectrum. (**b**) ^{13}C DEPTq spectrum. (**c**) ^{1}H-^{1}H COSY spectrum. (**d**) ^{1}H-^{13}C HMBC spectrum. (**e**) ^{1}H-^{13}C HMQC spectrum. (**f**) ^{1}H-^{1}H ROESY spectrum.

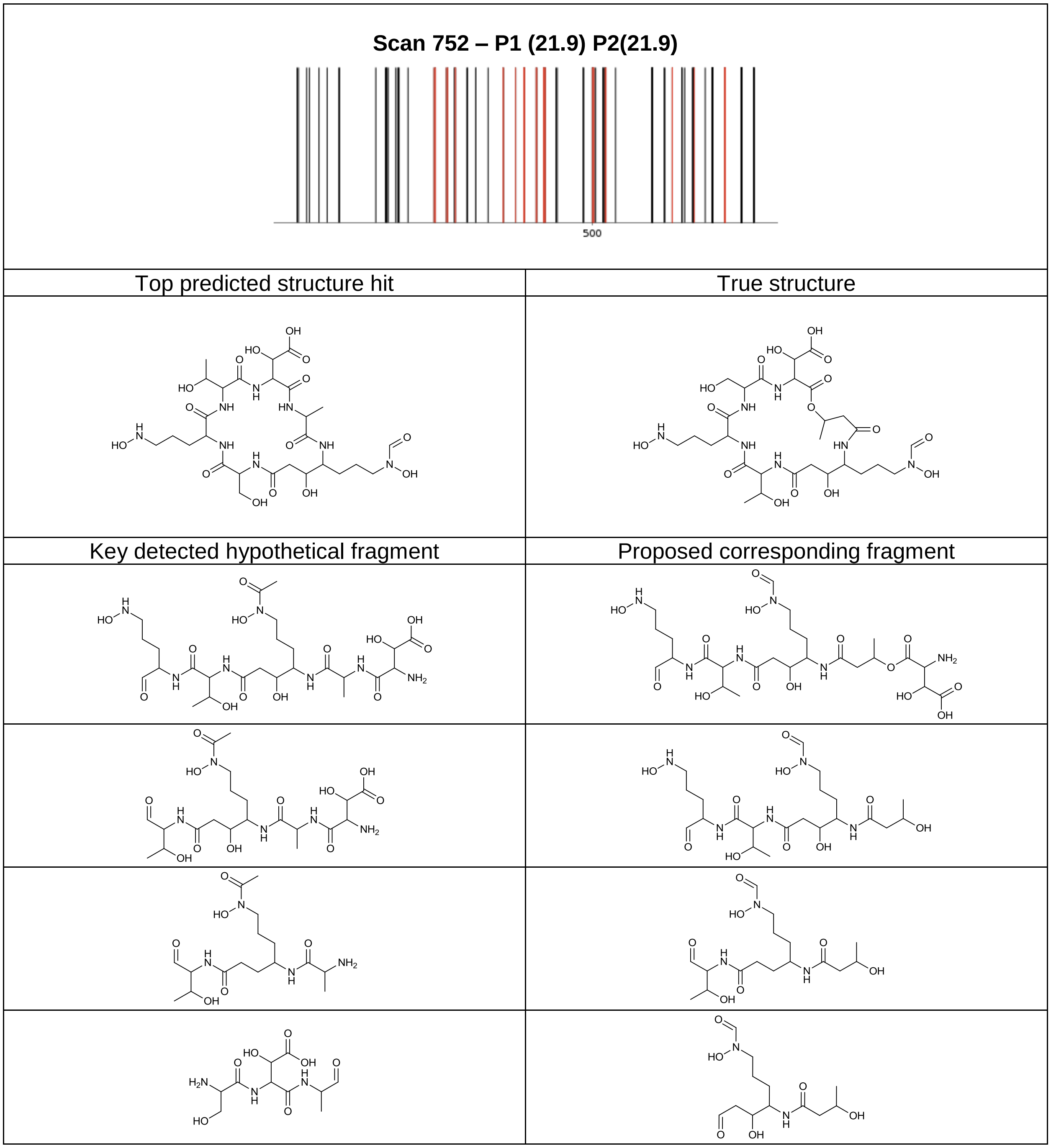

Supplementary Figure 10 Sample matched fragments between a predicted structure hit and acidobactin A. Several matched fragments between the hypothetical compound hit and the corresponding true structure are shown. Fragments of acidobactin A were generated using the GNP Fragmenter application. Detected hypothetical spectral fragments are shown as lines in the diagram above, with detected masses shown in red. P-scores for the scan are listed above. Full results tables for this scan and experimental run are available at http://magarveylab.ca/data/gnp.

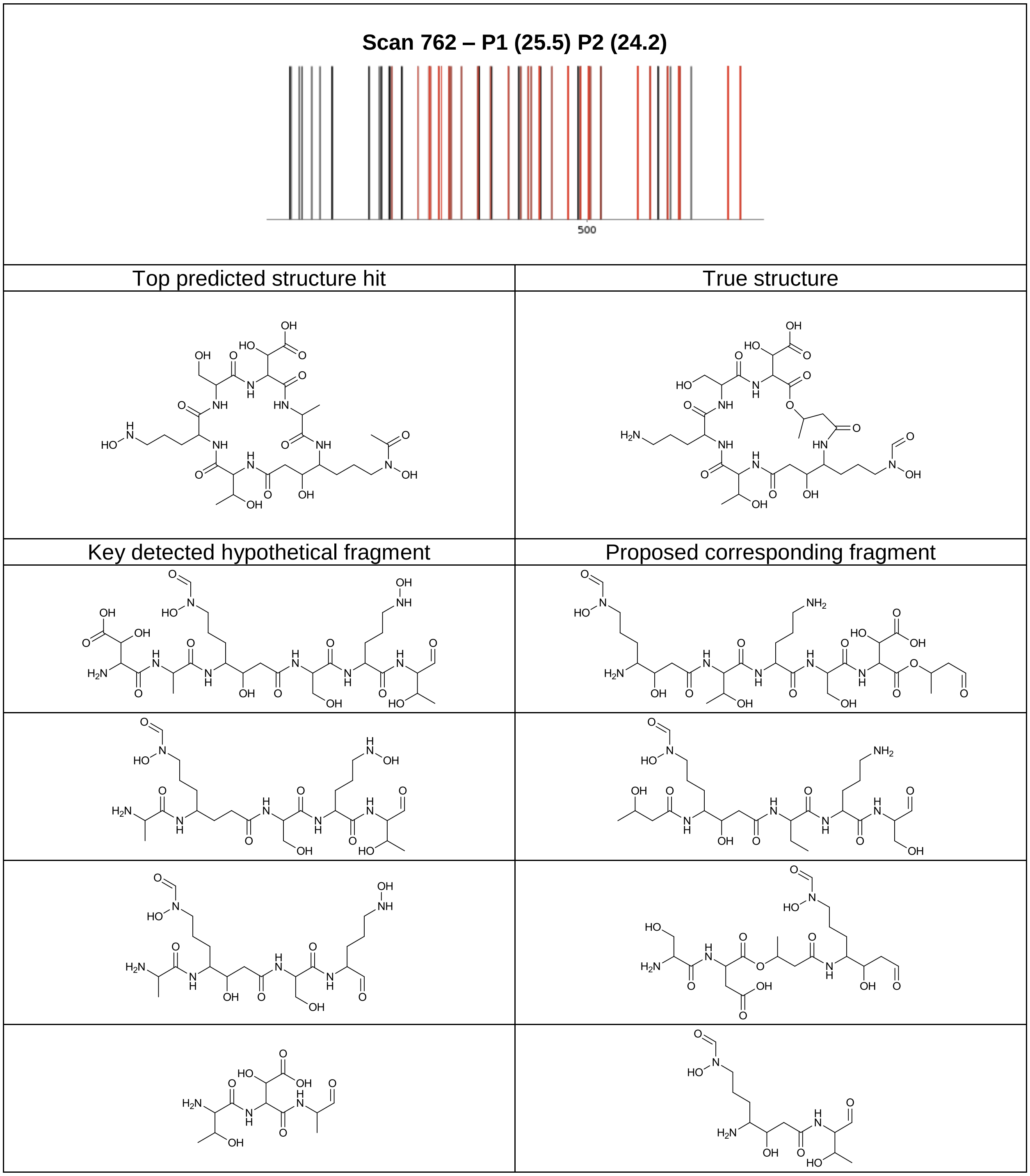

Supplementary Figure 11 Sample matched fragments between a predicted structure hit and acidobactin B. Several matched fragments between the hypothetical compound hit and the corresponding true structure are shown. Fragments of acidobactin B were generated using the GNP Fragmenter application. Detected hypothetical spectral fragments are shown as lines in the diagram above, with detected masses shown in red. P-scores for the scan are listed above. Full results tables for this scan and experimental run are available at http://magarveylab.ca/data/gnp.

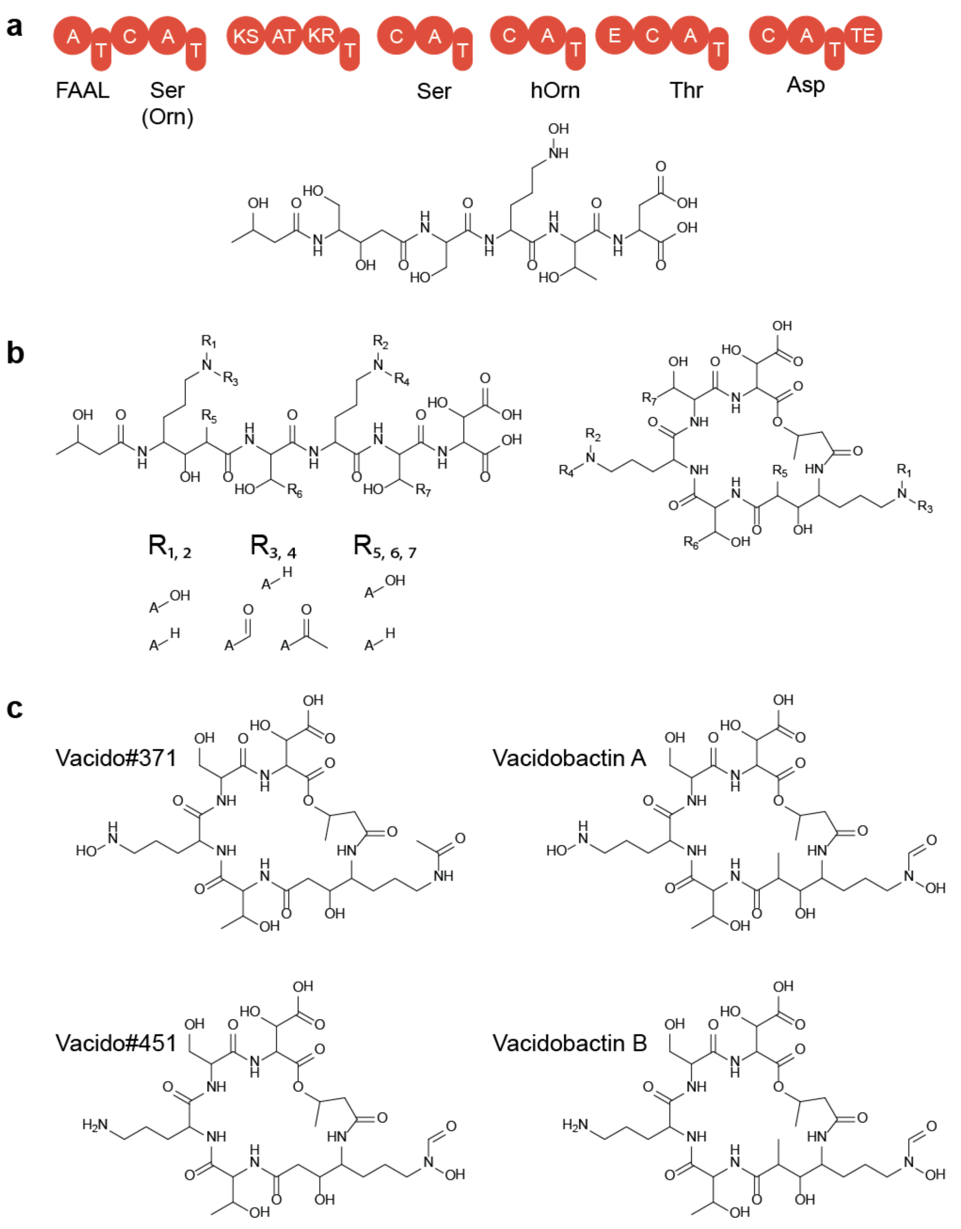

Supplementary Figure 12 Prediction, combinatorialization, and detection of vacidobactin metabolites. (**a**) GNP detected NRPS biosynthetic gene cluster from *Variovorax paradoxus* S110, with predicted monomers listed and GNP-generated prediction shown. (**b**) Structures used for combinatorialization, taking into account the anticipated ornithine incorporation at position 2 (**c**) Structure of the most frequent hits detected within the *V. paradoxus* S110 LC-MS/MS data (*left*), as well as the true associated metabolites, vacidobactin A and B (*right*).

a

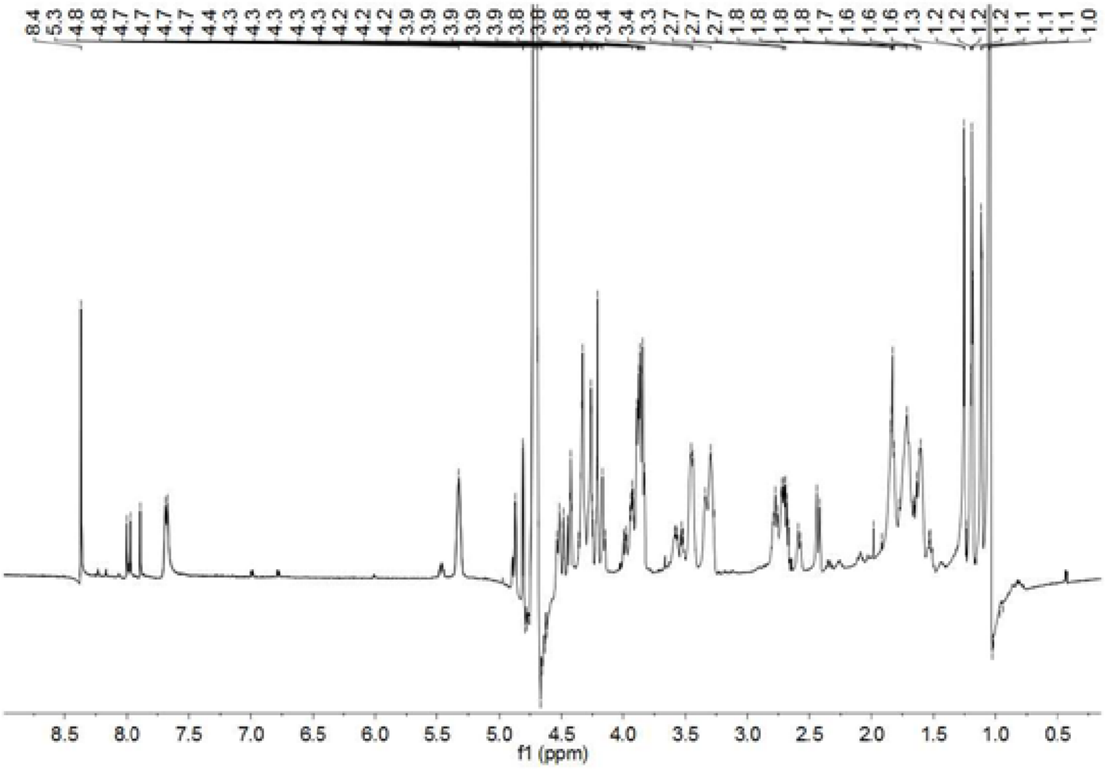

b

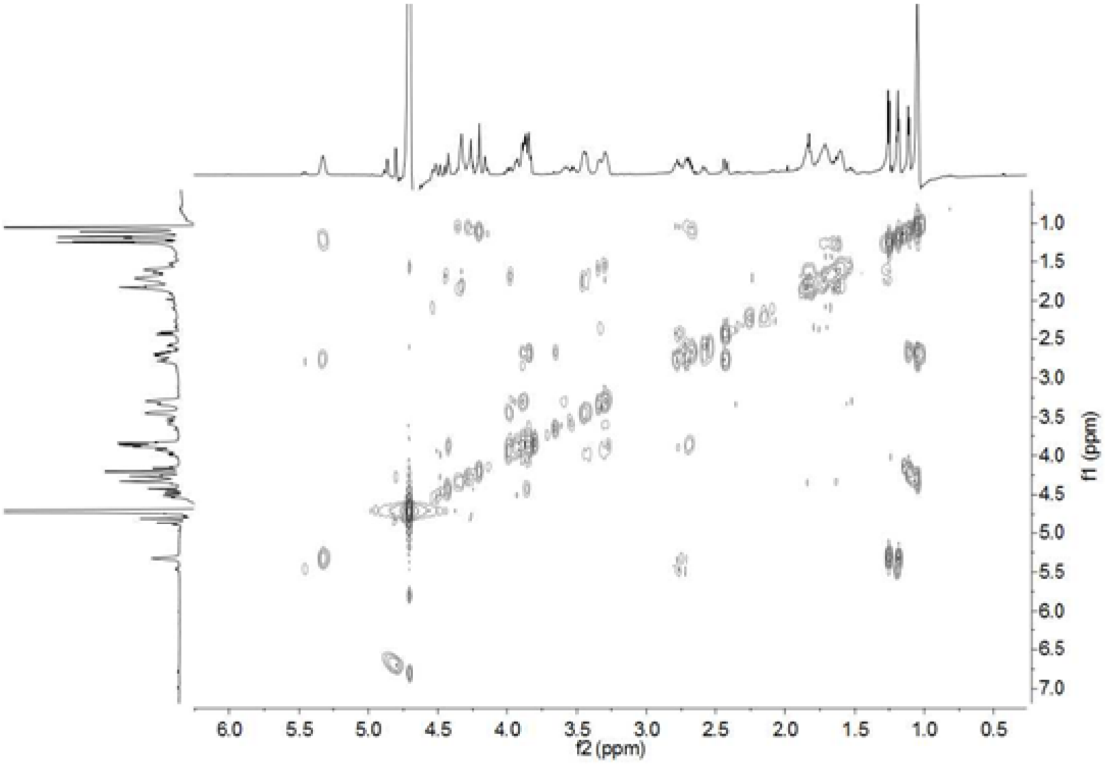

c

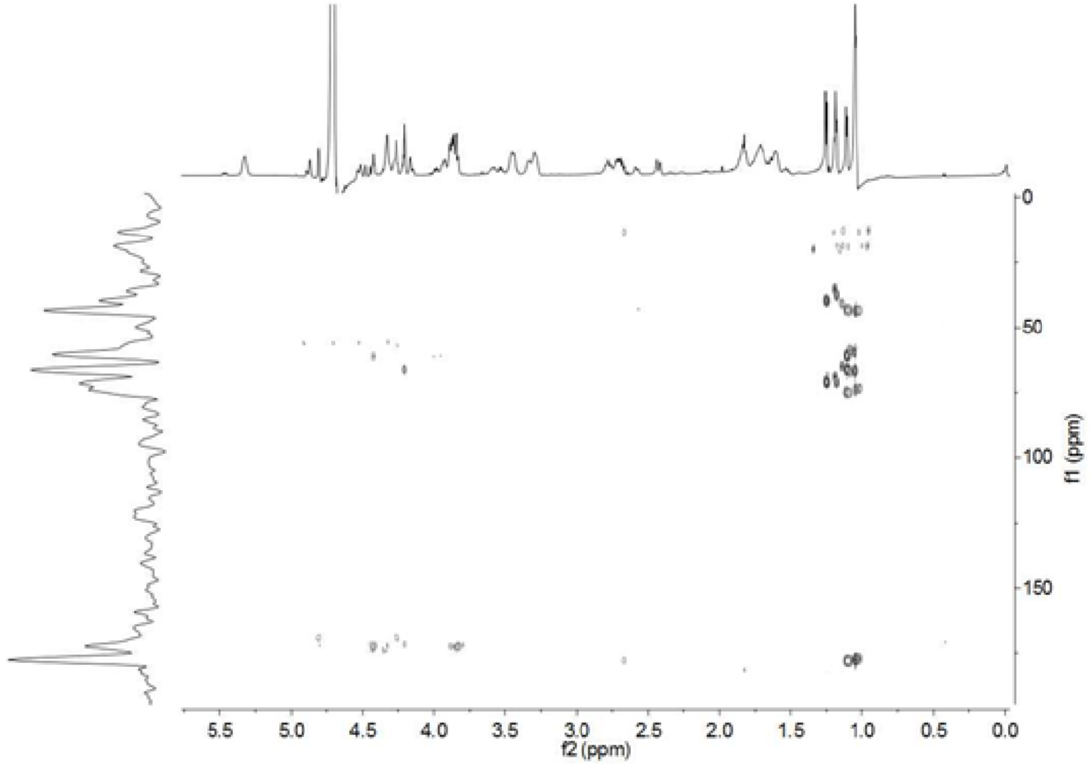

d

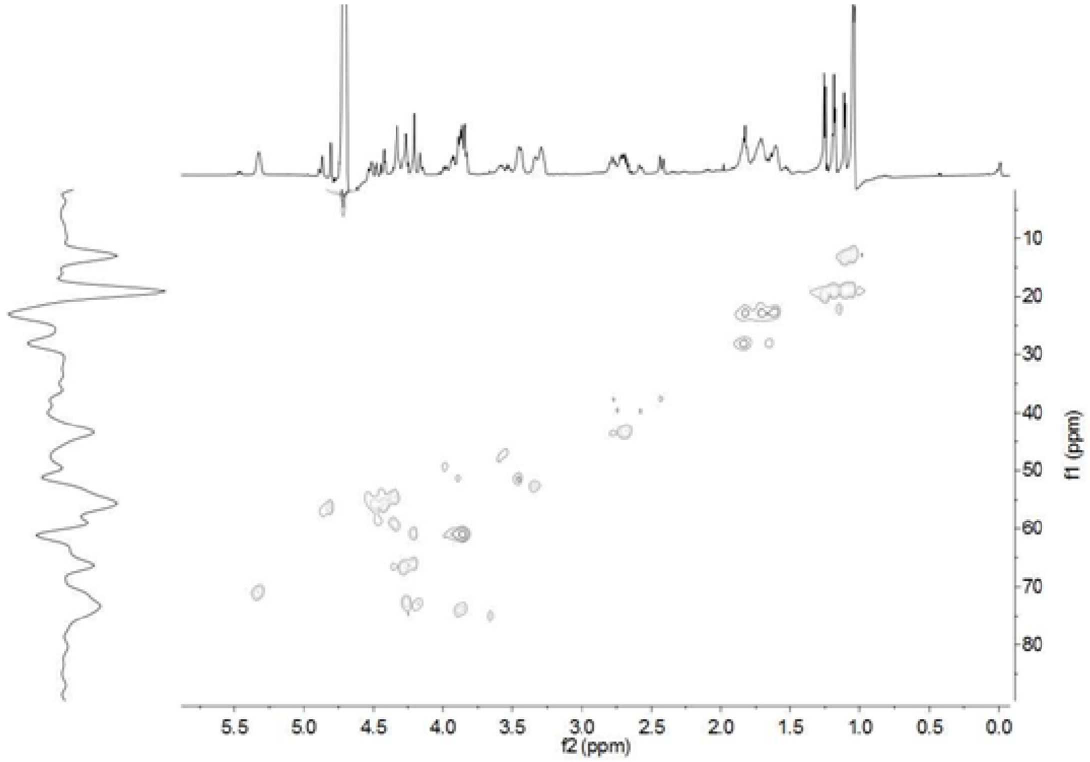

Supplementary Figure 13 NMR analysis of vacidobactin A (**4**) in D_2O. (**a**) ^{1}H NMR spectrum. (b) ^{1}H-^{1}H COSY spectrum. (**c**) ^{1}H-^{13}C HMBC spectrum. (**d**) ^{1}H-^{13}C HMQC spectrum.

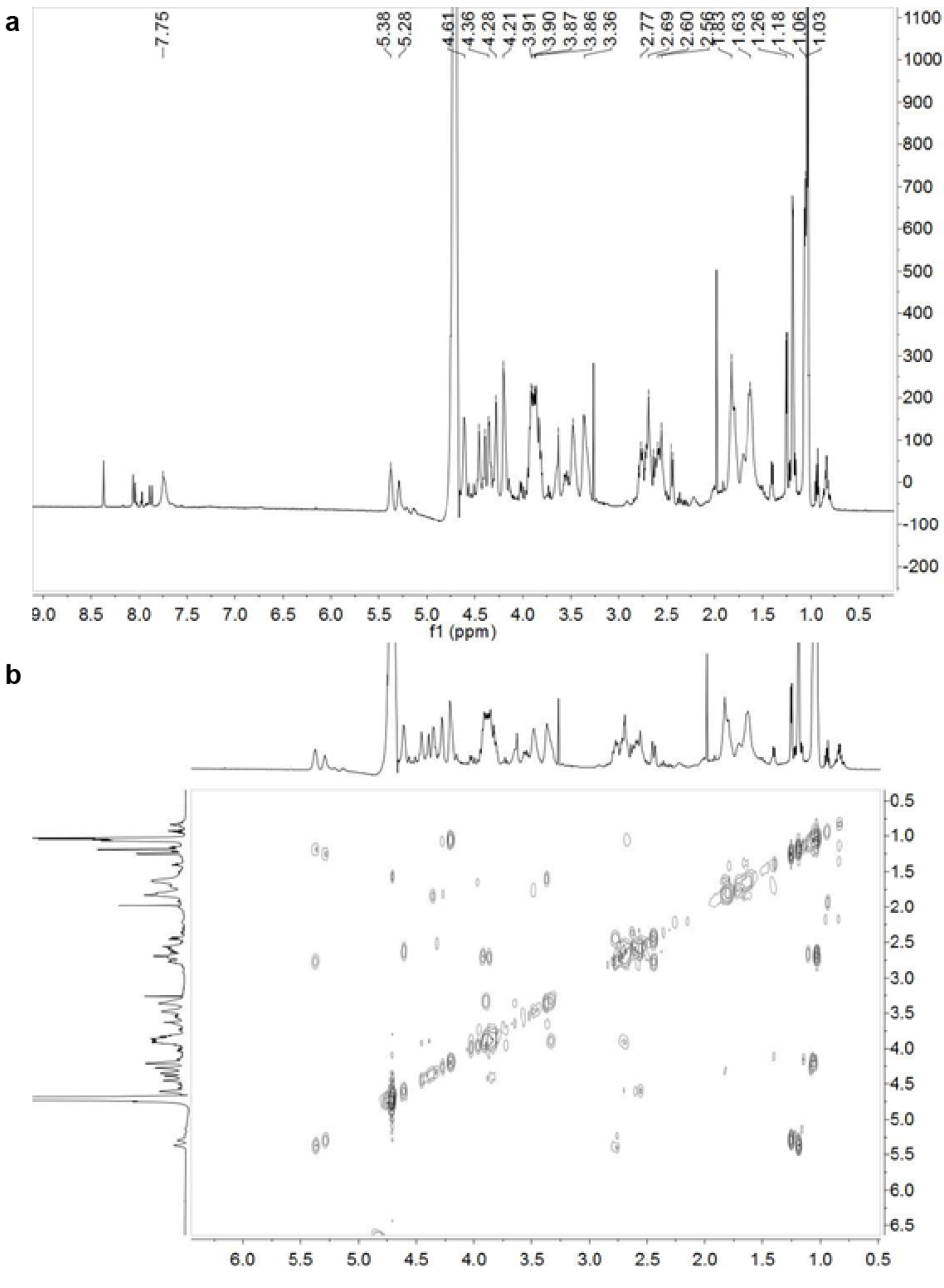
a
7.75
5.38
5.28
4.61
4.36
4.28
4.21
3.91
3.90
3.87
3.86
3.36
2.77
2.69
2.60
2.56
1.83
1.63
1.26
1.18
1.06
1.03
f1 (ppm)
b

c

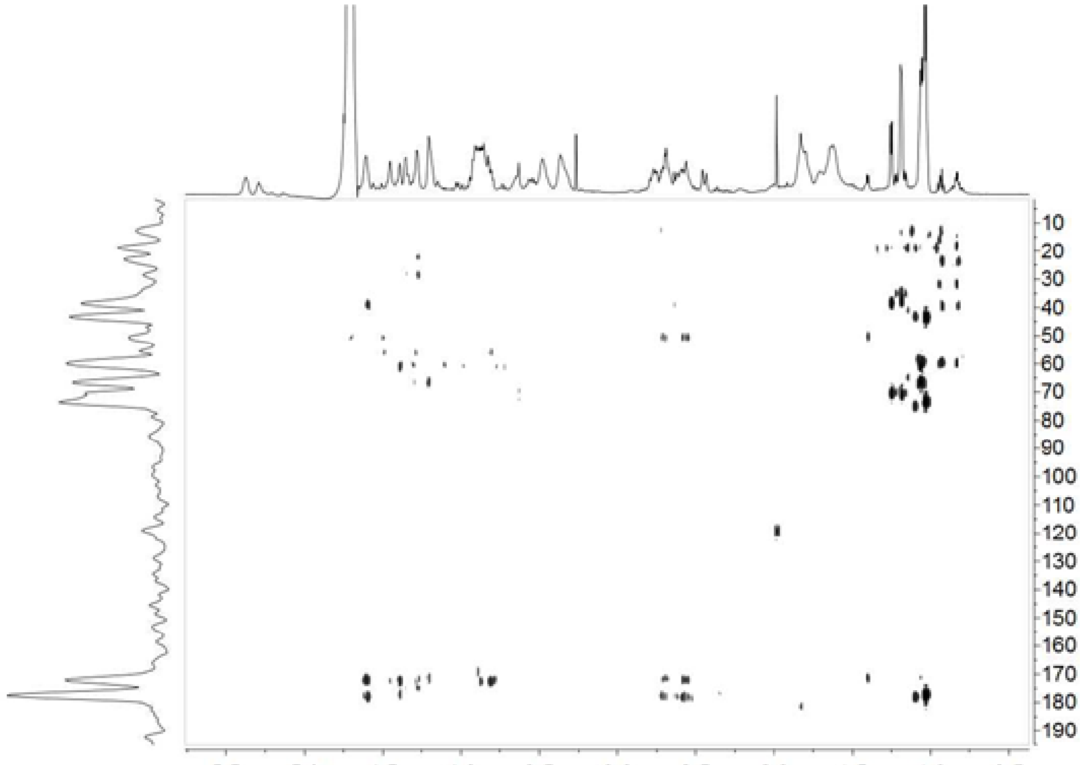

d

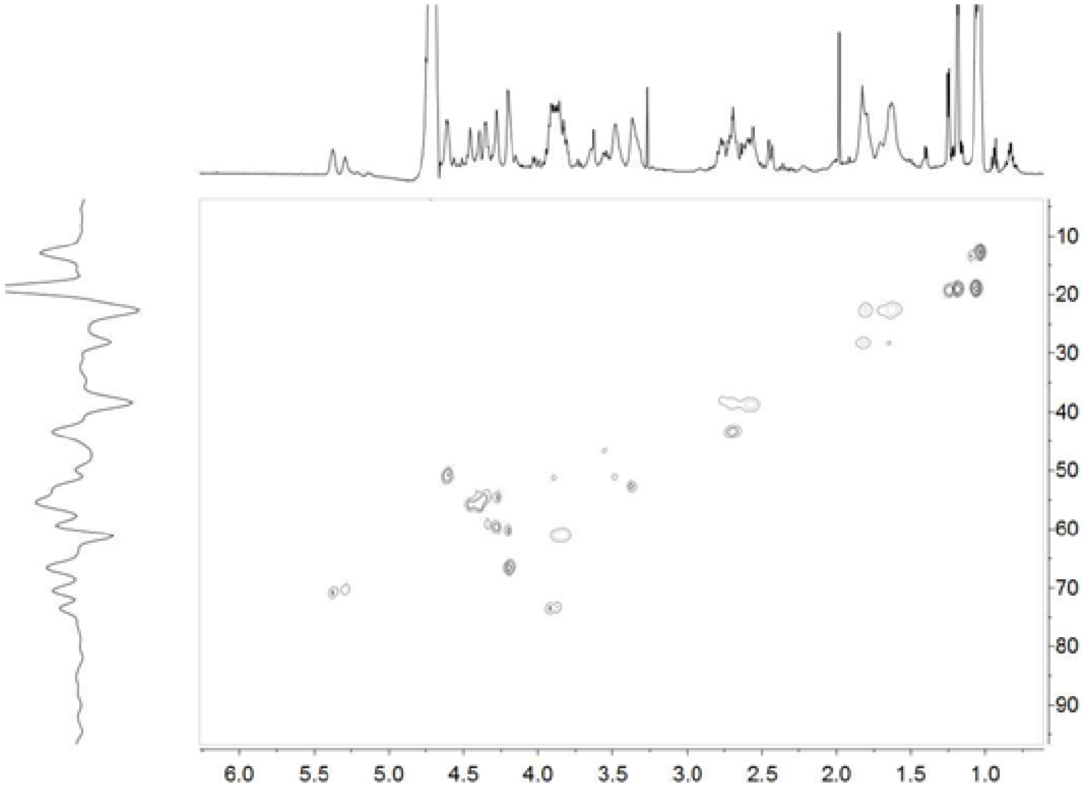

Supplementary Figure 14 NMR analysis of vacidobactin B (**5**) in D_2O. (**a**) ^{1}H NMR spectrum. (**b**) ^{1}H-^{1}H COSY spectrum. (**c**) ^{1}H-^{13}C HMBC spectrum. (**d**) ^{1}H-^{13}C HMQC spectrum.

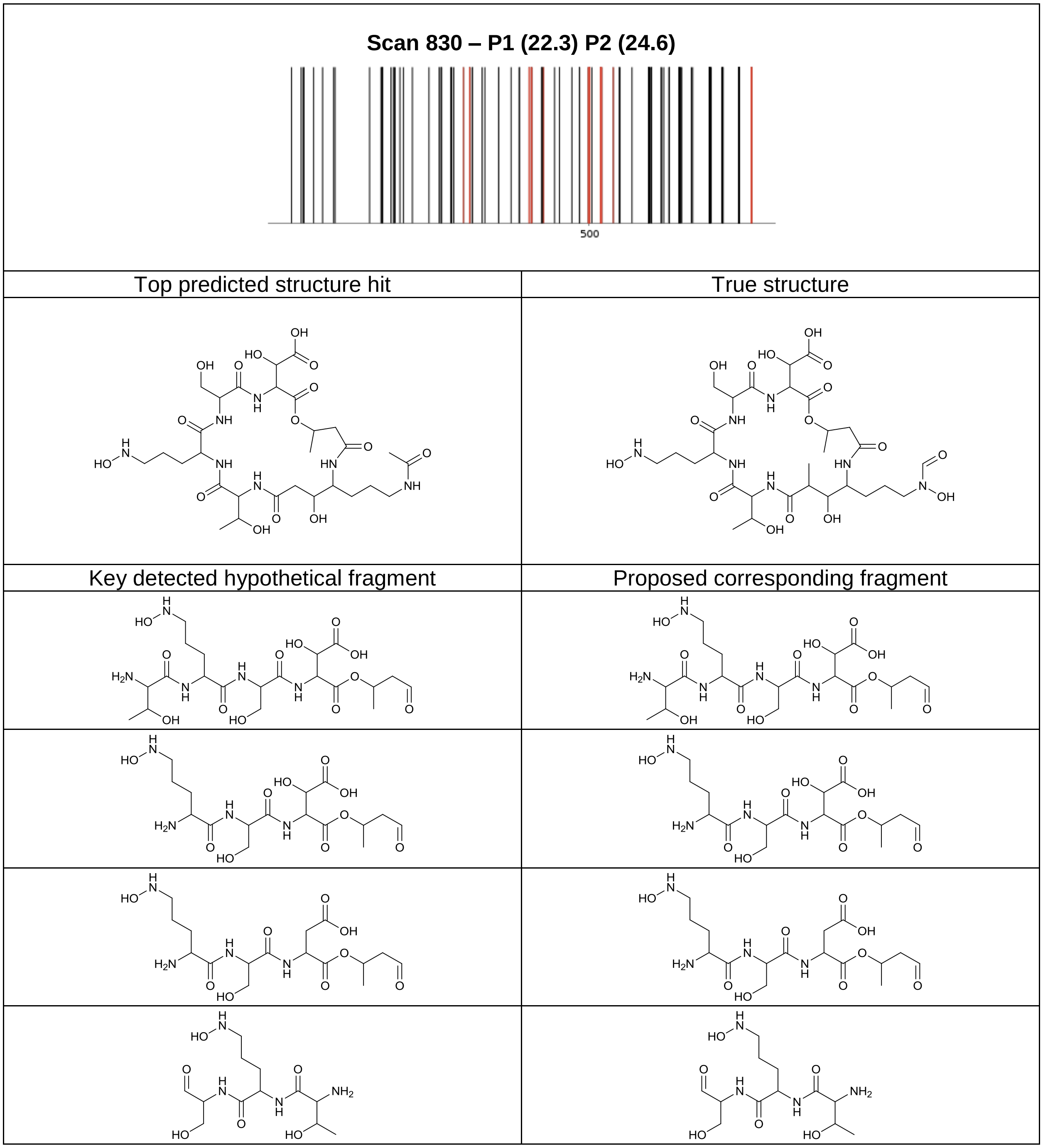

Supplementary Figure 15 Sample matched fragments between a predicted structure hit and vacidobactin A. Several matched fragments between the hypothetical compound hit and the corresponding true structure are shown. Fragments of vacidobactin A were generated using the GNP Fragmenter application. Detected hypothetical spectral fragments are shown as lines in the diagram above, with detected masses shown in red. P-scores for the scan are listed above. Full results tables for this scan and experimental run are available at http://magarveylab.ca/data/gnp.

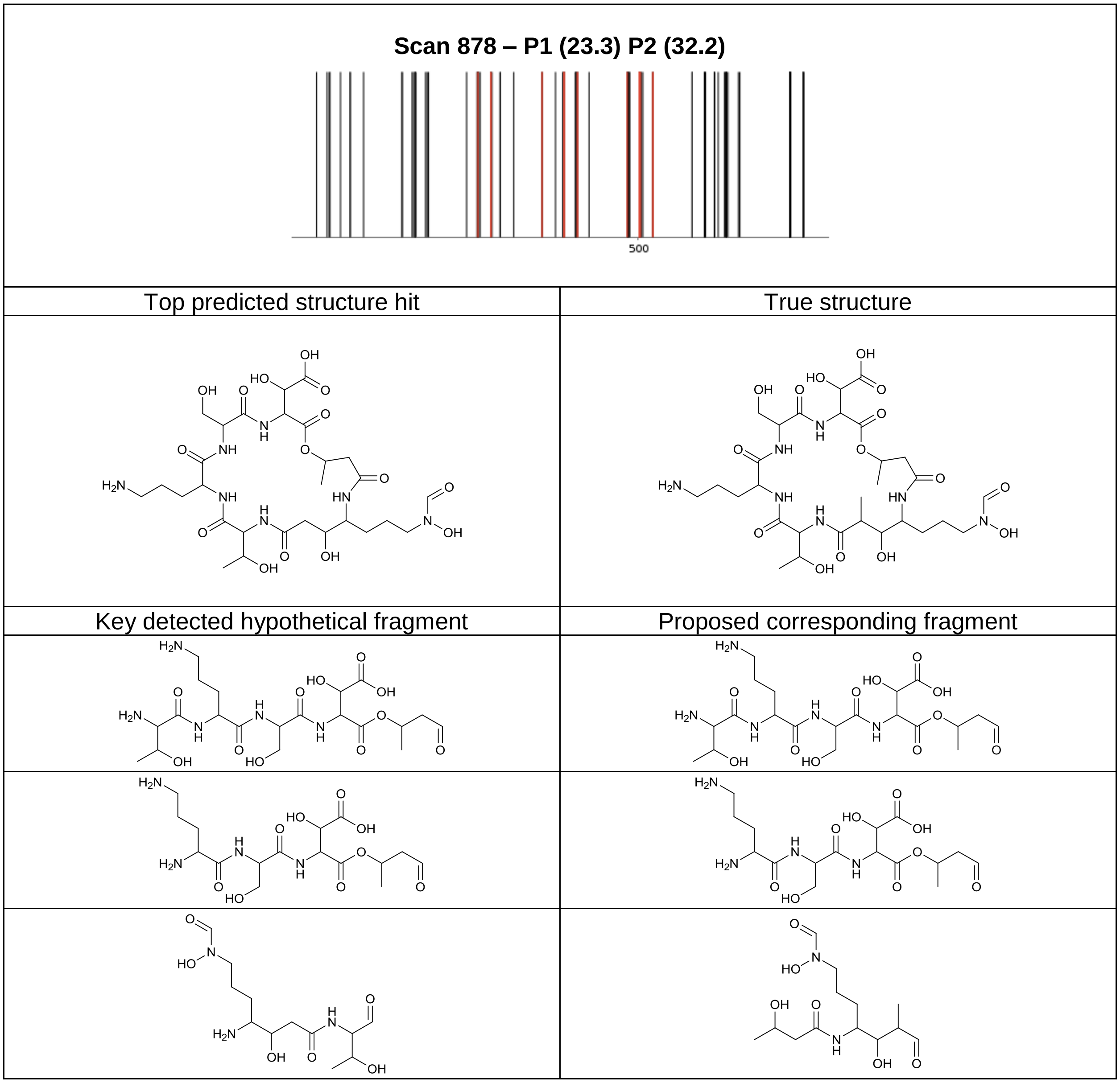

Supplementary Figure 16 Sample matched fragments between a predicted structure hit and vacidobactin B. Several matched fragments between the hypothetical compound hit and the corresponding true structure are shown. Fragments of vacidobactin B were generated using the GNP Fragmenter application. Detected hypothetical spectral fragments are shown as lines in the diagram above, with detected masses shown in red. P-scores for the scan are listed above. Full results tables for this scan and experimental run are available at http://magarveylab.ca/data/gnp.

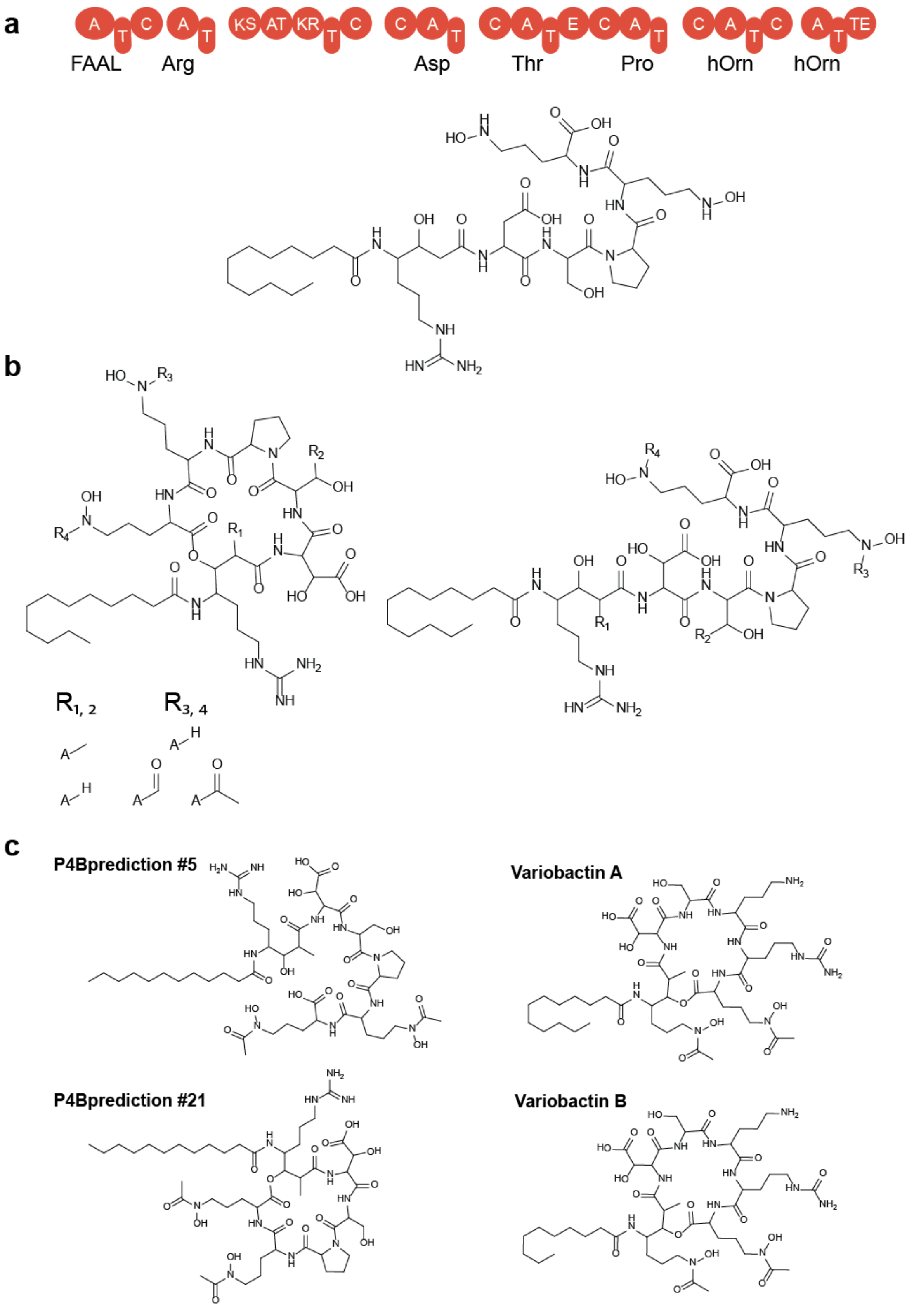

Supplementary Figure 17 Prediction, combinatorialization, and detection of variobactin metabolites. (**a**) GNP detected NRPS biosynthetic gene cluster from *Variovorax paradoxus* P4B, with predicted monomers listed and GNP-generated prediction shown. (**b**) Structures used for combinatorialization. (**c**) Structure of the most frequent hits detected within the *V. paradoxus* P4B LC-MS/MS data (*left*), as well as the true associated metabolites, variobactin A and B (*right*).

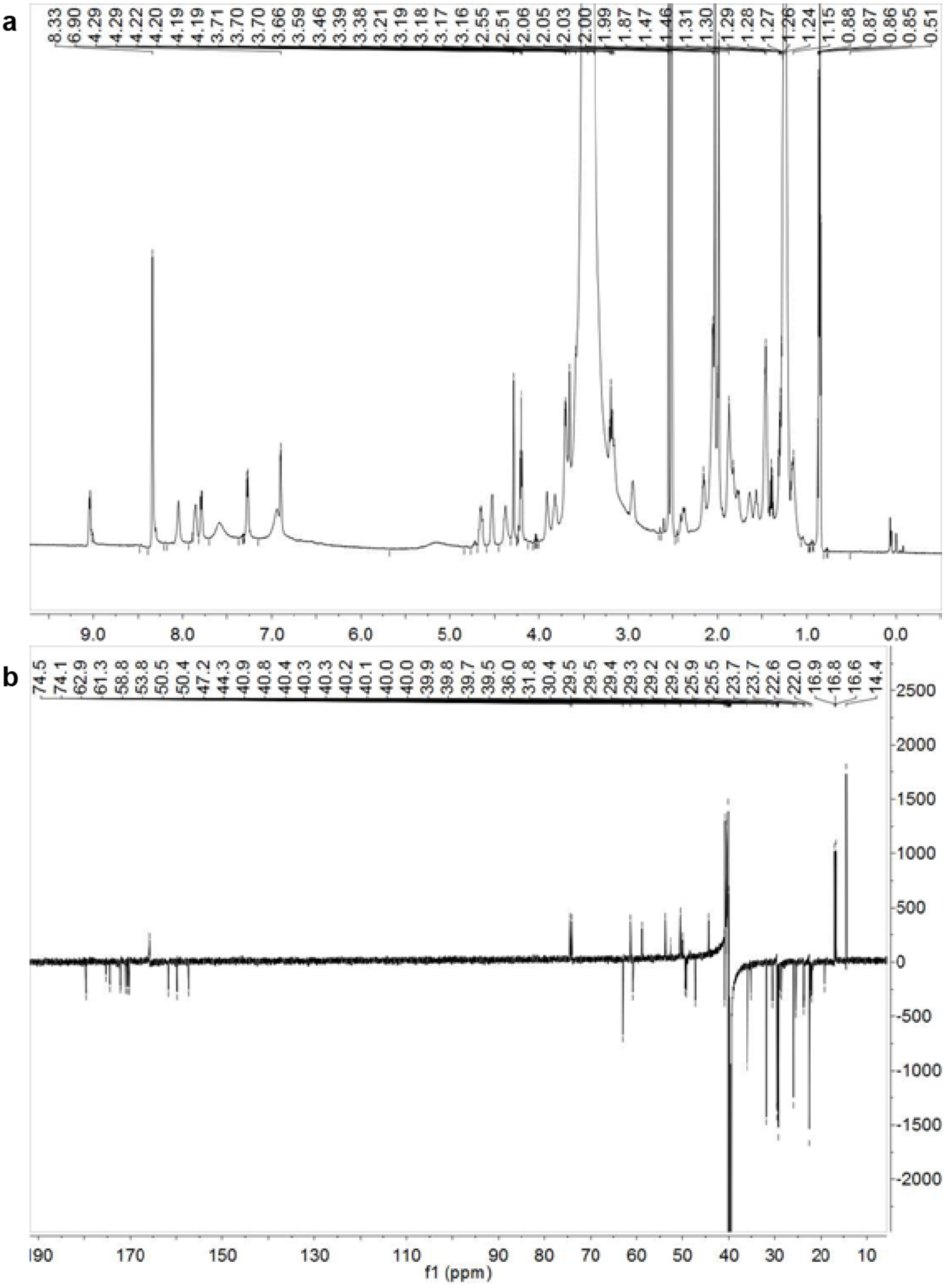
a
8.33 6.90 4.29 4.29 4.22 4.20 4.19 4.19 3.71 3.70 3.70 3.66 3.59 3.46 3.39 3.38 3.21 3.19 3.18 3.17 3.16 2.55 2.51 2.06 2.05 2.03 2.00 1.99 1.87 1.47 1.46 1.31 1.30 1.29 1.28 1.27 1.26 1.24 1.15 0.88 0.87 0.86 0.85 0.51
9.0 8.0 7.0 6.0 5.0 4.0 3.0 2.0 1.0 0.0
b
74.5 74.1 62.9 61.3 58.8 53.8 50.5 50.4 47.2 44.3 40.9 40.8 40.4 40.3 40.3 40.2 40.1 40.0 40.0 39.9 39.8 39.7 39.5 36.0 31.8 30.4 29.5 29.5 29.4 29.3 29.2 29.2 25.9 25.5 23.7 23.7 22.6 22.0 16.9 16.8 16.6 14.4
2500 2000 1500 1000 500 0 -500 -1000 -1500 -2000
190 170 150 130 110 90 80 70 60 50 40 30 20 10
f1 (ppm)

c

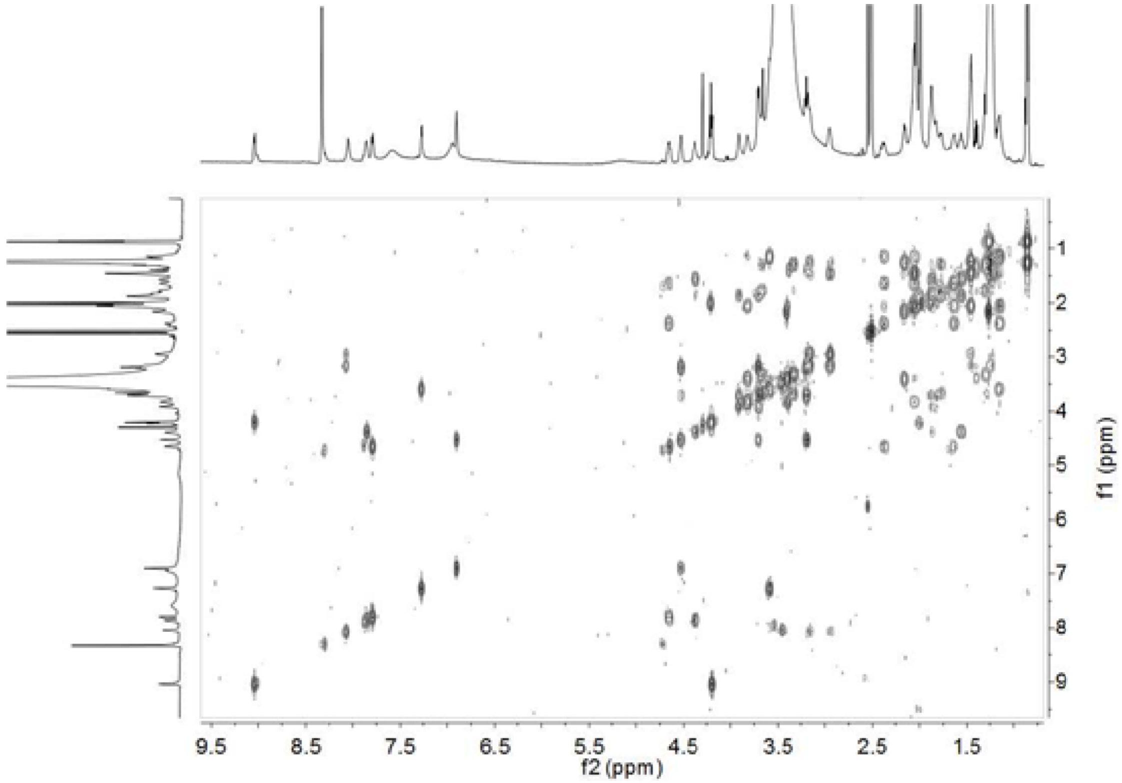

9.5 8.5 7.5 6.5 5.5 4.5 3.5 2.5 1.5
f2 (ppm)
1 2 3 4 5 6 7 8 9
f1 (ppm)

d

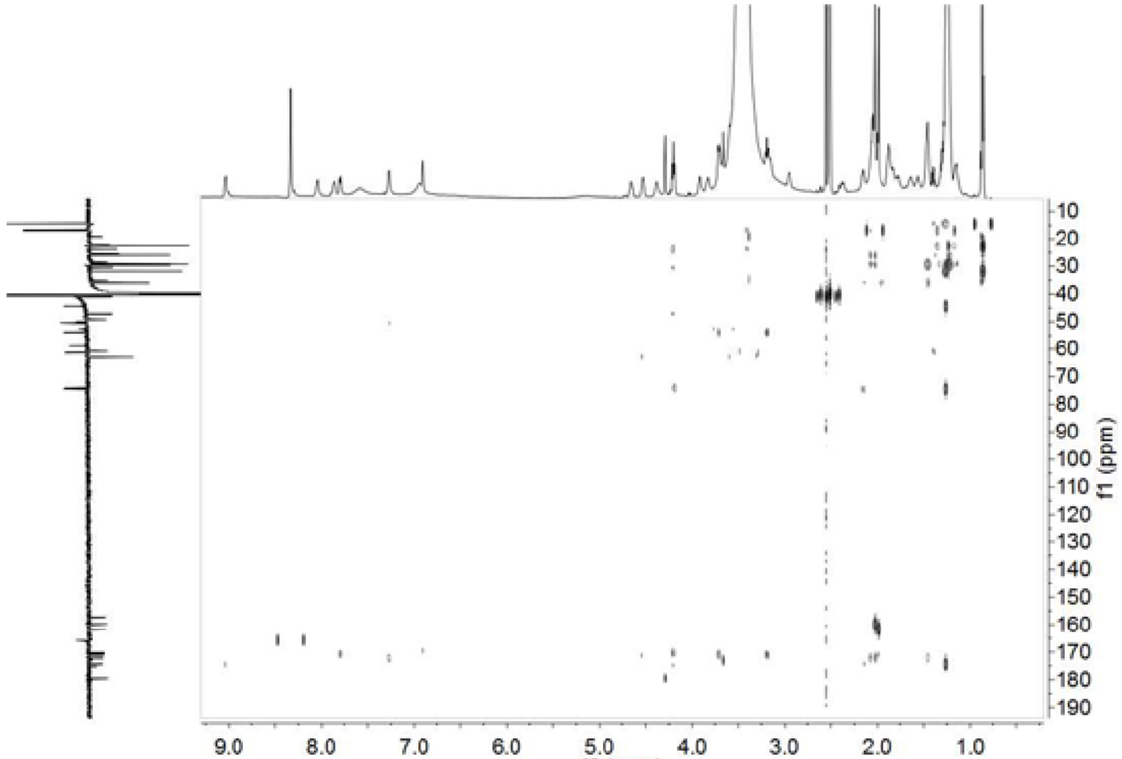

9.0 8.0 7.0 6.0 5.0 4.0 3.0 2.0 1.0
f2 (ppm)
10 20 30 40 50 60 70 80 90 100 110 120 130 140 150 160 170 180 190
f1 (ppm)

e

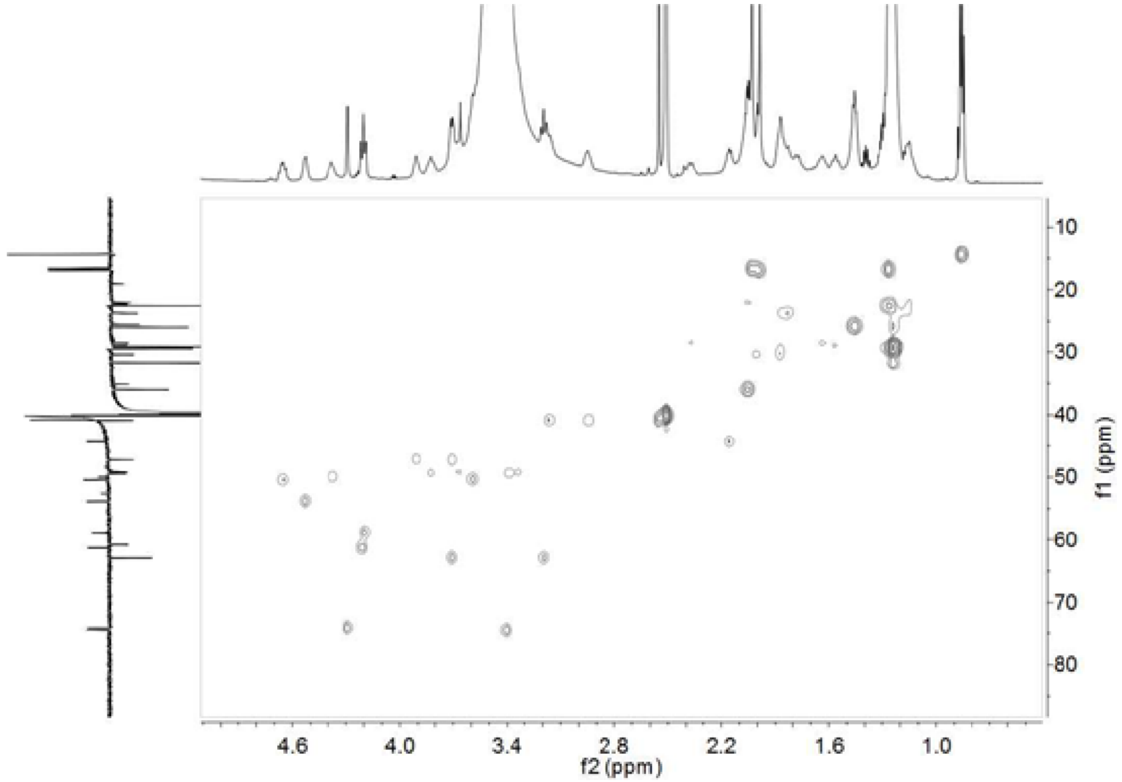

Supplementary Figure 18 NMR analysis of variobactin A (**6**) in DMSO. (**a**) ^{1}H NMR spectrum. (**b**) ^{13}C DEPTq spectrum. (**c**) ^{1}H-^{1}H COSY spectrum. (**d**) ^{1}H-^{13}C HMBC spectrum. (**e**) ^{1}H-^{13}C HMQC spectrum.

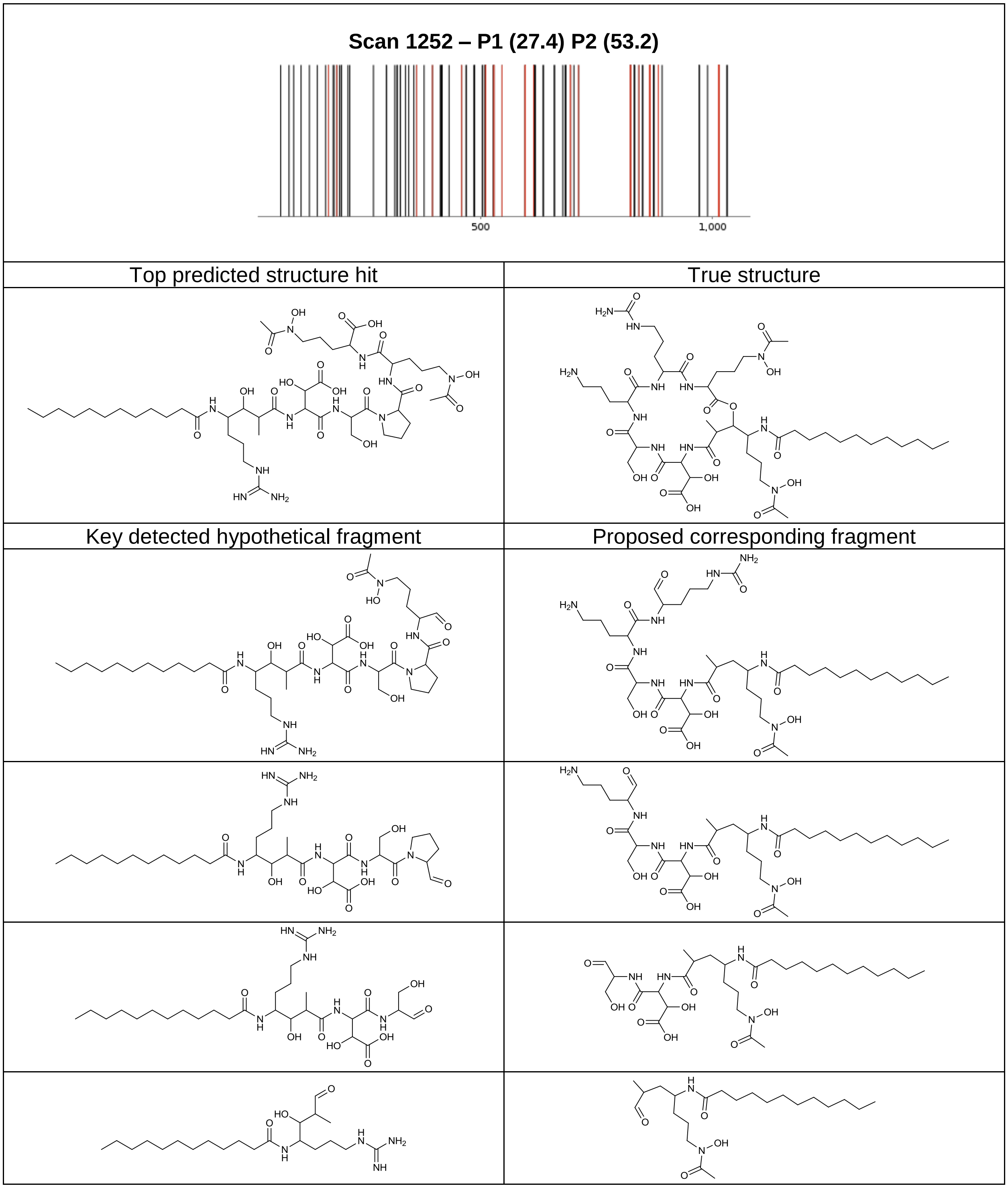

Supplementary Figure 19 Sample matched fragments between a predicted structure hit and variobactin A. Several matched fragments between the hypothetical compound hit and the corresponding true structure are shown. Fragments of variobactin A were generated using the GNP Fragmenter application. Detected hypothetical spectral fragments are shown as lines in the diagram above, with detected masses shown in red. P-scores for the scan are listed above. Full results tables for this scan and experimental run are available at http://magarveylab.ca/data/gnp.

Scan 1107 – P1 (18.4) P2 (21.3)

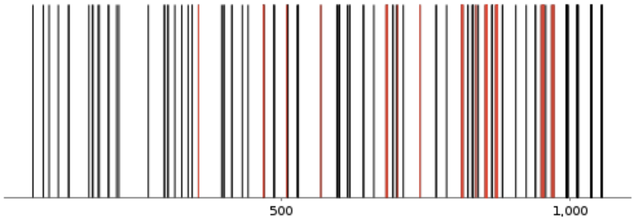

Top predicted structure hit	True structure
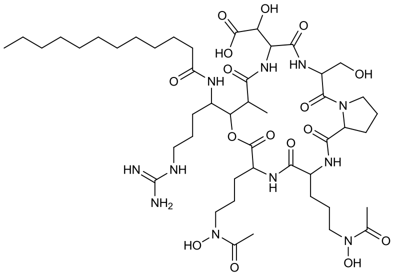	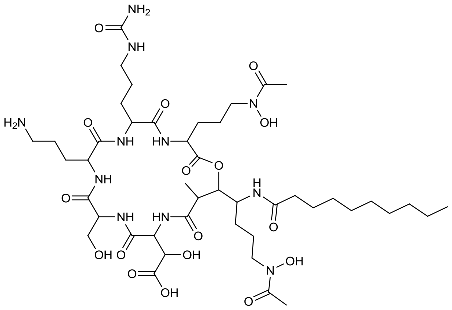
**Key detected hypothetical fragment**	**Proposed corresponding fragment**
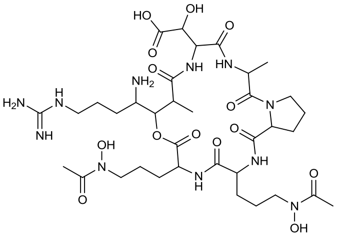	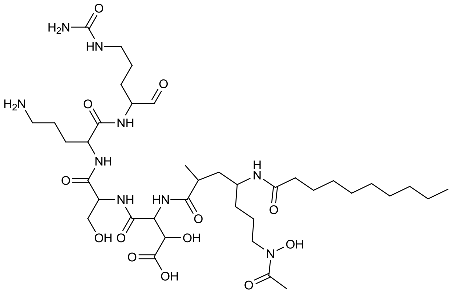
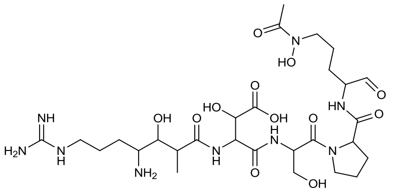	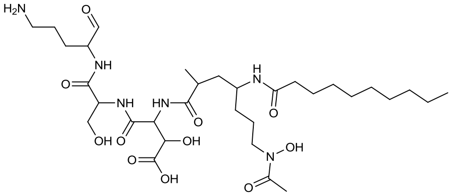
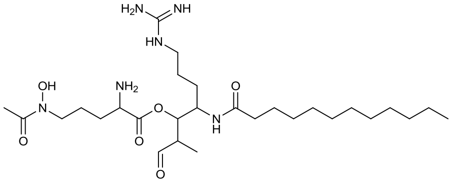	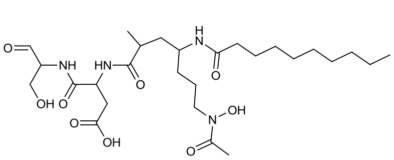
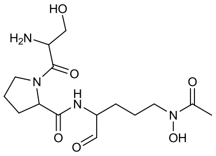	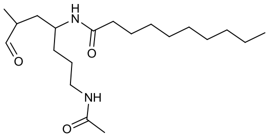

Supplementary Figure 20 Sample matched fragments between a predicted structure hit and variobactin B. Several matched fragments between the hypothetical compound hit and the corresponding true structure are shown. Fragments of variobactin B were generated using the GNP Fragmenter application. Detected hypothetical spectral fragments are shown as lines in the diagram above, with detected masses shown in red. P-scores for the scan are listed above. Full results tables for this scan and experimental run are available at http://magarveylab.ca/data/gnp.

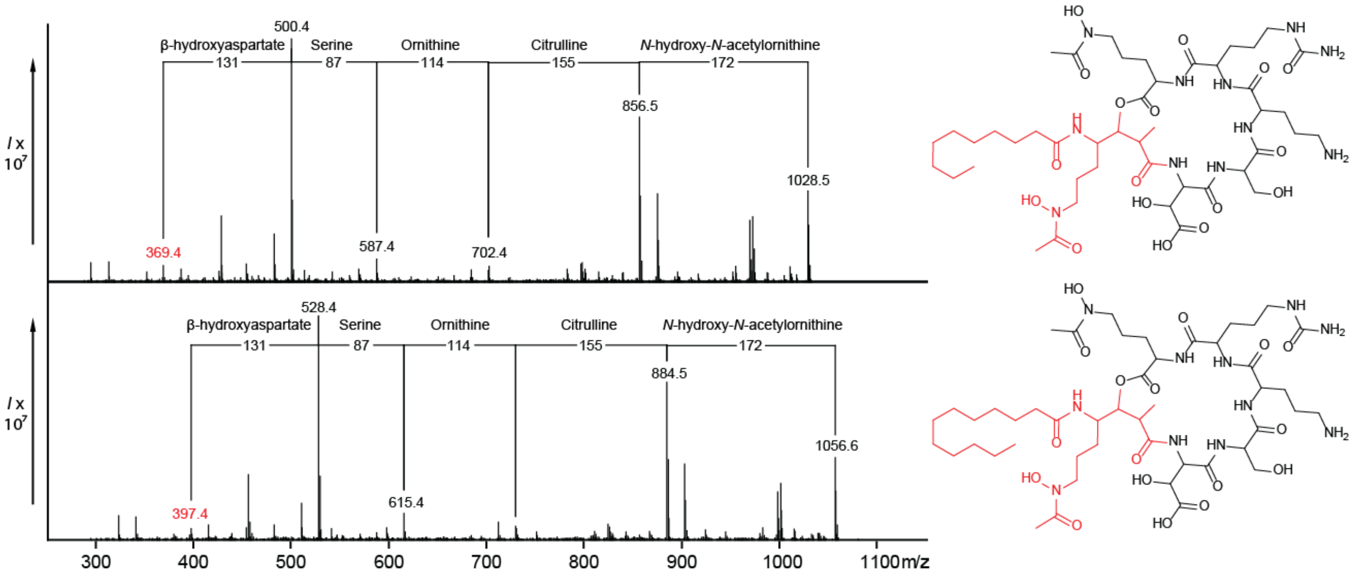

Supplementary Figure 21 MS/MS comparison of variobactin A and B. LC-MS/MS fragments of variobactin B (*top*) and A (*bottom*) indicate the presented structures, with a 28 Da difference in fragment mass attributed to the addition of two CH_2 units to the acyl tail of variobactin A.

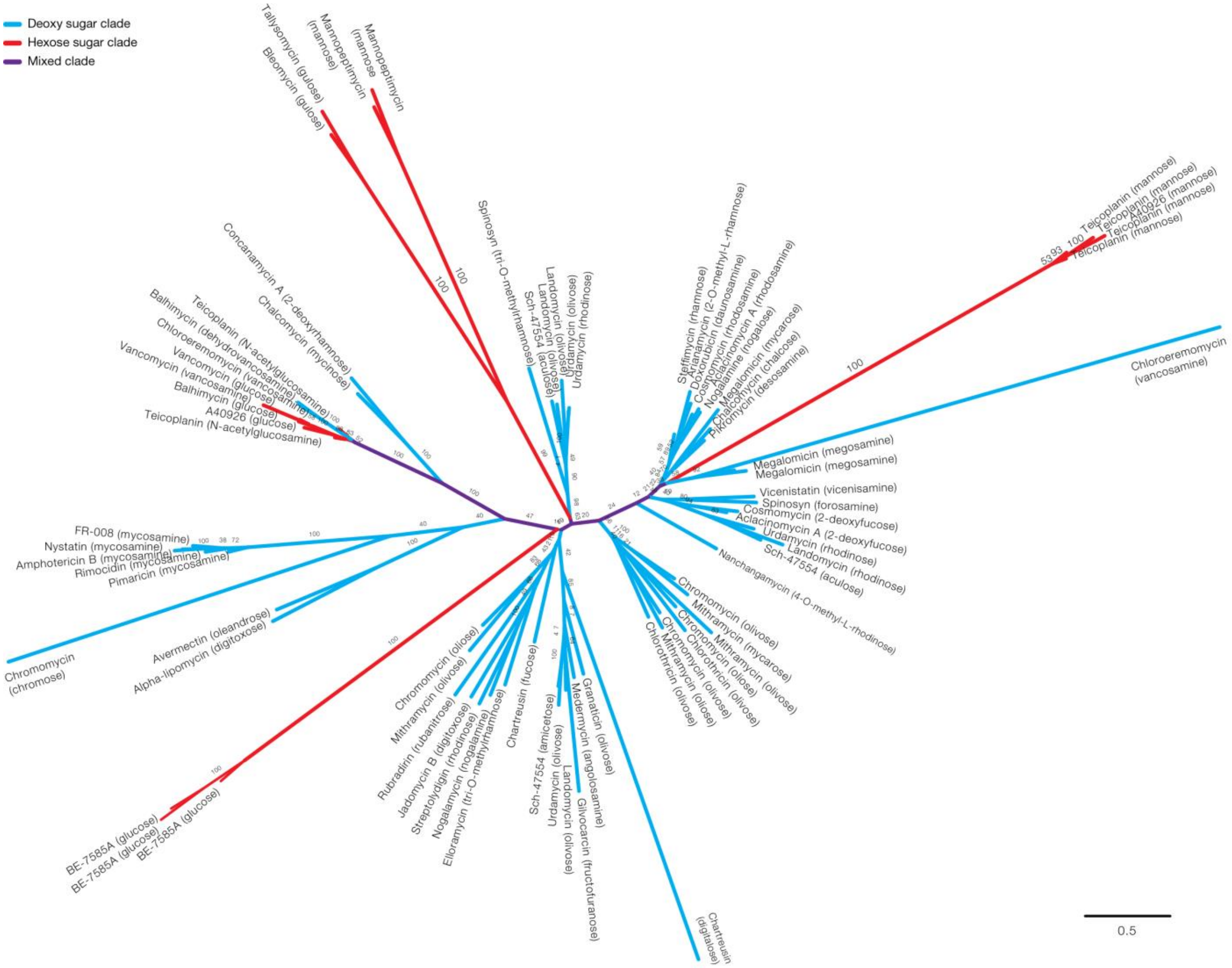

Supplementary Figure 22 Phylogenetic analysis of natural product glycosyltransferases. A manually curated database of 82 natural product glycosyltransferase domains and substrates was aligned and used to construct a phylogenetic tree. Results indicate that natural product glycosyltransferases specific to hexose sugars form distinct clades, including glycopeptide mannosyltransferases, BE-7585A-type glucosyltransferases, and a fouth mixed mixed clade, consisting of both hexose and deoxysugar glycosyltransferases, consisting of position-specific glycopeptide glycosyltransferases.

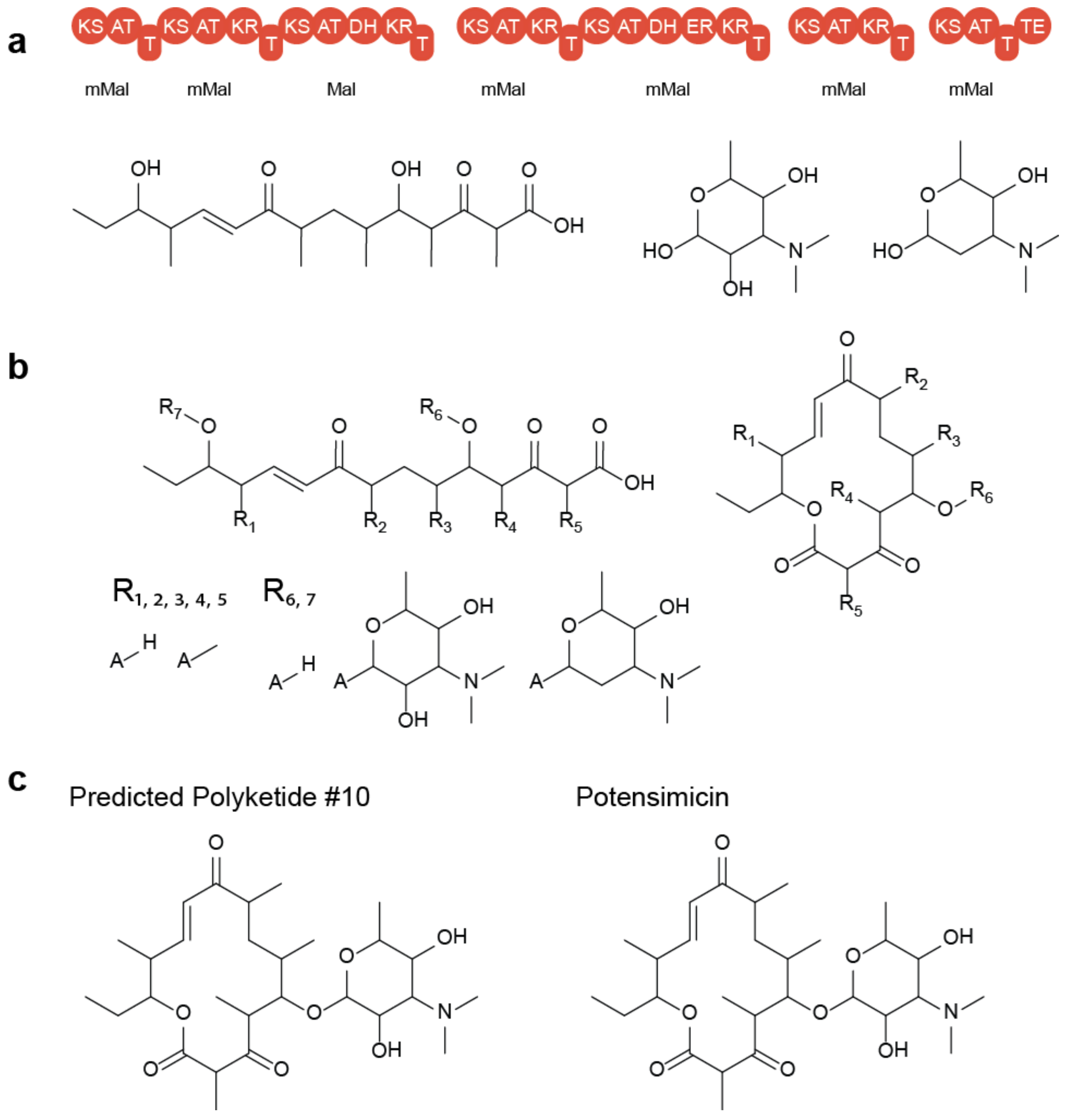

Supplementary Figure 23 Prediction, combinatorialization, and detection of potensimicin. (**a**) GNP detected PKS biosynthetic gene cluster from *Nocardiopsis potens*, with predicted monomers listed and GNP-generated prediction shown. (**b**) Structures used for combinatorialization. (**c**) Structure of the most frequent hit detected within the *N. potens* LC-MS/MS data (*left*), as well as the true associated metabolite, potensimicin (*right*).

a

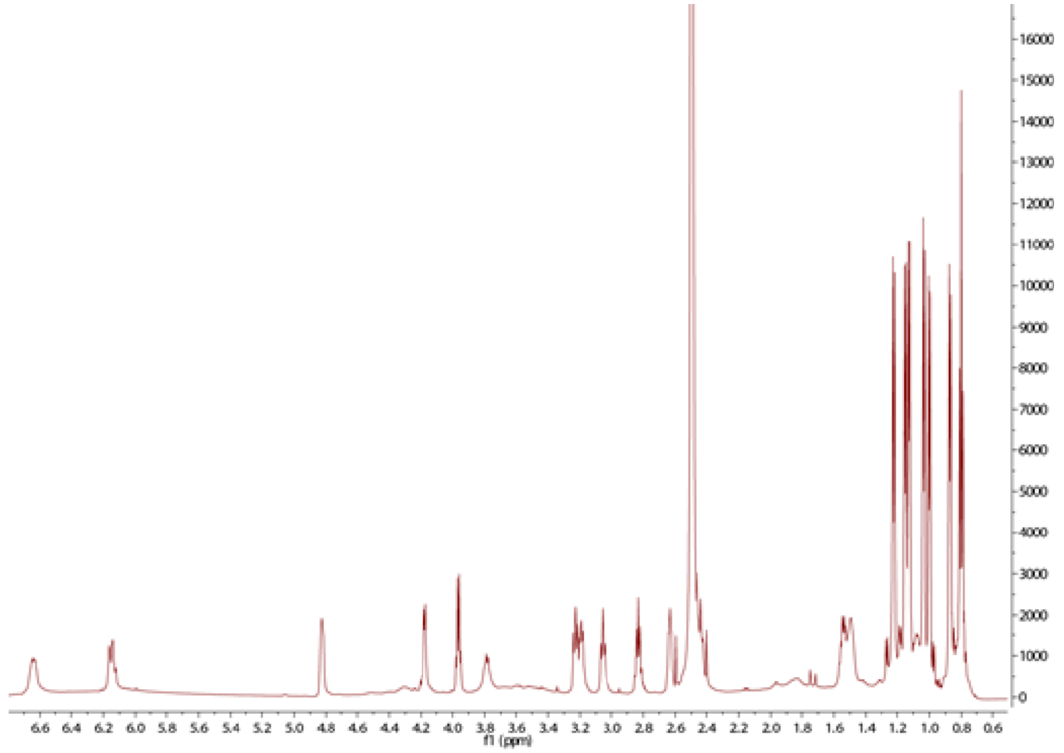

b

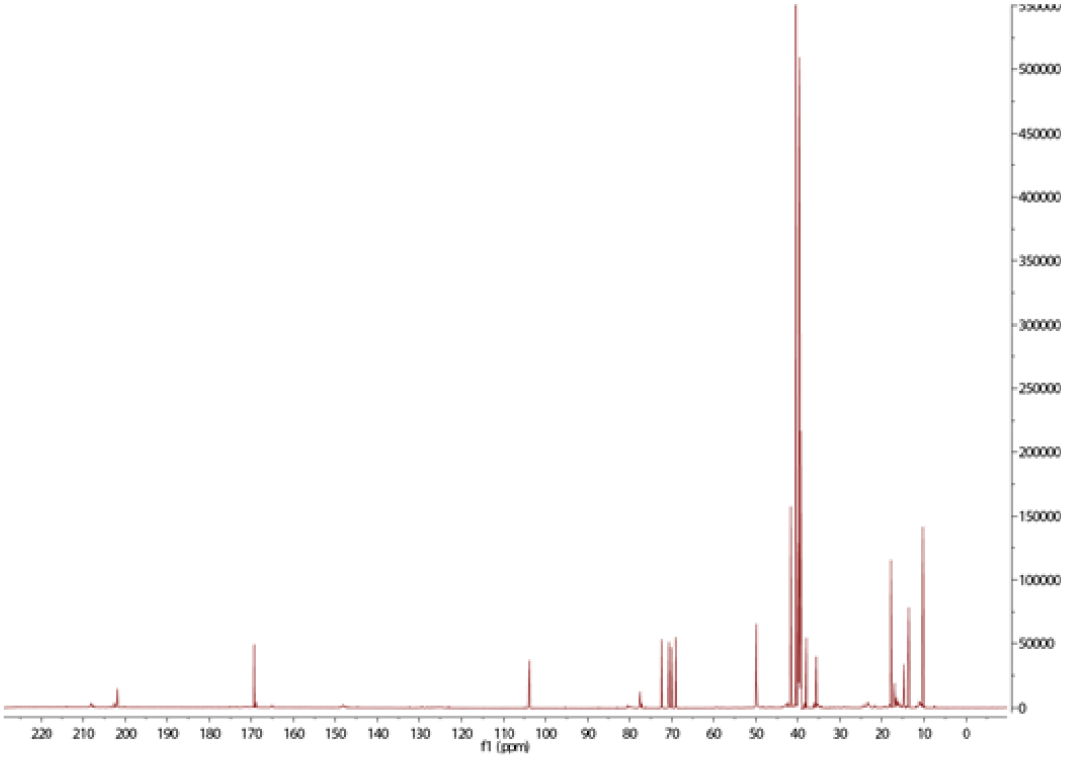

Supplementary Figure 24 NMR analysis of potensimicin (**7**) in DMSO. (**a**) ^{1}H NMR spectrum. (**b**) ^{13}C NMR spectrum.

a

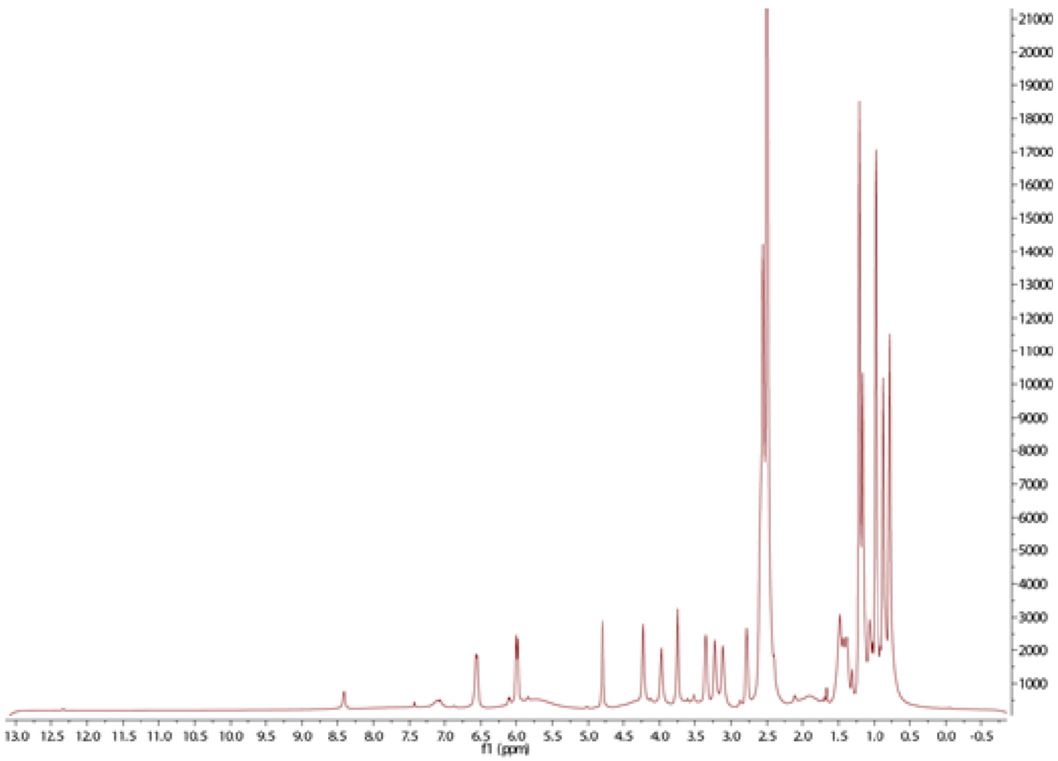

b

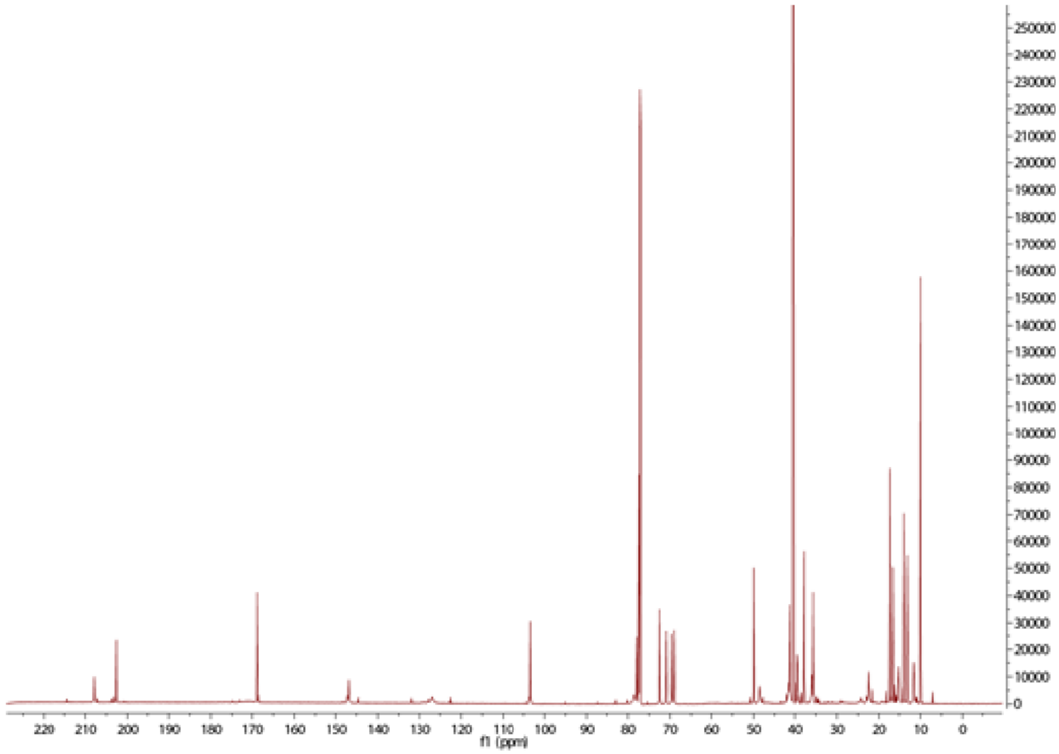

c

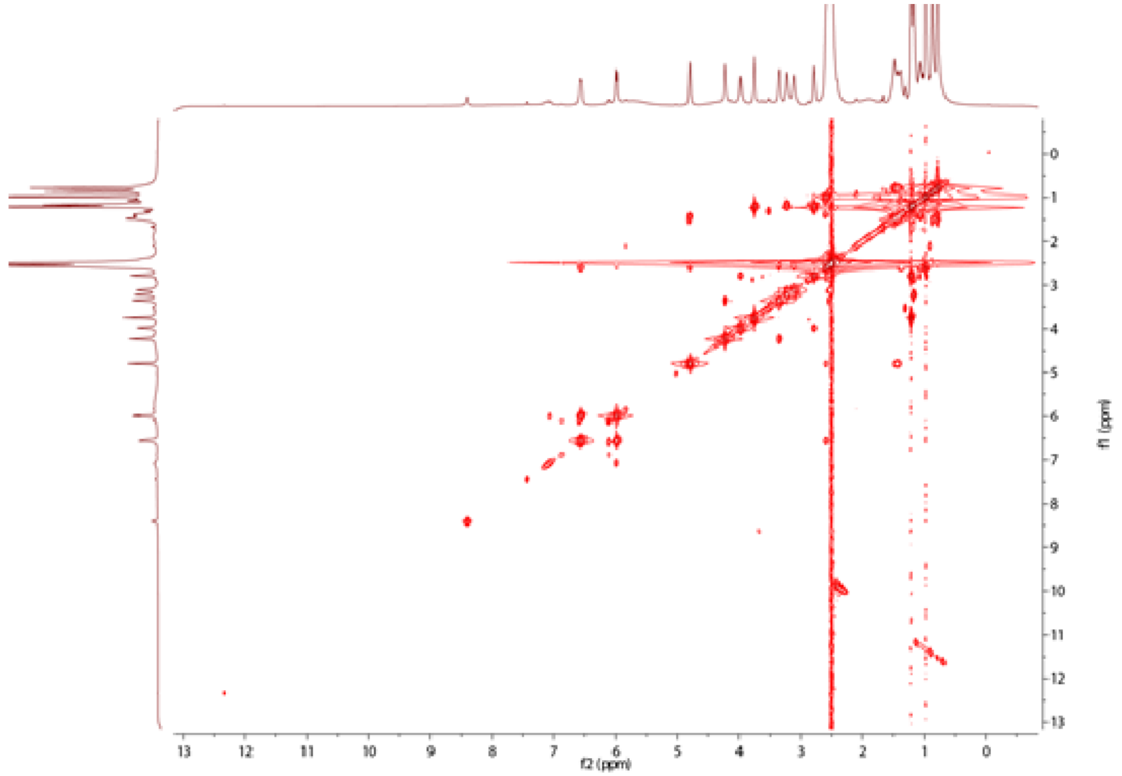

d

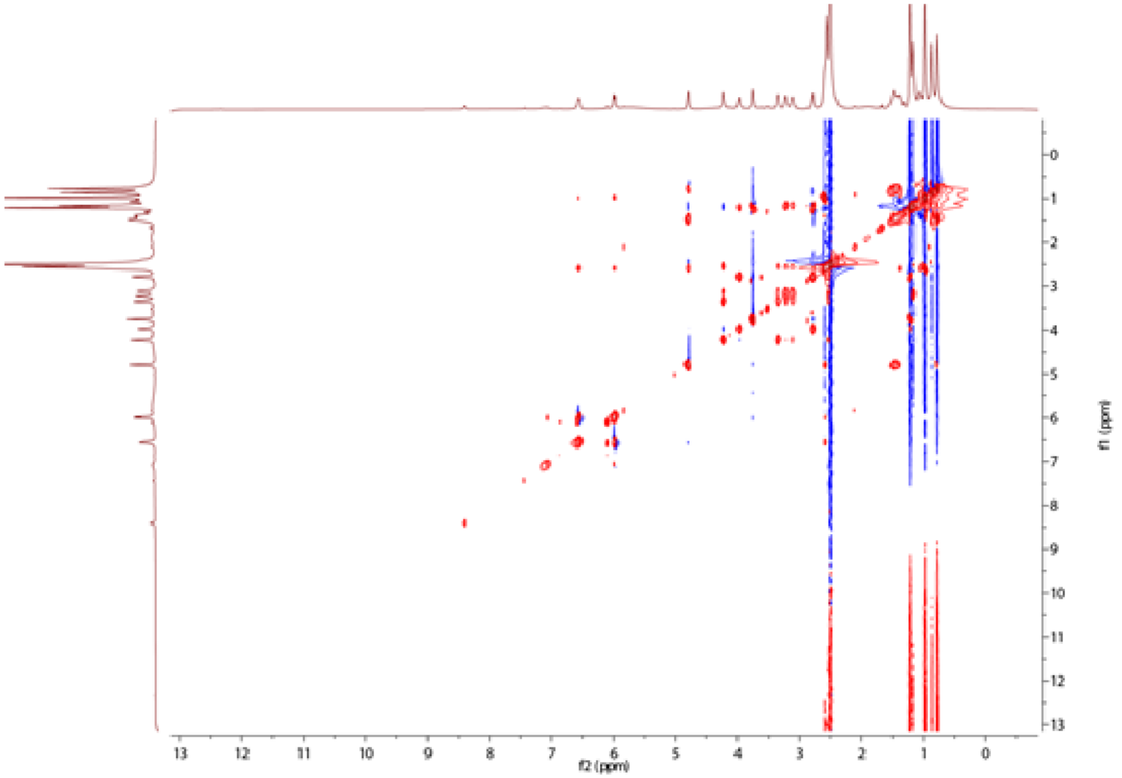

e

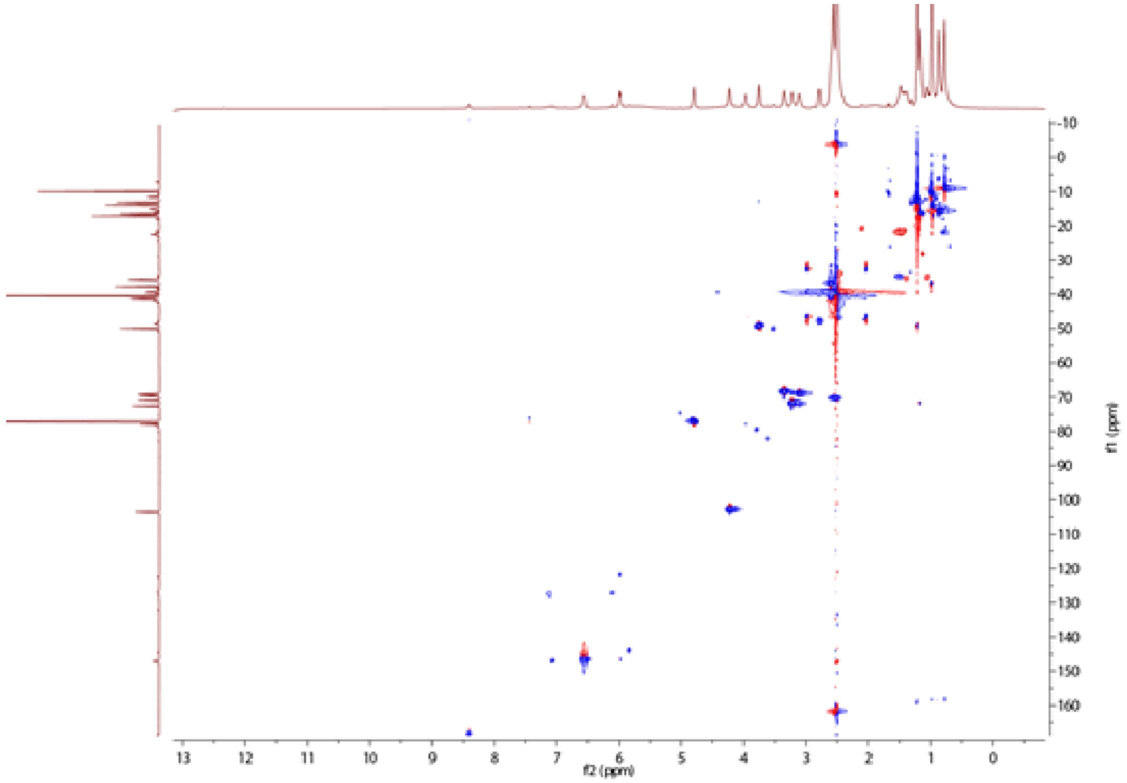

f

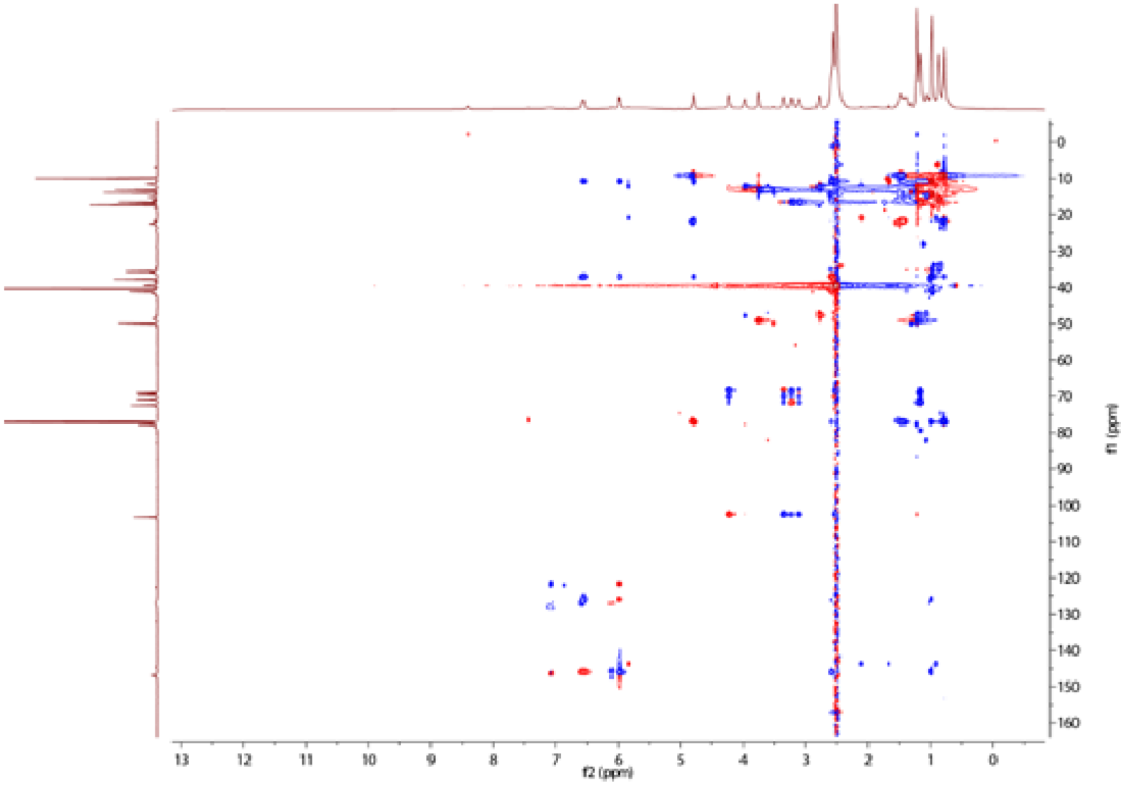

g

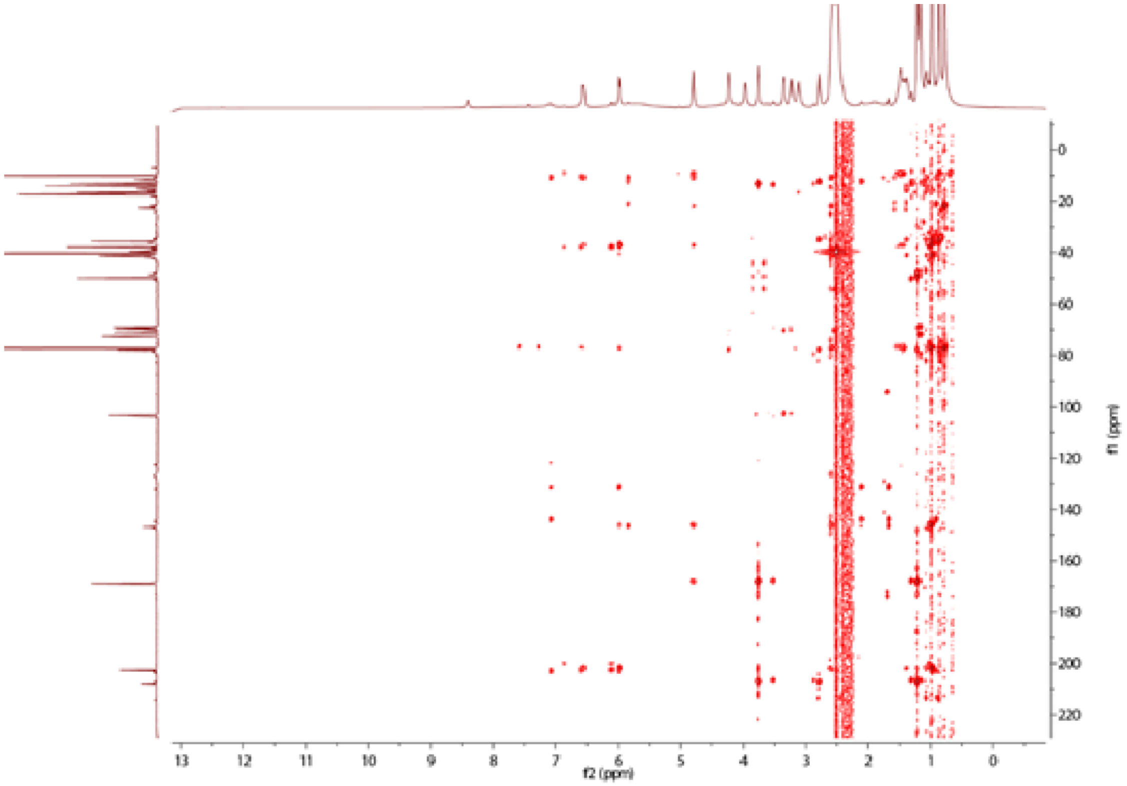

h

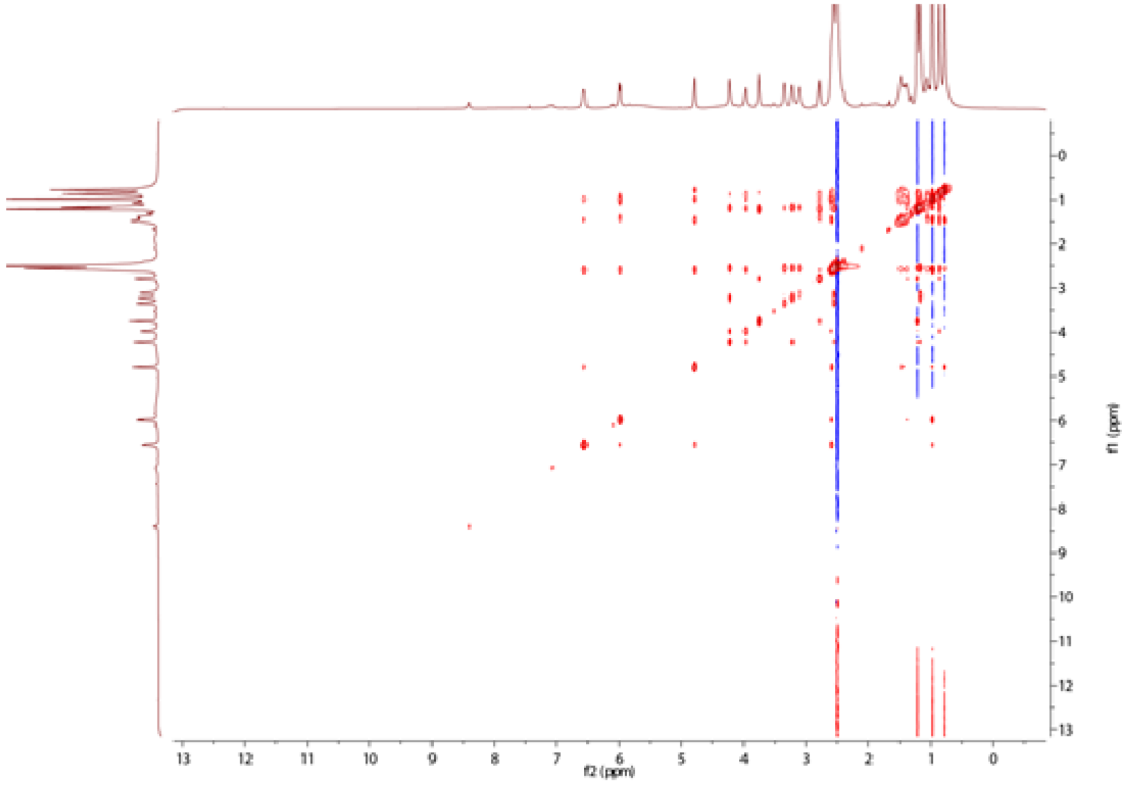

Supplementary Figure 25 NMR analysis of potensimicin (**7**) in $CDCl_3$. (**a**) ^{1}H NMR spectrum. (**b**) ^{13}C NMR spectrum. (**c**) ^{1}H-^{1}H COSY spectrum. (**d**) ^{1}H-^{1}H TOCSY spectrum. (**e**) ^{1}H-^{13}C HSQC spectrum. (**f**) ^{1}H-^{13}C HSQC-TOCSY spectrum. (**g**) ^{1}H-^{13}C HMBC spectrum. (**h**) ^{1}H-^{1}H NOESY spectrum.

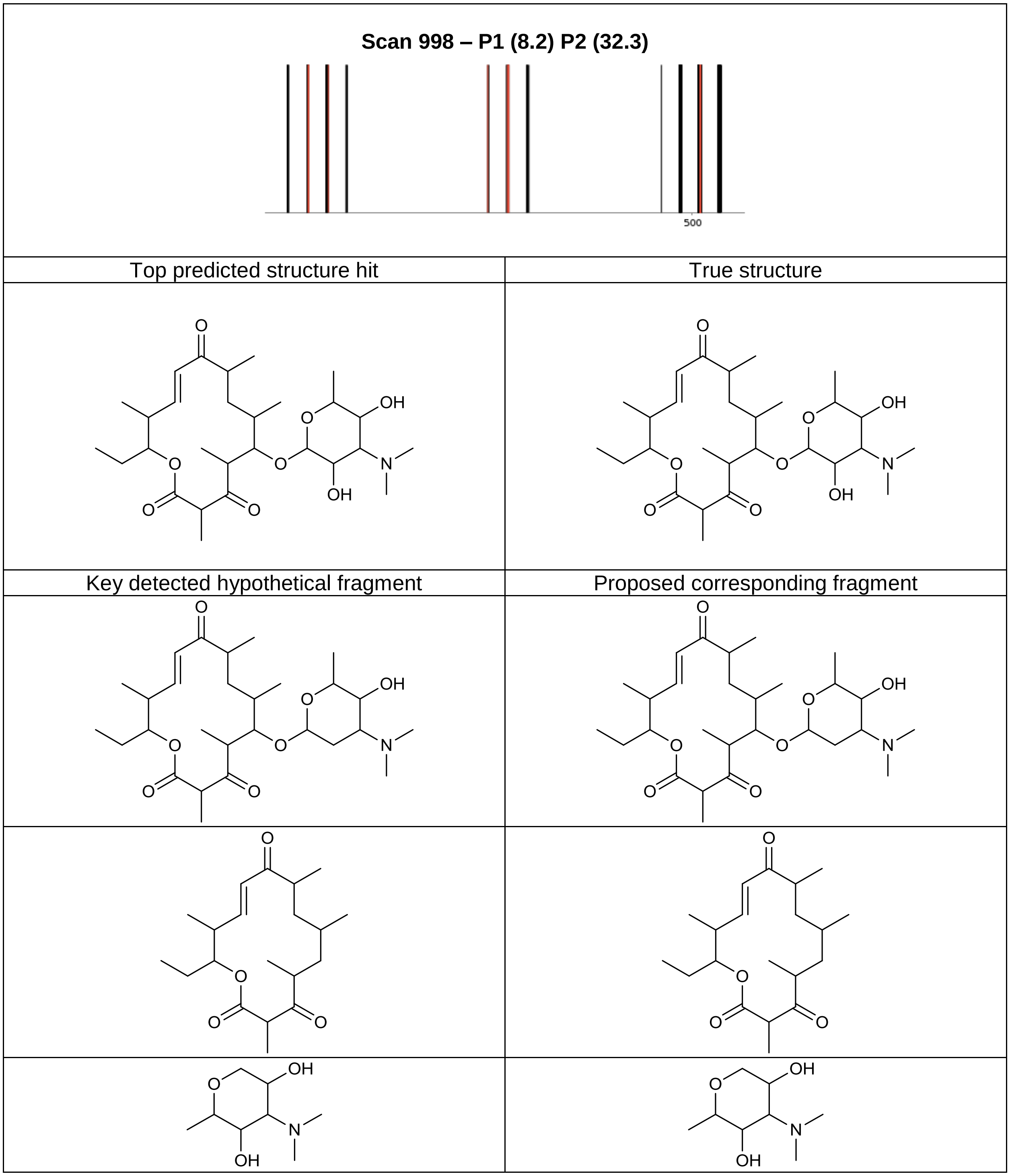

Supplementary Figure 26 Sample matched fragments between a predicted structure hit and potensimicin. Several matched fragments between the hypothetical compound hit and the corresponding true structure are shown. Fragments of potensimicin were generated using the GNP Fragmenter application. Detected hypothetical spectral fragments are shown as lines in the diagram above, with detected masses shown in red. P-scores for the scan are listed above. Full results tables for this scan and experimental run are available at http://magarveylab.ca/data/gnp.

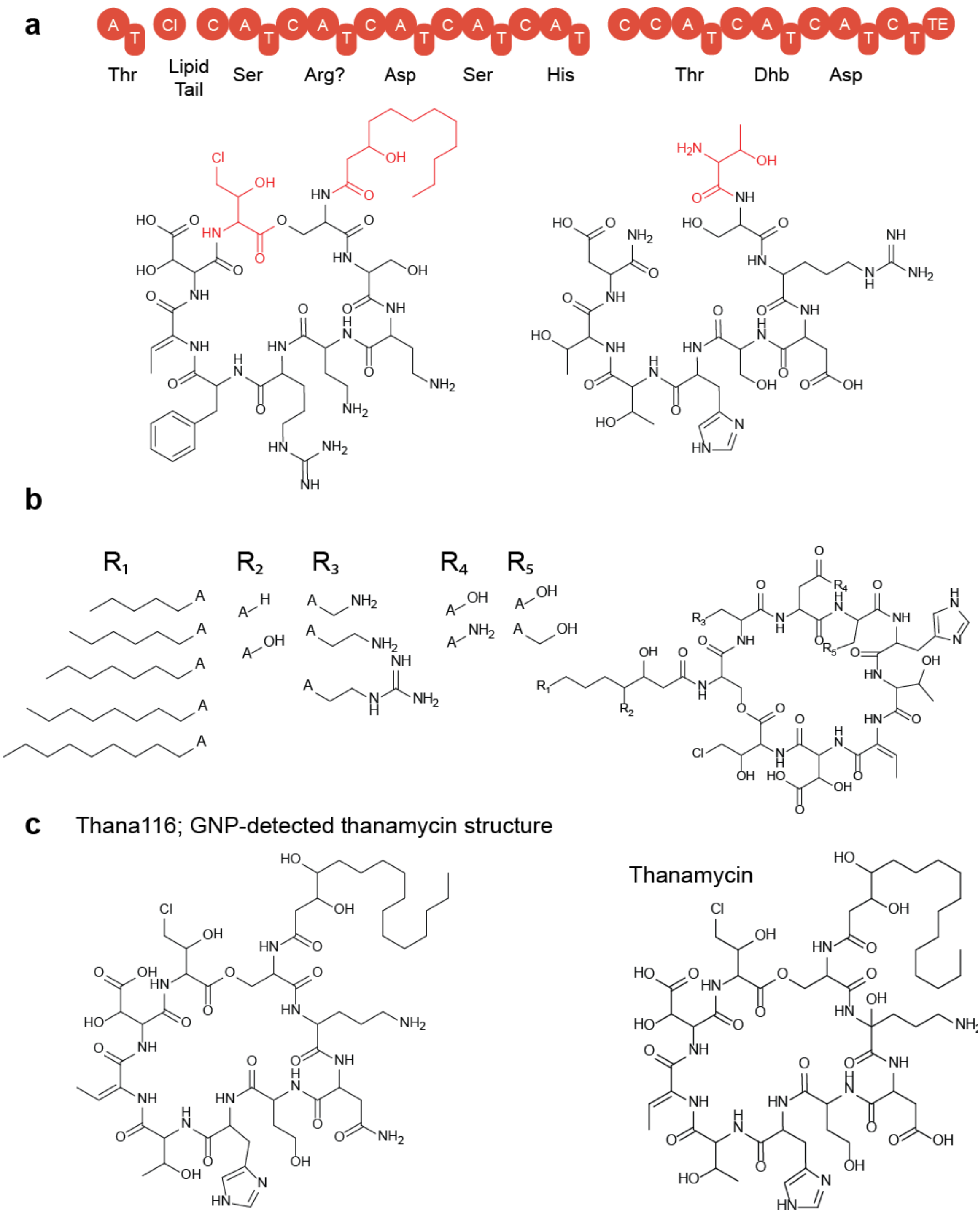

Supplementary Figure 27 Prediction, combinatorialization, and detection of thanamycin metabolites. (**a**) GNP detected thanamycin biosynthetic gene cluster from *Pseudomonas fluorescens* DSM 11579 (90% pairwise identity with *Pseudomonas* SH-C52 ThaAB), with predicted monomers listed and GNP-generated prediction shown (*right*) beside the related syringomycin structure (*left*). (**b**) Structures used for combinatorialization, taking into account the initial acylating condensation domain and trans-acting chlorinated threonine adenylation domain, as well as conserved syringomycin-family modifications. (**c**) Structure of the most frequent hit detected within the *P. fluorescens* DSM 11579 LC-MS/MS data.

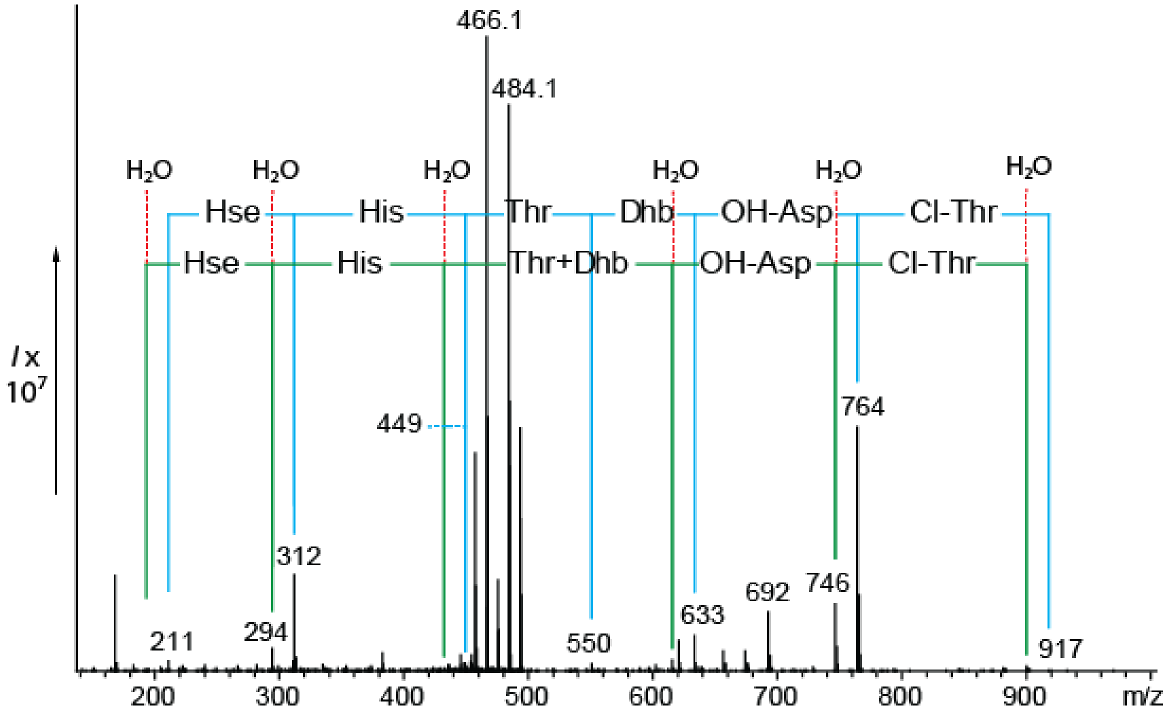

Supplementary Figure 28 Thanamycin tandem MS. MS/MS of the deacylated and dehydrated 1004 $[M+H]^+$ thanamycin fragment with amino acid annotation.

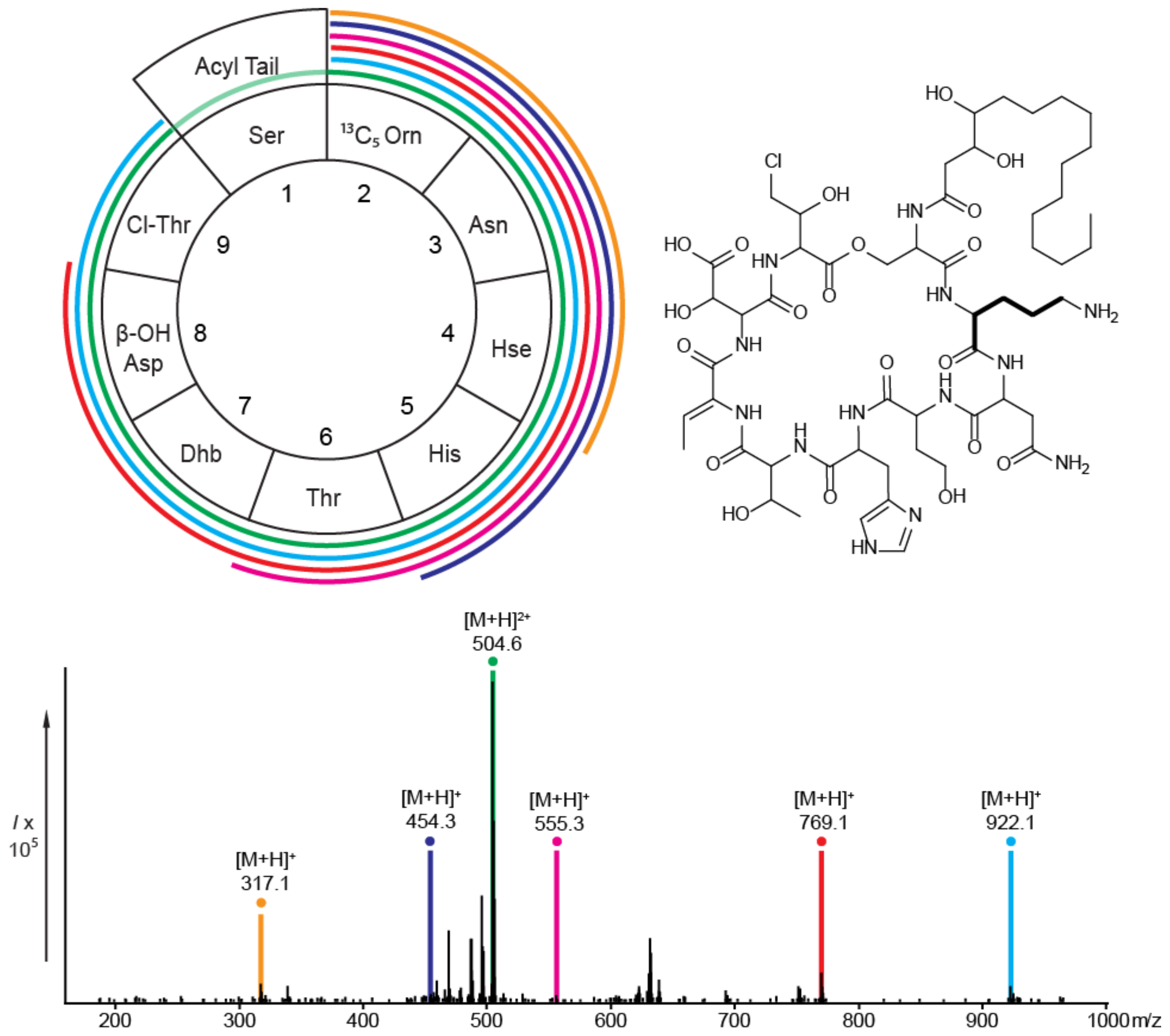

Supplementary Figure 29 GNP analysis of $^{13}C_5$-ornithine incorporation indicates ornithine at position 2. GNP analysis of extracted cultures containing stable $^{13}C_5$-ornithine identified matched fragments corresponding thanamycin with heavy ornithine at position 2 (*top left*, *bottom*), resulting in the matched structure (*top right*). MS/MS spectra and matched fragments are from representative scan 913.

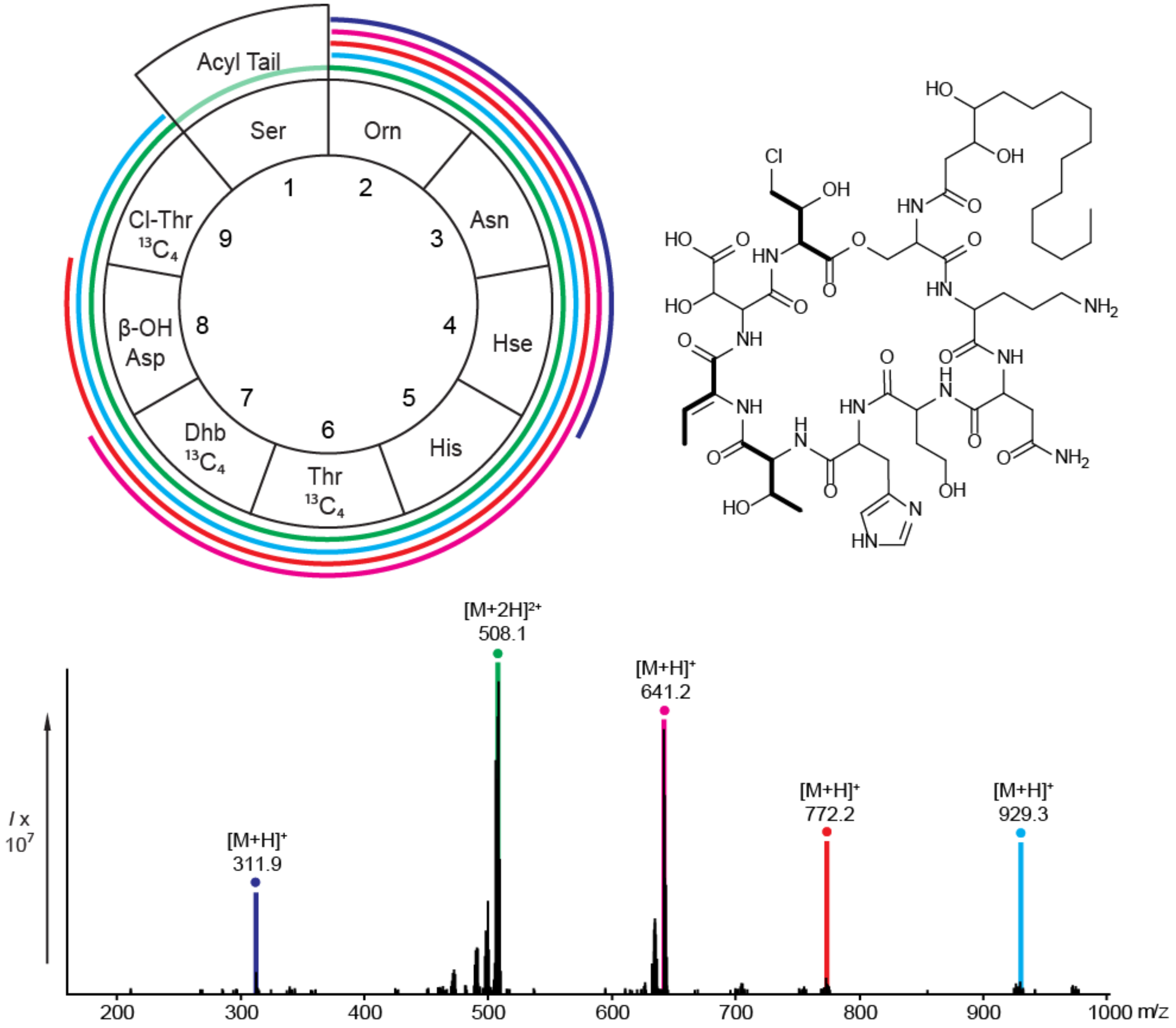

Supplementary Figure 30 GNP analysis of $^{13}C_4$-threonine incorporation indicates homoserine at position 4. GNP analysis of extracted cultures containing stable $^{13}C_4$-threonine identified matched fragments corresponding thanamycin with heavy threonine or threonine derivatives (Dhb) at positions 6, 7, and 9 (*top left, bottom*). No fragments were detected that indicated incorporation at position 4, resulting in the matched structure with homoserine found at position 4 (*top right*). MS/MS spectra and matched fragments are from representative scan 1148.

a

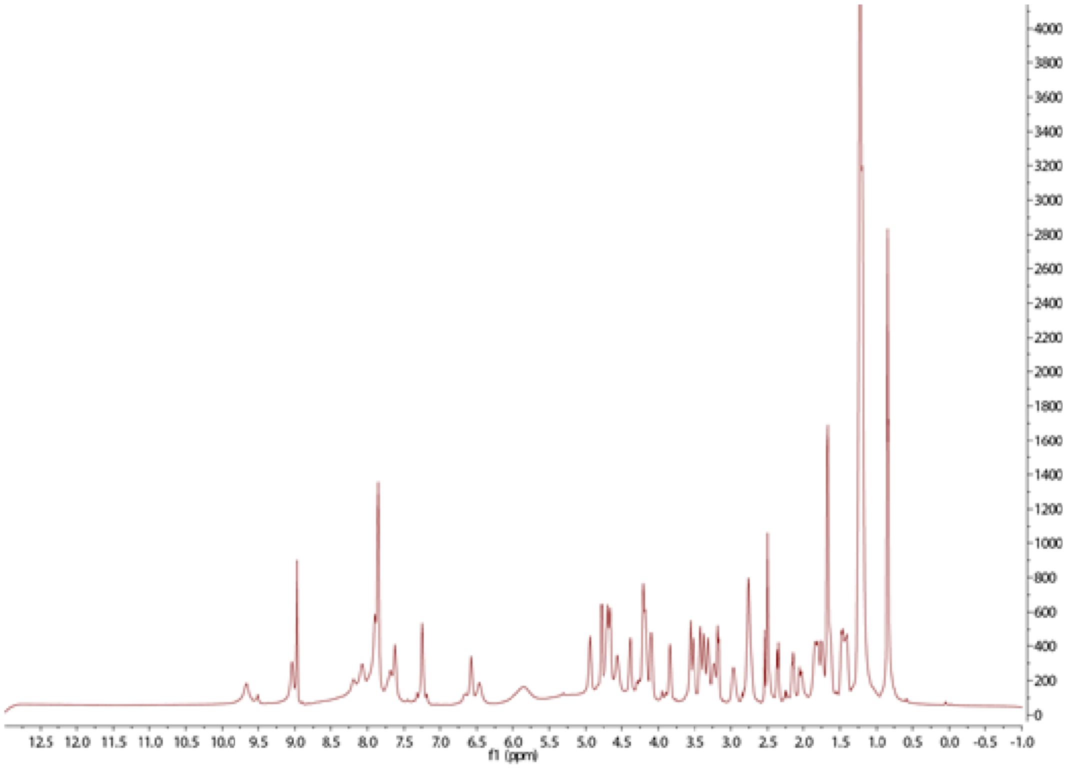

b

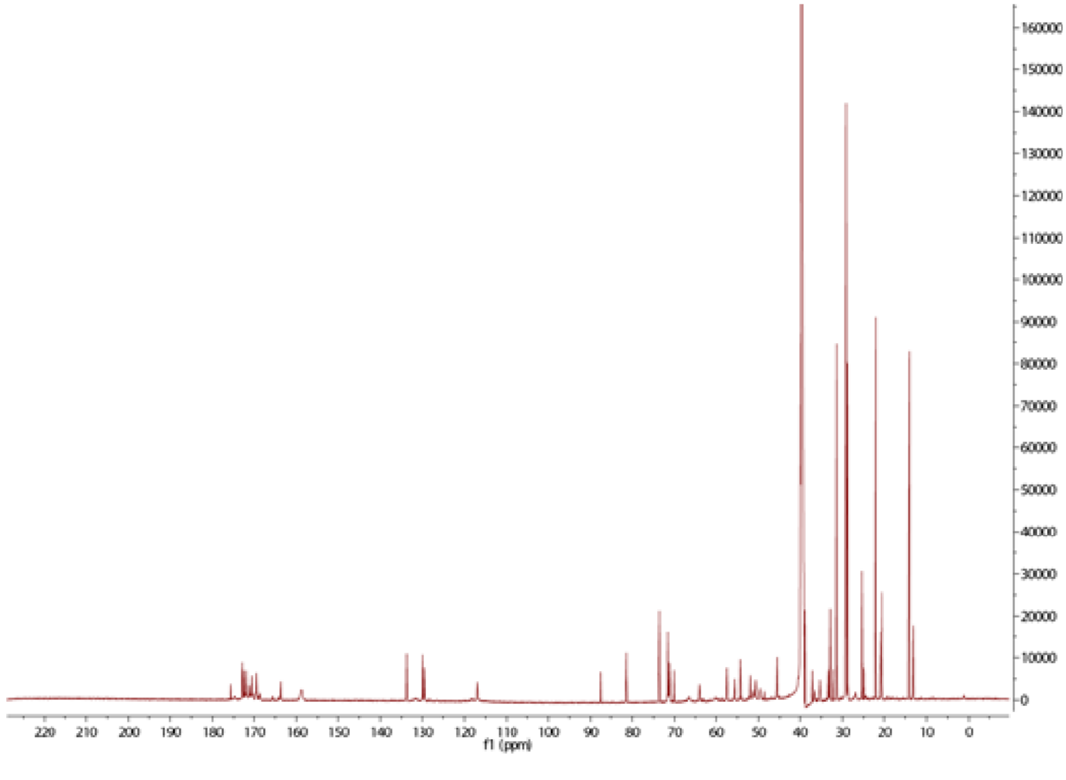

c

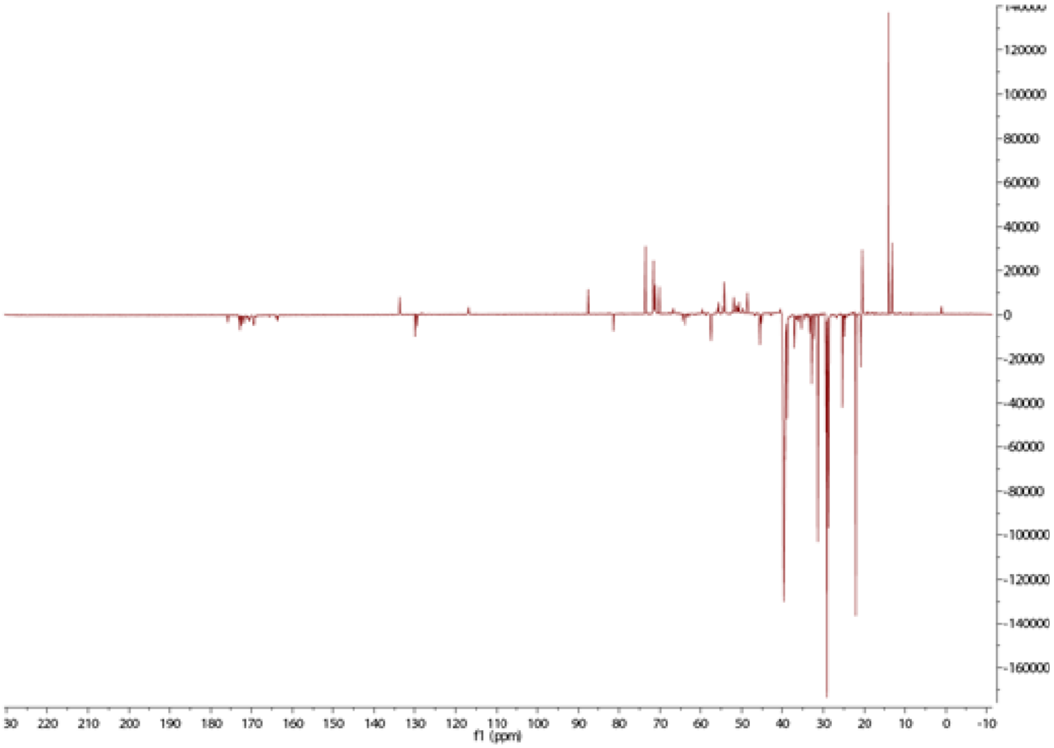

d

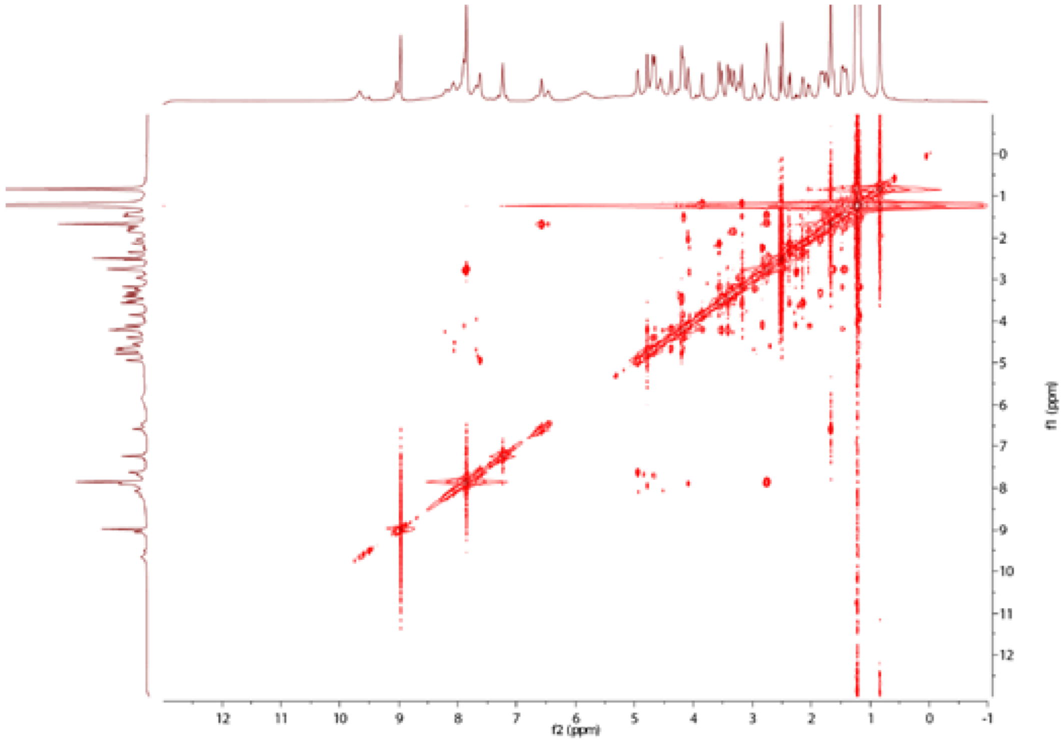

e

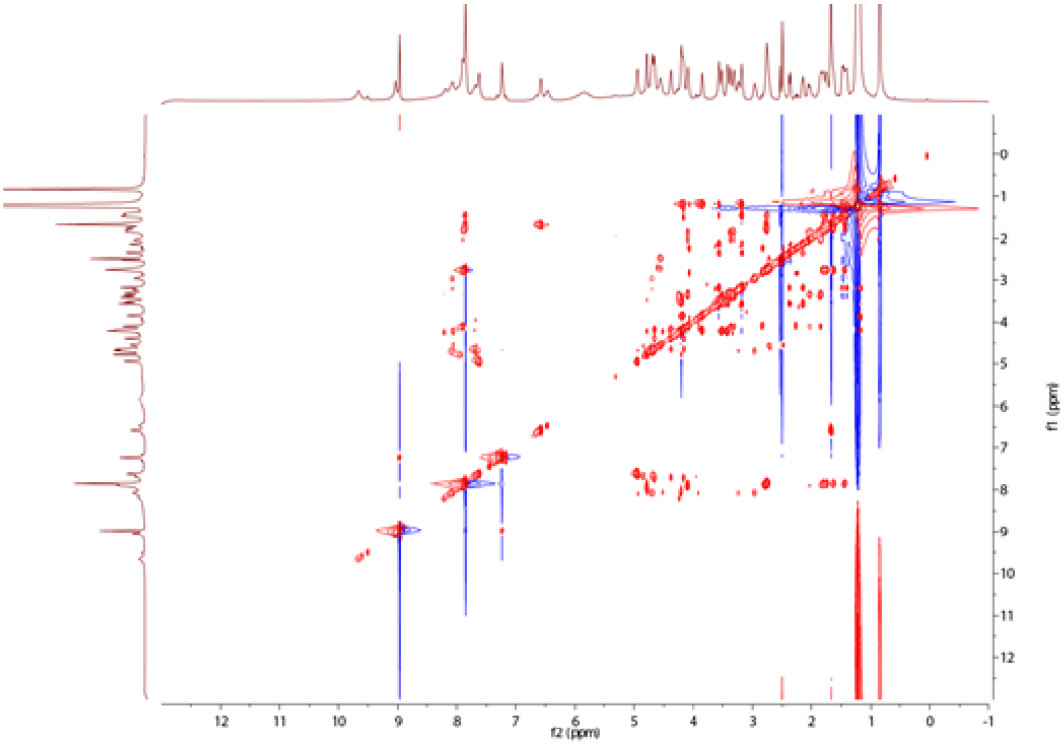

f

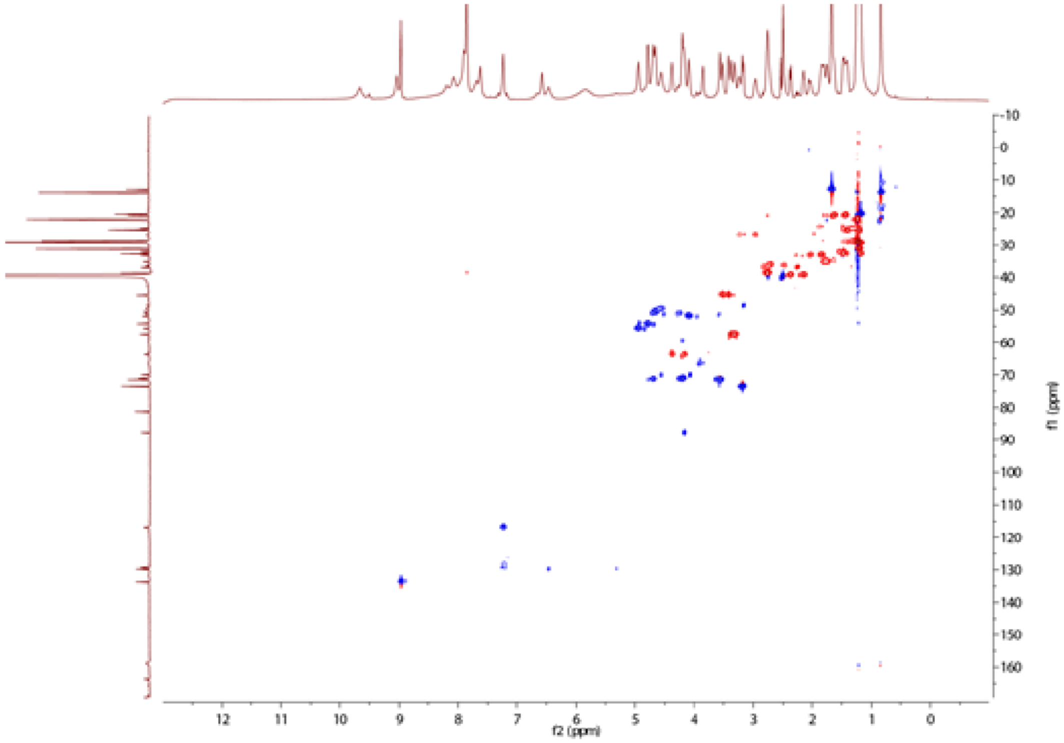

g

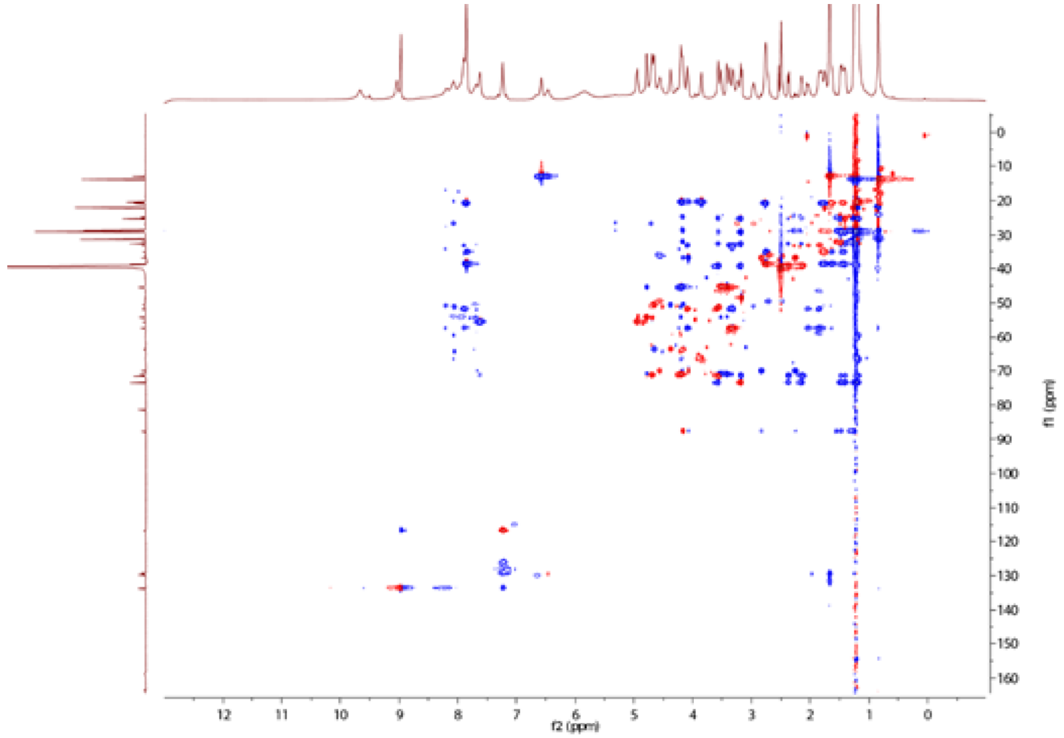

h

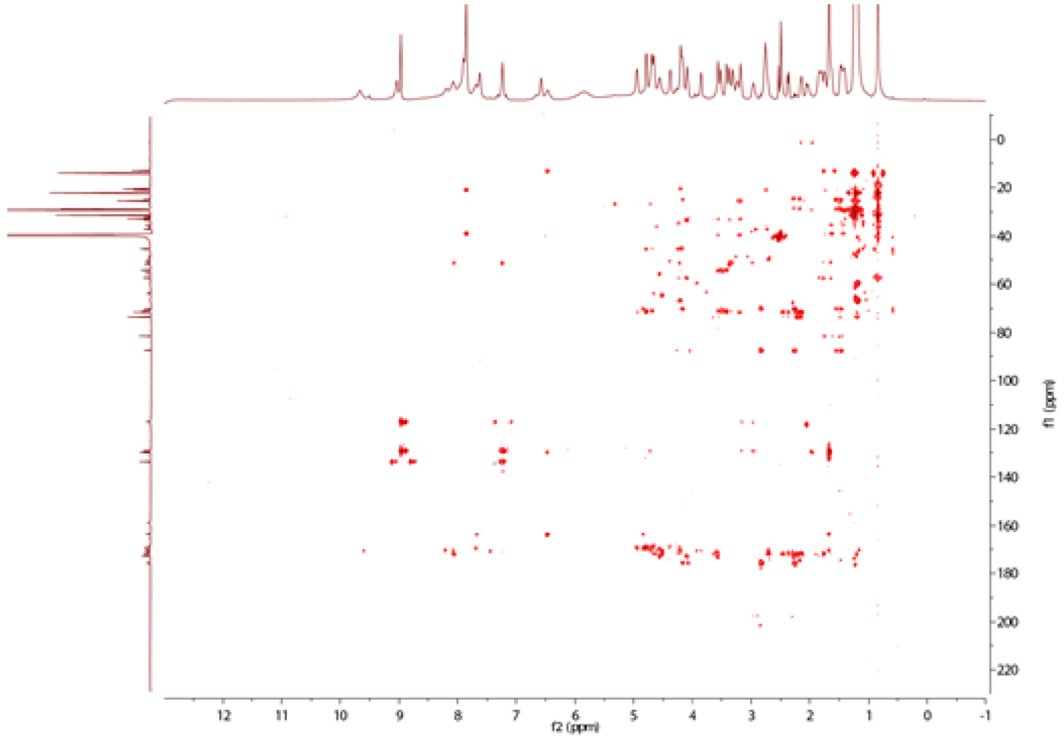

i

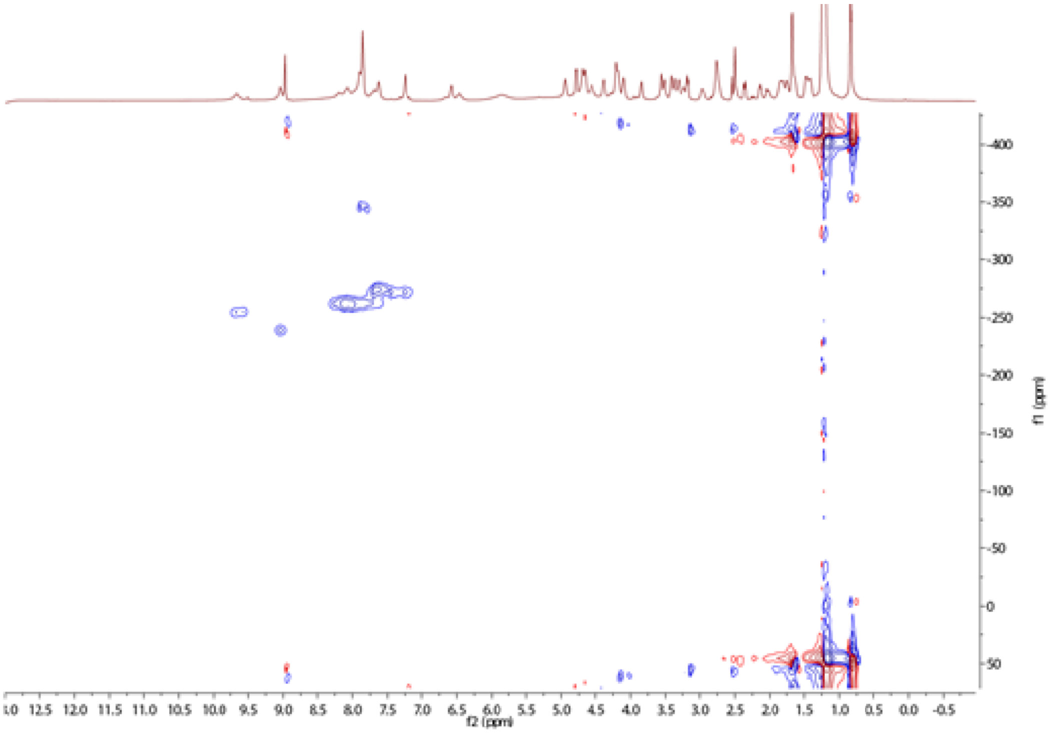

j

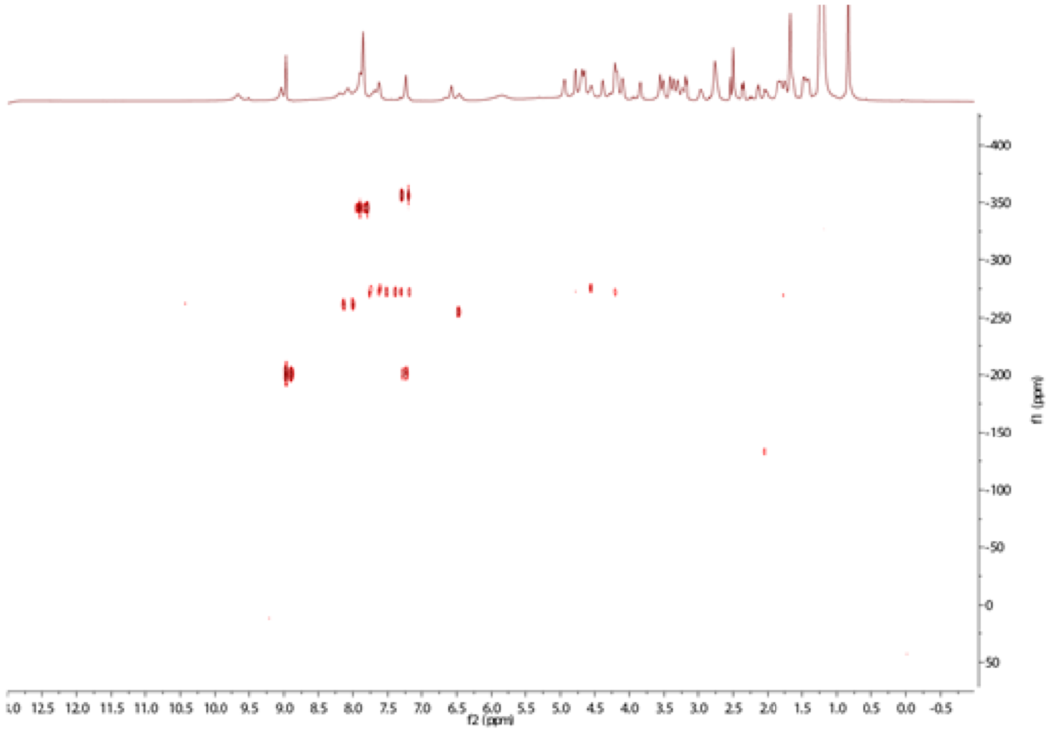

k

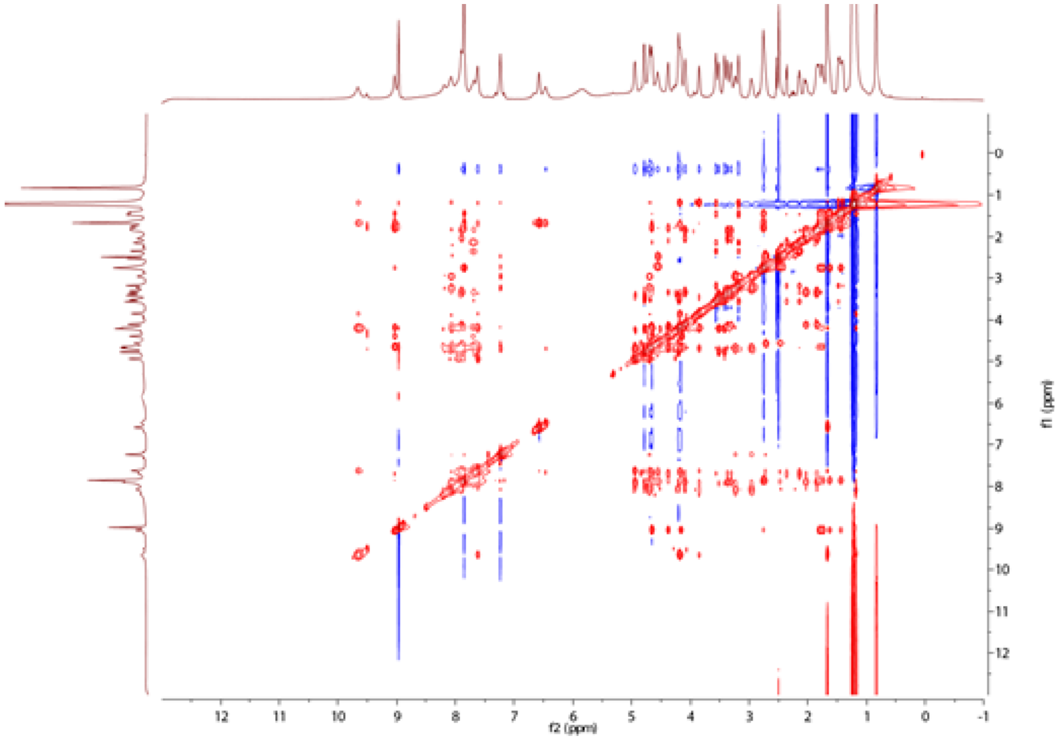

Supplementary Figure 31 NMR analysis of thanamycin (**8**) in DMSO. (**a**) ^{1}H NMR spectrum. (**b**) ^{13}C NMR spectrum. (**c**) ^{13}C DEPTq spectrum. (**d**) ^{1}H-^{1}H COSY spectrum. (**e**) ^{1}H-^{1}H TOCSY spectrum. (**f**) ^{1}H-^{13}C HSQC spectrum. (**g**) ^{1}H-^{13}C HSQC-TOCSY spectrum. (**h**) ^{1}H-^{13}C HMBC spectrum. (**i**) ^{1}H-^{15}N HSQC spectrum. (**j**) ^{1}H-^{15}N HMBC spectrum. (**k**) ^{1}H-^{1}H NOESY spectrum.

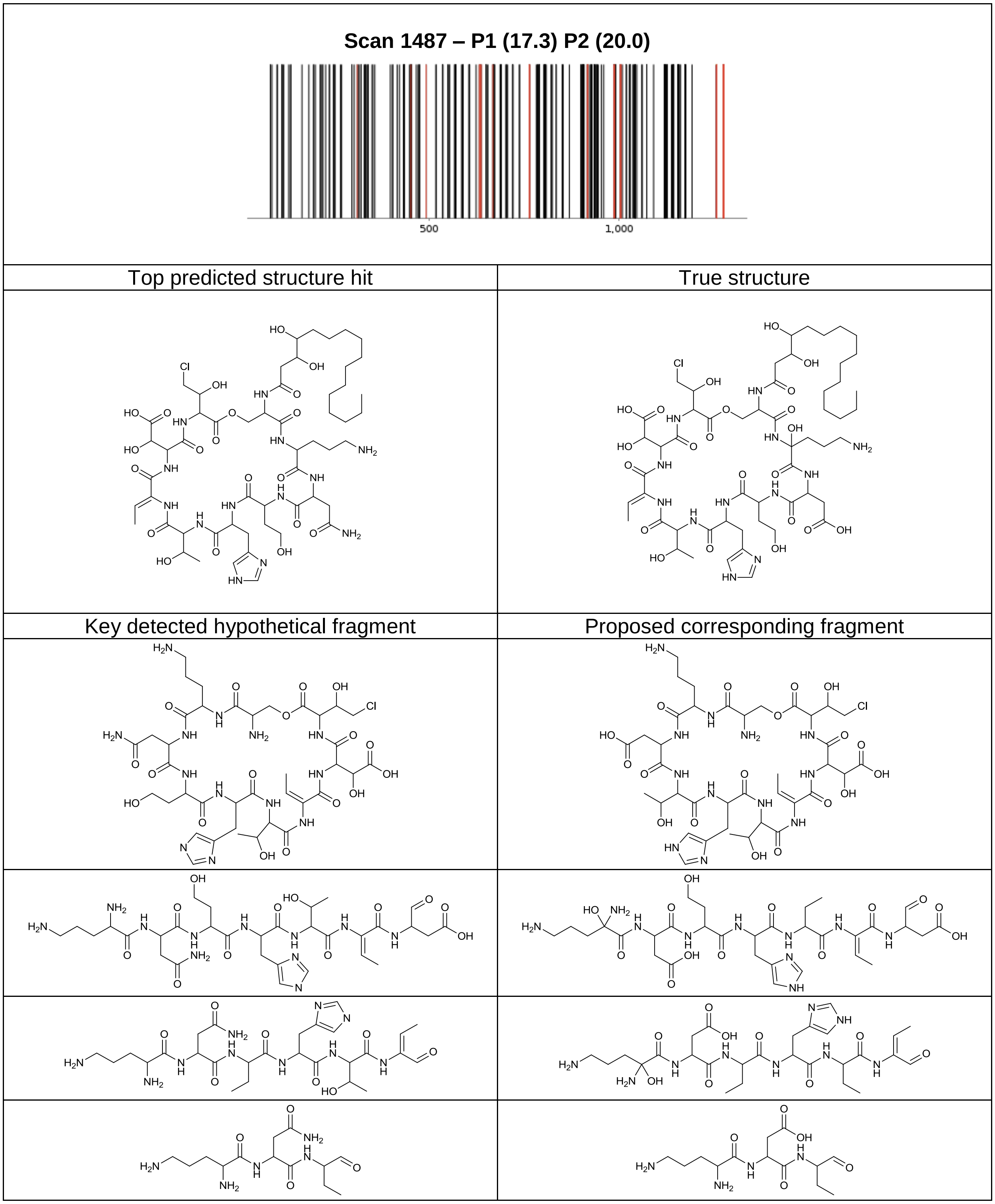

Supplementary Figure 32 Sample matched fragments between a predicted structure hit and thanamycin. Several matched fragments between the hypothetical compound hit and the corresponding true structure are shown. Fragments of thanamycin were generated using the GNP Fragmenter application. Detected hypothetical spectral fragments are shown as lines in the diagram above, with detected masses shown in red. P-scores for the scan are listed above. Full results tables for this scan and experimental run are available at http://magarveylab.ca/data/gnp.

Source	Program	Natural Product Classes	Automated NRP structure prediction?	Automated RiPP structure prediction?	Automated sugar structure prediction?	Automated polyketide structure prediction?	Automated MS analysis?	Statistical genome - metabolome matching?	Single software package?	User-friendly interface?
This paper	GNP	Glycosylated Polyketides, NRPs	**Yes**	No	**Yes**	**Yes**	**Yes**	**Yes**	**Yes**	**Yes**
55	NRPQuest	NRPs	**Yes**	No	No	No	**Yes**	**Yes**	**Yes**	No
54	RiPPQuest	RiPPs	No	**Yes**	No	No	**Yes**	**Yes**	**Yes**	No
20	Pep2Path	NRPs, RiPPs	No	No	No	No	No	**Yes**	No	No
21	Peptidogenomics	NRPs, RiPPs	No	No	No	No	No	No	No	No
22	Glycogenomics	Glycosylated NPs	No	No	No	No	No	No	No	No
57	HSEE	RiPPs	No	No	No	No	No	**Yes**	No	No

Supplementary Figure 33 Comparison of GNP to several manual, automated, or semi-automated genomic and metabolomic natural products discovery methods. GNP expands the chemical search space of previously published methods by targeting a wider spectrum of natural product classes, and automates structure prediction for modular natural products. While previously published methods require the annotation of mass shifts within a tandem mass spectrum scan[20-22], or the selection of a single point on a high-resolution mass spectrum[57], GNP's integrated mass spectral analysis algorithms eliminate the need for manual mass spectrum annotation and therefore facilitates high-throughput discovery. Finally, as a continuous workflow integrated into a single web application with a user-friendly interface, GNP is accessible to natural products chemists without formal bioinformatics training. By automating the most labour-intensive steps associated with a combined genomic and metabolomic discovery approach, GNP has the potential to significantly accelerate natural product discovery. NRP, nonribosomal peptide; RiPP, ribosomally synthesized and post-translationally modified peptide; NP, natural product.

Supplementary Tables

Gene name	Predicted Function	Strand	Amino Acids
cal1	Aminotransferase	-	414
cal2	LuxR transcriptional regulator	-	215
cal3	LysR transcription factor	-	80
cal4	MbtH domain protein	-	73
cal5	Thioesterase	-	263
cal6	Hypothetical protein	+	319
cal7	3-oxoacyl-ACP synthase	+	335
cal8	3-oxoacyl-ACP synthase	+	414
cal9	Acyl-carrier protein	-	84
cal10	Hypothetical protein	-	124
cal11	Hypothetical protein	-	137
cal12	Translation initiation factor IF-2	-	141
cal13	3-hydroxylacyl-ACP dehydratase	-	315
cal14	Hypothetical protein	+	258
cal15	Ferredoxin	+	66
cal16	Cytochrome P450	+	408
cal17	NRPS	-	2568
cal18	NRPS	-	2577
cal19	NRPS	-	1892
cal20	Thioesterase	-	234
cal21	Short chain dehydrogenase/reductase	-	276
cal22	Adenylation-ACP didomain	-	979
cal23	Adenylation-ACP didomain	-	600
cal24	Oxidoreductase	-	400
cal25	Hydrolase	-	275
cal26	Putative thioesterase	-	334
cal27	Isomerase	-	242
cal28	Phytoene dehydrogenase	-	575
cal29	Acyl-carrier protein	-	87
cal30	3-oxoacyl-ACP synthase	-	417
cal31	3-oxoacyl-ACP synthase	-	380
cal32	3-oxoacyl-ACP synthase	-	315
cal33	3-oxoacyl-ACP synthase	-	372
cal34	Acyl-carrier protein	-	88
cal35	3-oxoacyl-ACP dehydratase	-	134
cal36	3-oxoacyl-ACP dehydratase	-	160
cal37	3-oxoacyl-ACP reductase	-	249
cal38	YbaK/prolyl-tRNA synthetase	-	179
cal39	ABC transporter ATPase	-	319
cal40	Multidrug ABC transporter permease	-	281

Supplementary Table 1 WS9326 gene cluster analysis

Supplementary Table 2 Summary of ^{1}H (600 MHz) and ^{13}C (125 MHz) spectroscopic data for WS9326a (1) in DMSO-d_6[a]

Position		d_H (*J* in Hz)	d_C
Acyl	1 (C=O)		165.2, s
	2	6.68, 1H, d (15.6)	122.7, d
	3	7.42, 1H, d (15.6)	137.4, d
	4		133.1, s
	5	7.53, 1H, d (7.6)	126.0, d
	6	7.32, 1H, o. m	127.3, d
	7	7.36, 1H, o. t (7.6)	128.9, d (o)
	8	7.20, 1H, o. m	129.6, d
	9		137.0, s
	10	6.50, 1H, d (11.2)	126.8, d
	11	5.83, 1H, dt (11.7, 7.4)	134.0, d
	12	1.99, 2H, m	29.9, t
	13	1.36, 2H, m (7.8)	21.9, t
	14	0.79, 3H, t (7.4)	13.53, q
^{1}Thr	NH	8.70, 1H, d (9.3)	
	a	5.33, 1H, t (9.6)	53.2, d
	b	5.03, 1H, dq (9.8, 6.2)	73.24, d
	g	1.15, 3H, d, 6.0	16.56, q
	C=O		169.0, s
^{2}DTyr	NMe	2.98, 3H, s	34.2, q
	a		128.5, s
	b	6.13, 1H, s	131.6, d
	1		122.9, s
	2,6	7.39, 2H, d (8.6)	131.5, d
	3,5	6.59, 2H, d (8.6)	114.8, d
	4		158.1, s
	C=O		165.6, s
^{3}Leu	NH	9.23, 1H, br. s	
	a	4.07, 1H, m	53.7, d
	b	1.26, 2H, m	38.8, t
	g	0.89, 1H, m	23.4, d
	d	0.63, 3H, o. d	22.8, q
	d'	0.75, 3H, d (6.3)	22.0, q
	C=O		172.1, s

Position		d_H (*J* in Hz)	d_C
^{4}Phe	NH	9.16, 1H, d (7.7)	
	a	4.33 m	55.7, d
	b	3.28, 1H, m	36.3, t
	b'	2.73, 1H, o. t (12.9)	
	1		138.7, s
	2,6	7.32, 2H, o. d (8.4)	128.9, d (o)
	3,5	7.27, 2H, o. t, (7.6)	127.9, d (o)
	4	7.27, 1H, o. m	127.9, d (o)
	C=O		170.1, s
^{5}Thr	NH	7.59, 1H, d (9.5)	
	a	4.36, 1H, =tho. t (9.8)	57.2, d
	b	4.26, 1H, m	68.1, d
	g	0.64, 3H, o. d (6.4)	22.0, q
	OH	5.18, 1H, d (2.9)	
	C=O		169.9, s
^{6}Asn	NH	8.33, 1H, d (7.3)	
	a	4.44, 1H, m	50.8, d
	b	2.46, 1H, m	36.7, t
	b'	2.41, 1H, dd (15.3, 9.9)	
	g C=O		171.2, s
	g NH_2	6.93, 1H, s	
	g' NH_2	7.30, 1H, o	
	C=O		171.6, s
^{7}Ser	NH	8.48, 1H, d (9.6)	
	a	4.33, 1H, o	56.0, d
	b	3.26, 1H, o	60.8, d
	b'	3.16, 1H, br. t (~9)	
	OH	4.78, 1H, br	
	C=O		168.8, s

[a]Assignments based on HSQC, COSY, TOCSY, HMBC, and ROESY experiments. o = overlapping signal, br = broad signal, n/d = no data

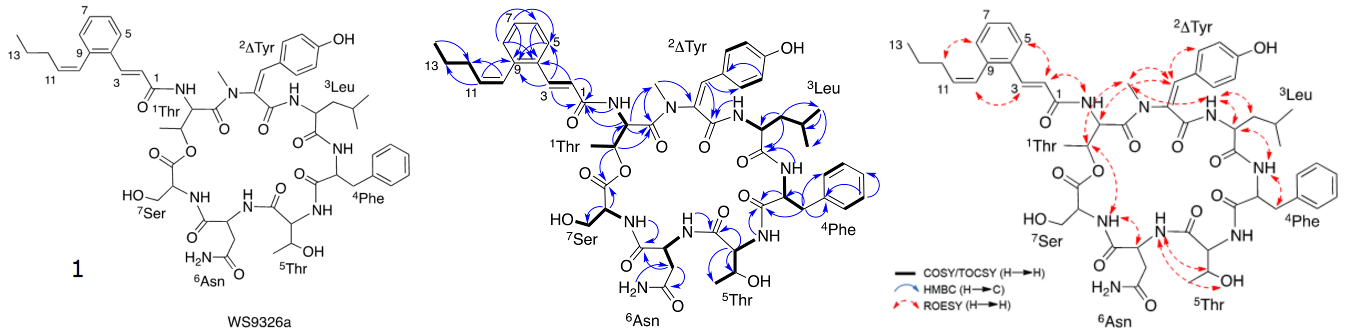

Supplementary Table 3 High Resolution Mass Data

Compound	***Molecular Formula***	***Calculated m/z***	***Observed m/z***	***Δppm***
Acidobactin A [M+H]	$C_{28}H_{48}N_7O_{16}$	738.31575	738.31705	5.245
Acidobactin B [M+H]	$C_{28}H_{48}N_7O_{15}$	722.32283	722.31768	2.609
Variobactin A [M+H]	$C_{47}H_{84}N_{11}O_{17}$	1074.60462	1074.6093	4.1
Potensimicin [M+H]	$C_{28}H_{48}NO_8$	526.33744	526.33559	4.565
Thanamycin A [M+H]	$C_{54}H_{88}ClN_{12}O_{22}$	1291.58192	1291.58220	0.205

Supplementary Table 4 Acidobactin gene cluster analysis

**Locus**	**Predicted Function**	**Strand**	**Amino Acids**
Aave_3737	RNA polymerase, sigma-24 subunit, ECF subfamily	-	177
Aave_3736	MbtH domain protein	-	95
Aave_3735	Thioesterase	-	258
Aave_3734	TauD dioxygenase	-	330
Aave_3733	NRPS	-	1779
Aave_3732	PKS	-	1535
Aave_3731	NRPS	-	1130
Aave_3730	NRPS	-	2651
Aave_3729	NRPS	-	1368
Aave_3728	TonB siderophore receptor	+	733
Aave_3727	N-acetyltransferase	+	366
Aave_3726	N5-hydroxyornithine formyltransferase	+	313
Aave_3725	Ferric iron reductase	+	276
Aave_3724	Cyclic peptide transporter	-	564
Aave_3723	L-lysine 6-monooxygenase	-	452
Aave_3722	Phosphopantetheinyl transferase	-	257

Supplementary Table 5 NMR spectroscopic Data for acidobactin A (2), B (3), vacidobactin A (4), and B (5) (700 MHz, in D_2O)[a]

position	δ_H mult.				δ_C			
	**2**	**3**	**4**	**5**	**2** [a]	**3**	**4** [a]	**5** [a]
1	1.25, *d*, 4.0	1.20, *d*, 4.2	1.19, *d*, 3.6	1.19, *d*, 3.6	18.6, CH_3	19.3, CH_3	19.0, CH_3	19.0, CH_3
2	5.34, *m*	5.48, *m*	5.38, *m*	5.37, *m*	68.9, CH	70.7, CH	70.6, CH	70.7, CH
3a	2.51, *m*	2.73, *m*	2.58, *m*	2.58, *m*	34.9, CH_2	39.7, CH_2	38.4, CH_2	38.3, CH_2
3b	2.80, *m*	2.81, *m*	2.77, *m*	2.71, *m*	-	-	-	-
4	-	-	-	-	164.2, C	171.8, C	164.6, C	171.4, C
5	3.36, *m*	3.33, *m*	3.31, *m*	3.36, *m*	54.4, CH	54.9, CH	52.8, CH	52.9, CH
6a	1.61, *m*	1.50, *m*	1.63, *m*	1.63, *m*	23.1, CH_2	23.7, CH_2	22.4, CH_2	22.6, CH_2
6b	1.59, *m*	1.68, *m*	-	-	-	-	-	-
7a	1.71, *m*	1.63, *m*	1.83, *m*	1.87, *m*	25.4, CH_2	26.4, CH_2	28.6, CH_2	28.4, CH_2
7b	1.80, *m*	1.85, *m*	-	-	-	-	-	-
8a	3.55, *m*	3.49, *m*	3.38, *m*	3.33, *m*	49.9, CH_2	49.8, CH_2	52.1, CH_2	52.7, CH_2
8b	-	3.90, *m*	-	3.48, m	-	-	-	-
9	7.90, *s*	7.88, *s*	7.74, *s*	7.74, *s*	153.6, CH	160.0, CH	155.4, CH	155.5, CH
10	4.22, *m*	4.35, *m*	4.30, m	4.17, m	68.0, CH	71.9, CH	72.3, CH	66.9, CH
11a	2.79, *m*	2.59, *m*	2.71, *m*	2.69, *m*	39.0, CH_2	38.9, CH_2	43.2, CH	43.1, CH
11b	-	2.69, *m*	-	-	-	-	-	-
11-CH_3	-	-	1.03, *d*	1.02, *d*	-	-	12.8, CH_3	12.7, CH_3
12	-	-	-	-	173.4, C	172.3, C	177.0, C	177.1, C
13	4.23, *m*	4.40, *d*, 1.8	4.41, *m*	4.44, *m*	58.7, CH	60.8, CH	56.2, CH	55.6, CH
14	4.22, *m*	4.31, *m*	4.26, *m*	4.19, *m*	66.3, CH	66.5, CH	66.8, CH	66.4, CH
15	1.10, *d*, 3.8	1.07, *d*, 4.0	1.10, *m*	1.08, *m*	18.8, CH_3	19.1, CH_3	19.2, CH_3	19.1, CH_3
16	-	-	-	-	174.1, C	174.1, C	172.3, C	173.9, C
17	4.36, *m*	4.60, *t*, 3.6	4.40, *m*	4.41, *m*	53.6, CH	54.1, CH	55.3, CH	55.1, CH
18a	1.62, *m*	1.71, *m*	1.60, *m*	1.62, *m*	27.7, CH_2	27.9, CH_2	28.4, CH_2	28.3, CH_2
18b	1.84, *m*	2.61, *m*	-	-	-	-	-	-
19a	1.71, *m*	1.81, *m*	1.85, *m*	1.81, *m*	23.7, CH_2	22.1, CH_2	22.6, CH_2	22.4, CH_2
19b	1.87, *m*	2.12, *m*			-	-	-	-
20a	3.55, *m*	3.32, *m*	3.41	3.37	50.3, CH_2	49.3, CH_2	51.4, CH_2	49.8, CH_2
20b	-	3.96, *m*	-	3.65	-	-	-	-
21	-	-	-	-	172.9, C	174.2, C	172.7, C	174.9, C
22	4.41, *m*	4.44, *m*	4.30, *m*	4.27, *m*	56.3, CH	55.7, CH	54.7, CH	54.4, CH
23a	3.83, *d*, 8.5	3.84, *d*, 9.0	3.90, *m*	3.85, *m*	61.3, CH	61.1, CH	61.3, CH	61.5, CH
23b	-	3.95, *d*, 9.0	-	-	-	-	-	-
24	-	-	-	-	171.6, C	172.5, C	169.3, C	169.4, C
25	4.82, *m*	4.50, *m*	4.80, *m*	4.59, *m*	56.8, CH	56.2, CH	56.4, CH	51.9, CH
26	4.31, *m*	4.25, *m*	3.90, *m*	3.87, *m*	74.7, CH	72.4, CH	73.9, CH	73.6, CH
27	-	-	-	-	180.0, C	175.6, C	179.6, C	181.4, C
28	-	-	-	-	169.6, C	169.1, C	177.9, C	177.7, C

[a] ^{13}C NMR were extracted from HMBC spectra.

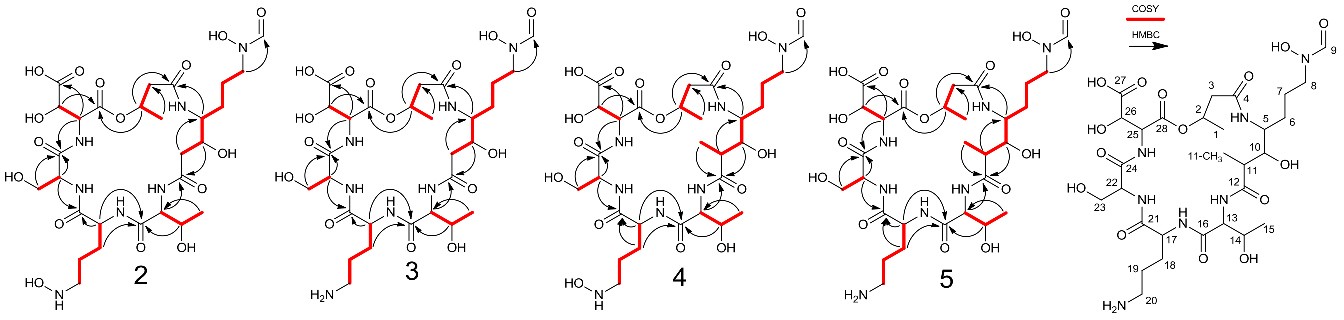

Supplementary Table 6 Vacidobactin gene cluster analysis

Locus	Predicted Function	Strand	Amino Acids
Vapar_3752	RNA polymerase, sigma-24 subunit, ECF subfamily	-	181
Vapar_3751	anti-FecI sigma factor	-	67
Vapar_3750	MbtH domain protein	-	85
Vapar_3749	Thioesterase	-	246
Vapar_3748	Phosphopantetheinyl transferase	-	229
Vapar_3747	TauD dioxygenase	-	330
Vapar_3746	NRPS	-	1776
Vapar_3745	PKS	-	1520
Vapar_3744	NRPS	-	1110
Vapar_3743	NRPS	-	2626
Vapar_3742	NRPS	-	1358
Vapar_3741	TonB siderophore receptor	+	723
Vapar_3740	L-lysine 6-monooxygenase	+	439
Vapar_3739	N-acetyltransferase	+	344
Vapar_3738	N5-hydroxyornithine formyltransferase	+	281
Vapar_3737	Ferric iron reductase	+	281
Vapar_3736	Cyclic peptide transporter	-	563

Supplementary Table 7 Variobactin gene cluster analysis

Locus	Predicted Function	Strand	Amino Acids
Var1	Thioesterase	+	278
Var2	Phosphopantetheinyl transferase	+	234
Var3	NRPS	+	1741
Var4	PKS	+	2346
Var5	NRPS	+	1038
Var6	NRPS	+	2585
Var7	NRPS	+	2431
Var8	TonB receptor	-	816
Var9	FecR-like protein	-	343
Var10	RNA polymerase subunit sigma-24	-	193
Var11	L-lysine 6-monooxygenase	-	440
Var12	N-acetyltransferase	-	369
Var13	Ferric iron reductase	-	262
Var14	Cyclic peptide transporter	+	560

Supplementary Table 8 NMR spectroscopic data for variobactin A (6) (700 MHz, in DMSO-d_6)[a].

Position	δ_H mult.	δ_C	Position	δ_H mult.	δ_C
1	0.89 (*t*, 7.2)	14.4, CH_3	34	-	-
2	1.23 (*ov*)	22.5, CH_2	35a	3.81 (*m*)	49.4, CH_2
3	1.23 (*ov*)	31.7, CH_2	35b	3.36 (*m*)	
4-9	1.23 (*ov*)	28.5-29.5, CH_2	36	2.06 (*m*)	23.7, CH_2
10	1.45 (*m*)	25.9, CH_2	37a	1.45 (m)	29.3, CH_2
11	2.04 (*m*)	36.0, CH_2	37b	1.14 (m)	
12	-	172.2, C	38	3.59 (*ov*)	50.4, CH
13	7.84 (*broad*)	-	39	-	171.1, C
14	2.03 (*s*)	16.6, CH_3	40	7.78 (*d*, 9.9)	-
15	-	159.8, C	41	-	-
16a	3.90 (*m*)	47.2, CH_2	42	-	157.3, C
16b	3.70 (*m*)		43	8.06 (*broad*)	-
17	1.85 (*m*)	23.7, CH_2	44a	2.94 (*m*)	40.9, CH_2
18a	1.85 (*m*)	29.0, CH2	44b	3.15 (*m*)	
18b	1.56 (*m*)		45a	1.22 (*m*)	25.5, CH_2
19	4.36 (*m*)	49.9, CH	45b	1.46 (*m*)	
20	3.40 (*m*)	74.5, CH	46a	2.37 (*m*)	28.5, CH_2
21	2.14 (*m*)	44.3, CH	46b	1.63 (*m*)	
22	1.26 (*ov*)	16.8, CH_3	47	4.64 (*m*)	50.5, CH
23	-	175.3, C	48	-	172.1, C
24	9.04 (*d*, 7.6)	-	49	-	-
25	-	179.6, C	50	1.99 (*s*)	16.9, CH_3
26	4.29 (*d*, 2.1)	74.1, CH	51	-	161.7, C
27	4.19 (*d*, 9.3)	58.8, CH	52a	3.66 (*m*)	48.8, CH_2
28	-	174.4, C	52b	3.32 (*m*)	
29	6.89 (*d*, 4.9)	-	53a	1.74 (*m*)	22.0, CH_2
30a	3.70 (*d*, 5.7)	62.9, CH_2	53b	1.28 (*m*)	
30b	3.18 (*m*)		54a	1.99 (*m*)	30.1, CH_2
31	4.52 (*m*)	53.8, CH	54b	1.86 (*m*)	
32	-	170.3, C	55	4.20 (*d*, 9.3)	61.3, CH
33	7.26 (*d*, 8.66)	-	56	-	170.6, C

[a]Chemical shift δ and (multiplicity, *J* in Hz).

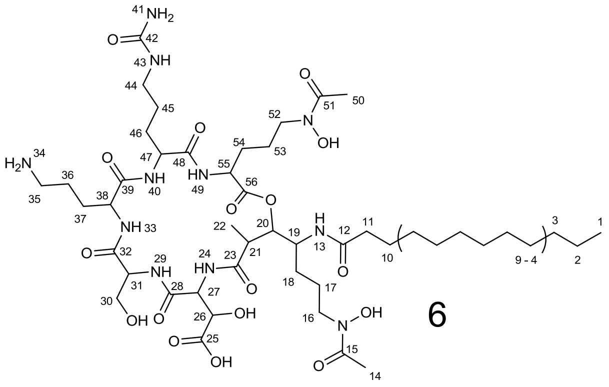

6

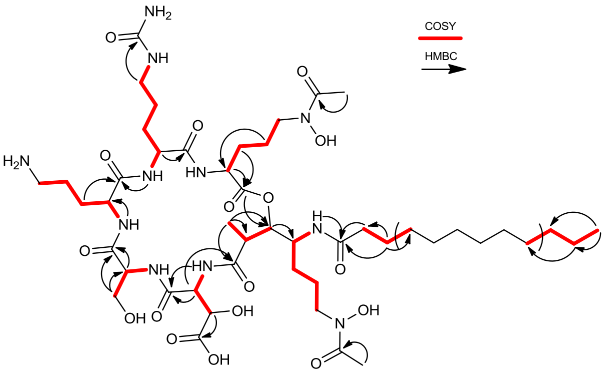

Supplementary Table 9 Hidden Markov models for sugar biosynthesis

Domain	Abbreviation	Hidden Markov model	Number of sequences	Cutoff
**2,3-dehydratase**	2,3DH	2_3_dehydratase.hmm	23	100.0
**3,4-dehydratase**	3,4DH	3_4_dehydratase.hmm	10	600.0
**3,4-ketoisomerase**	3,4IM	3_4_ketoisomerase.hmm	8	150.0
**3-aminotransferase**	3-AmT	3_aminotransferase.hmm	13	451.0
**3-ketoreductase**	3KR	3_ketoreductase.hmm	15	200.0
**4,6-dehydratase**	4,6DH	4_6_dehydratase.hmm	21	400.0
**4-aminotransferase**	4-AmT	4_aminotransferase.hmm	9	350.0
**4-ketoreductase**	4KR	4_ketoreductase.hmm	26	190.0
**Acetyltransferase**	AcT	acetyltransferase.hmm	4	160.0
**Carbamoyltransferase**	CarbT	carbamoyltransferase.hmm	2	845.0
**Decarboxylase**	UDP-DC	decarboxylase_epimerase.hmm	15	400.0
**Dehydrogenase**	UDP-DH	pentose_dehydrogenase.hmm	15	400.0
**Epimerase**	E	epimerase.hmm	22	160.0
**Glycosyltransferase**	GTr	glycosyltransferase.hmm	82	125.0
**N-ethyltransferase**	N-ET	n_ethyltransferase.hmm	2	500.0
**N,N-dimethyltransferase**	N,N-MT	n_n_dimethyltransferase.hmm	10	147.0
**Oxidative deaminase**	OxDA	oxidative_deaminase.hmm	10	500.0
**Oxidoreductase**	Ox	oxidoreductase.hmm	6	500.0
**Pyrrolyltransferase**	PyT	pyrrolyltransferase.hmm	4	350.0
**C-methyltransferase**	CMT	sugar_c_methyltransferase.hmm	20	400.0
**N-methyltransferase**	NMT	sugar_n_methyltransferase.hmm	5	180.0
**O-methyltransferase**	OMT	sugar_o_methyltransferase.hmm	40	147.0
**Thiosugar synthase**	ThiS	thiosugar_synthase.hmm	3	300.0

Supplementary Table 10 Rules for sugar biosynthesis

Sugar	SMILES	Genes
**L-aculose**	CC1O[C@H](C=CC1=O)O	4,6-dehydratase<br>2,3-dehydratase<br>3,4-dehydratase<br>3-ketoreductase<br>4-ketoreductase<br>Epimerase<br>Oxidoreductase
**L-cinerulose A**	CC1O[C@H](CCC1=O)O	4,6-dehydratase<br>2,3-dehydratase<br>3,4-dehydratase<br>3-ketoreductase<br>4-ketoreductase<br>Epimerase
**L-rhodinose**	C[C@@H]1O[C@@H](CC[C@@H]1O)O	4,6-dehydratase<br>2,3-dehydratase<br>3,4-dehydratase<br>3-ketoreductase<br>4-ketoreductase<br>Epimerase
**Rednose**	CC1OC(C(N)=CC1=O)O	4,6-dehydratase<br>2,3-dehydratase<br>3,4-dehydratase<br>3-ketoreductase<br>4-ketoreductase<br>Epimerase
**L-cinerulose B**	CC1O[C@H]([C@@H](O)CC1=O)O	4,6-dehydratase<br>2,3-dehydratase<br>3,4-dehydratase<br>3-ketoreductase<br>4-ketoreductase<br>Epimerase
**O-methyl-L-amicetose**	COC1CC[C@@H](O[C@H]1C)O	4,6-dehydratase<br>2,3-dehydratase<br>3,4-dehydratase<br>3-ketoreductase<br>4-ketoreductase<br>Epimerase<br>O-methyltransferase
**4-O-methyl-L-rhodinose**	CO[C@H]1CC[C@@H](O[C@H]1C)O	4,6-dehydratase<br>2,3-dehydratase<br>3,4-dehydratase<br>3-ketoreductase<br>4-ketoreductase<br>Epimerase<br>O-methyltransferase

Sugar	SMILES	Genes
**L-daunosamine**	C[C@@H]1OC(C[C@@H]([C@@H]1O)N)O	4,6-dehydratase<br>2,3-dehydratase<br>4-ketoreductase<br>Epimerase<br>3-aminotransferase
**L-ristosamine**	CC1OC(CC(C1O)N)O	4,6-dehydratase<br>2,3-dehydratase<br>4-ketoreductase<br>Epimerase<br>4-aminotransferase
**D-digitoxose**	CC1OC(CC(C1O)O)O	4,6-dehydratase<br>2,3-dehydratase<br>3-ketoreductase<br>4-ketoreductase
**L-digitoxose**	CC1OC(CC(C1O)O)O	4,6-dehydratase<br>2,3-dehydratase<br>3-ketoreductase<br>4-ketoreductase<br>Epimerase
**2-deoxy-L-fucose**	CC1OC(CC(C1O)O)O	4,6-dehydratase<br>2,3-dehydratase<br>3-ketoreductase<br>4-ketoreductase<br>Epimerase
**D-olivose**	CC1O[C@@H](C[C@H]([C@@H]1O)O)O	4,6-dehydratase<br>2,3-dehydratase<br>3-ketoreductase<br>4-ketoreductase<br>Epimerase
**D-oliose**	CC1O[C@H](C[C@H]([C@H]1O)O)O	4,6-dehydratase<br>2,3-dehydratase<br>3-ketoreductase<br>4-ketoreductase
**4-oxo-L-vancosamine**	C[C@@H]1OC(C[C@](N)(C1=O)C)O	4,6-dehydratase<br>2,3-dehydratase<br>Epimerase<br>4-aminotransferase<br>C-methyltransferase
**D-forosamine**	CC1OC(CC[C@@H]1N(C)C)O	4,6-dehydratase<br>2,3-dehydratase<br>3,4-dehydratase<br>3-ketoreductase<br>4-aminotransferase<br>N,N-dimethyltransferase
**L-actinosamine**	COC1C(OC(CC1N)O)C	4,6-dehydratase<br>2,3-dehydratase

Sugar	SMILES	Genes
**L-vancosamine**	OC1O[C@H]([C@@H](O)[C@](C1)(N)C)C	4,6-dehydratase<br>2,3-dehydratase<br>4-ketoreductase<br>Epimerase<br>3-aminotransferase<br>C-methyltransferase
**L-vicenisamine**	CN[C@@H]1C(CC(OC1C)O)O	4,6-dehydratase<br>2,3-dehydratase<br>3-ketoreductase<br>4-aminotransferase<br>N-methyltransferase
**D-chalcose**	CO[C@H]1C[C@H](OC([C@@H]1O)O)C	4,6-dehydratase<br>3-ketoreductase<br>4-aminotransferase<br>O-methyltransferase<br>Oxidative deaminase
**D-mycarose**	CC1OC(C[C@](O)([C@H]1O)C)O	4,6-dehydratase<br>2,3-dehydratase<br>3-ketoreductase<br>4-ketoreductase<br>Epimerase<br>O-methyltransferase
**L-oleandrose**	CO[C@H]1C[C@H](O[C@H]([C@@H]1O)C)O	4,6-dehydratase<br>2,3-dehydratase<br>3-ketoreductase<br>4-ketoreductase<br>Epimerase<br>O-methyltransferase
**Olivomose**	COC1C(CC(OC1C)O)O	4,6-dehydratase<br>2,3-dehydratase<br>3-ketoreductase<br>4-ketoreductase<br>O-methyltransferase
**D-mycosamine**	C[C@H]1O[C@H]([C@H]([C@H]([C@@H]1O)N)O)O	4,6-dehydratase<br>3,4-ketoisomerase<br>3-aminotransferase<br>O-methyltransferase
**4-deoxy-4-thio-D-digitoxose**	CC1O[C@H](C[C@H](C1S)O)O	4,6-dehydratase<br>2,3-dehydratase<br>3-ketoreductase<br>Epimerase<br>Thiosugar synthase
**D-fucofuranose**	C[C@H]([C@H]1O[C@H]([C@H](C1O)O)O)O	4,6-dehydratase<br>4-ketoreductase
**D-fucose**	CC1OC([C@@H]([C@H]([C@H]1O)O)O)O	4,6-dehydratase<br>4-ketoreductase

Sugar	SMILES	Genes
**D-rhamnose**	C[C@@H]1O[C@@H]([C@@H]([C@@H]([C@H]1O)O)O)O	4,6-dehydratase<br>4-ketoreductase<br>Epimerase
**4-N-ethyl-4-amino-3-O-methoxy-2,4,5-trideoxypentose**	CCN[C@H]1CO[C@H](C[C@H]1OC)O	Dehydrogenase<br>Decarboxylase<br>2,3-dehydratase<br>3-ketoreductase<br>4-aminotransferase<br>N-ethyltransferase
**D-3-N-methyl-4-O-methyl-L-ristosamine**	CN[C@H]1CC(OC([C@@H]1OC)C)O	4,6-dehydratase<br>2,3-dehydratase<br>4-ketoreductase<br>Epimerase<br>4-aminotransferase<br>N-methyltransferase<br>O-methyltransferase
**N,N-dimethyl-L-pyrrolosamine**	CC1OC(CC(C1N(C)C)O)O	4,6-dehydratase<br>2,3-dehydratase<br>3-ketoreductase<br>Epimerase<br>4-aminotransferase<br>N,N-dimethyltransferase
**D-desosamine**	C[C@@H]1C[C@H](N(C)C)[C@@H](O)[C@H](O)O1	4,6-dehydratase<br>3,4-dehydratase<br>3-aminotransferase<br>N,N-dimethyltransferase<br>Oxidative deaminase
**L-megosamine**	C[C@@H]1OC(C[C@@H](N(C)C)[C@H]1O)O	4,6-dehydratase<br>2,3-dehydratase<br>4-ketoreductase<br>Epimerase<br>3-aminotransferase<br>N,N-dimethyltransferase
**Nogalamine**	OC1O[C@@H](C)[C@H](O)[C@@H](N(C)C)[C@@H]1O	4,6-dehydratase<br>2,3-dehydratase<br>4-ketoreductase<br>Epimerase<br>3-aminotransferase<br>N,N-dimethyltransferase
**L-rhodosamine**	CC1O[C@H](CC(N(C)C)[C@H]1O)O	4,6-dehydratase<br>2,3-dehydratase<br>4-ketoreductase<br>Epimerase<br>3-aminotransferase<br>N,N-dimethyltransferase

Sugar	SMILES	Genes
**D-angolosamine**	C[C@@H]1OC(CC(N(C)C)[C@H]1O)O	4,6-dehydratase<br>2,3-dehydratase<br>4-ketoreductase<br>3-aminotransferase<br>N,N-dimethyltransferase
**Kedarosamine**	OC1O[C@@H](C)[C@@H](N(C)C)[C@@H](O)C1	4,6-dehydratase<br>2,3-dehydratase<br>3-ketoreductase<br>4-aminotransferase<br>N,N-dimethyltransferase
**L-noviose**	CC1(C)[C@H](O)[C@@H](O)[C@@H](O)C(O)O1	4,6-dehydratase<br>4-ketoreductase<br>Epimerase<br>C-methyltransferase
**L-cladinose**	C[C@H]1[C@H](O)[C@](C)(OC)CC(O)O1	4,6-dehydratase<br>2,3-dehydratase<br>3-ketoreductase<br>4-ketoreductase<br>Epimerase<br>C-methyltransferase<br>O-methyltransferase
**2-N-methyl-D-fucosamine**	CN[C@H]1C(O[C@@H]([C@@H]([C@@H]1O)O)C)O	4,6-dehydratase<br>2,3-dehydratase<br>4-ketoreductase<br>N-methyltransferase
**D-digitalose**	CO[C@H]1[C@H]([C@H](O[C@H]([C@@H]1O)O)C)O	4,6-dehydratase<br>4-ketoreductase<br>O-methyltransferase
**2-O-methyl-rhamnose**	COC1[C@H]([C@H](OC([C@H]1O)O)C)O	4,6-dehydratase<br>4-ketoreductase<br>Epimerase<br>O-methyltransferase
**3-O-methyl-rhamnose**	COC1[C@@H](OC([C@@H](C1O)O)C)O	4,6-dehydratase<br>4-ketoreductase<br>Epimerase<br>O-methyltransferase
**6-deoxy-3-C-methyl-L-mannose**	C[C@@H]1OC([C@@H]([C@](O)([C@H]1O)C)O)O	4,6-dehydratase<br>4-ketoreductase<br>Epimerase<br>C-methyltransferase
**4,6-dideoxy-4-hydroxylamino-D-glucose**	CC1OC(C(C(C1NO)O)O)O	4,6-dehydratase<br>4-aminotransferase

Sugar	SMILES	Genes
**3-N,N-dimethyl-L-eremosamine**	OC1C[C@](C)(N(C)C)[C@@H](O)[C@H](C)O1	4,6-dehydratase<br>2,3-dehydratase<br>4-ketoreductase<br>Epimerase<br>C-methyltransferase<br>N,N-dimethyltransferase
**Chromose**	CC1OC(CC(C1OC(C)=O)O)O	4,6-dehydratase<br>2,3-dehydratase<br>3-ketoreductase<br>4-ketoreductase<br>Acetyltransferase
**4-O-carbamoyl-D-olivose**	CC1OC(CC(C1OC(N)=O)O)O	4,6-dehydratase<br>2,3-dehydratase<br>3-ketoreductase<br>4-ketoreductase<br>Carbamoyltransferase
**D-ravidosamine**	C[C@H]1O[C@@H]([C@@H]([C@@H](N(C)C)[C@H]1O)O)O	4,6-dehydratase<br>3,4-ketoisomerase<br>3-aminotransferase<br>N,N-dimethyltransferase
**3-N,N-dimethyl-D-mycosamine**	C[C@H]1O[C@H]([C@@H]([C@@H](N(C)C)[C@@H]1O)O)O	4,6-dehydratase<br>3,4-ketoisomerase<br>3-aminotransferase<br>N,N-dimethyltransferase
**2,3-O-dimethyl-L-rhamnose**	COC1[C@@H](OC([C@@H](C1OC)O)C)O	4,6-dehydratase<br>4-ketoreductase<br>Epimerase<br>O-methyltransferase
**2,4-O-dimethyl-L-rhamnose**	CO[C@H]1C(O[C@H](C(C1O)OC)O)C	4,6-dehydratase<br>4-ketoreductase<br>Epimerase<br>O-methyltransferase
**3,4-O-dimethyl-L-rhamnose**	CO[C@H]1C(O[C@H](C(C1OC)O)O)C	4,6-dehydratase<br>4-ketoreductase<br>Epimerase<br>O-methyltransferase
**2-thioglucose**	OC1[C@H](S)[C@@H](O)[C@H](O)[C@@H](CO)O1	Thiosugar synthase
**Olivomycose**	C[C@@H]1O[C@H](C[C@](O)([C@H]1OC(C)=O)C)O	4,6-dehydratase<br>2,3-dehydratase<br>3-ketoreductase<br>4-ketoreductase<br>Epimerase<br>C-methyltransferase<br>Acetyltransferase

Sugar	SMILES	Genes
**4-N,N-dimethylamino-4-deoxy-5-C-methyl-l-rhamnose**	CN(C1C(C(C(OC1(C)C)O)O)O)C	4,6-dehydratase<br>Epimerase<br>4-aminotransferase<br>C-methyltransferase<br>N,N-dimethyltransferase<br>Acetyltransferase
**2,3,4-tri-O-methylrhamnose**	CO[C@@H]1[C@H](O[C@@H]([C@H]([C@H]1OC)OC)O)C	4,6-dehydratase<br>4-ketoreductase<br>Epimerase<br>O-methyltransferase
**4-O-acetyl-L-arcanose**	OC1C[C@@](C)(OC)[C@H](OC(C)=O)[C@H](C)O1	4,6-dehydratase<br>2,3-dehydratase<br>3-ketoreductase<br>4-ketoreductase<br>Epimerase<br>C-methyltransferase<br>Acetyltransferase
**3-N-acetyl-D-ravidosamine**	C[C@H]1O[C@@H]([C@@H]([C@@H](N(C(C)=O)C)[C@H]1OC(C)=O)O)O	4,6-dehydratase<br>3,4-ketoisomerase<br>3-aminotransferase<br>N,N-dimethyltransferase<br>Acetyltransferase
**3-O-carbamoyl-L-noviose**	CC1([C@@H]([C@H]([C@H](C(O1)O)O)OC(N)=O)O)C	4,6-dehydratase<br>2,3-dehydratase<br>4-ketoreductase<br>C-methyltransferase<br>Carbamoyltransferase
**L-nogalose**	COC1C(OC(C(C1(OC)C)OC)O)C	4,6-dehydratase<br>4-ketoreductase<br>Epimerase<br>C-methyltransferase<br>O-methyltransferase
**4-O-acetyl-D-ravidosamine**	C[C@H]1O[C@@H]([C@@H]([C@@H](N(C)C)[C@H]1OC(C)=O)O)O	4,6-dehydratase<br>3,4-ketoisomerase<br>3-aminotransferase<br>N,N-dimethyltransferase<br>Acetyltransferase
**3-O-carbamoyl-4-O-methyl-L-noviose**	CC1(C)[C@H](OC)[C@@H](OC(N)=O)[C@@H](O)C(O)O1	4,6-dehydratase<br>2,3-dehydratase<br>4-ketoreductase<br>C-methyltransferase<br>O-methyltransferase<br>Carbamoyltransferase
**3-N-acetyl-4-O-acetyl-D-ravidosamine**	C[C@H]1O[C@@H]([C@@H]([C@@H](N(C(C)=O)C)[C@H]1OC(C)=O)O)O	4,6-dehydratase<br>3,4-ketoisomerase<br>3-aminotransferase<br>N,N-dimethyltransferase<br>Acetyltransferase

Sugar	SMILES	Genes
**3-(5'-methyl-2'-pyrrolylcarbonyl-)4-O-methyl-L-noviose**	CO[C@@H]1[C@@H](C([C@@H](OC1(C)C)O)O)OC(C2=CC=C(N2)C)=O	4,6-dehydratase<br>4-ketoreductase<br>Epimerase<br>C-methyltransferase<br>O-methyltransferase<br>Pyrrolyltransferase
**Madurose**	O[C@@H]1[C@H](O)[C@@](O)(C)[C@H](N)CO1	Dehydrogenase<br>Decarboxylase<br>C-methyltransferase<br>4-aminotransferase
**4-N-methyl-4-amino-3-O-methoxy-2,4,5-trideoxypentose**	O[C@@H]1C[C@H](OC)[C@@H](NC)CO1	Dehydrogenase<br>Decarboxylase<br>2,3-dehydratase<br>3-ketoreductase<br>4-aminotransferase<br>N-methyltransferase

Supplementary Table 11 Potensimicin gene cluster analysis

Locus	Predicted Function	Strand	Amino Acids
D459DRAFT_04885	Glycosyltransferase	+	401
D459DRAFT_04886	Hypothetical protein	+	99
D459DRAFT_04887	Hypothetical protein	+	281
D459DRAFT_04888	Hypothetical protein	+	244
D459DRAFT_04889	NDP-hexose-2,3-dehydratase	+	449
D459DRAFT_04890	dTDP-4-amino-4,6-transaminase	+	374
D459DRAFT_04891	N,N-dimethyltransferase	+	247
D459DRAFT_04892	dTDP-4-dehydrorhamnose 3,5-epimerase	+	228
D459DRAFT_04893	4-ketoreductase	+	341
D459DRAFT_04894	Hypothetical protein	+	271
D459DRAFT_04895	AAA ATPase domain	-	989
D459DRAFT_04896	Polyketide synthase	+	4564
D459DRAFT_04897	Polyketide synthase	+	3658
D459DRAFT_05970	Polyketide synthase	+	1627
D459DRAFT_05969	Polyketide synthase	+	1353
D459DRAFT_04117	Beta-glucosidase-related glycosidase	+	485
D459DRAFT_04116	Cytochrome P450	+	399
D459DRAFT_04115	Glycosyltransferase	+	424
D459DRAFT_04114	N,N-dimethyltransferase	+	239
D459DRAFT_04113	Beta-glucosidase-related glycosidase	+	793
D459DRAFT_04112	MFS / Sugar transport protein	+	418
D459DRAFT_04111	Phosphohydrolase	-	281
D459DRAFT_04110	Glucose-1-phosphate thymidylyltransferase	+	285
D459DRAFT_04109	dTDP-4-amino-4,6-dehydratase	+	341
D459DRAFT_04108	3,5-ketoisomerase	+	150
D459DRAFT_04107	dTDP-4-amino-4,6-transaminase	+	389
D459DRAFT_04106	Adenine-N(6)-methyltransferase	+	293

Supplementary Table 12 NMR spectroscopic data for potensimicin (7) (700 MHz, in DMSO-d_6)[a].

Position	δ_H (mult.)	δ_C	Position	δ_H (mult.)	δ_C
1	0.80 (*t*, 7.30)	9.36	14	3.78 (*m*)	79.42
2a	1.54 (*m*)	22.50	15	2.83 (*quint.*, 7.19, 7.37)	48.72
2b	1.54 (*m*)		16	1.22 (*d*, 7.32)	12.7
3	4.83 (*m*)	76.5	17	-	208.17
4	2.63 (*m*)	37.1	18	3.96 (*quart.*, 6.74, 6.89)	49.1
5	0.98 (*d*, 6.02)	10.03	19	1.15 (*d*, 6.84)	13.84
6	6.64 (*m*)	126.1	20	-	169.25
7	6.14 (*d*, 14.2)	146.8	1'	4.17 (*d*, 7.06)	102.8
8	-	201.95	2'	3.23 (*t*, 8.91)	68.17
9	2.51 (*ov*)	41.93	3'	2.44 (*ov*)	70.0
10	1.00 (*d*, 6.81)	15.07	4'	2.49 (*ov*)	40.73
11a	1.49 (*m*)	34.37	5'	2.49 (*ov*)	40.73
11b	1.08 (*m*)		6'	3.05 (*t*, 9.31)	69.1
12	-	-	7'	3.19 (*quint.*, 5.64, 6.84)	71.5
13	0.87 (*d*, 6.58)	16.00	8'	1.12 (*d*, 5.94)	16.96

[a]Chemical shift δ and (multiplicity, J in Hz).

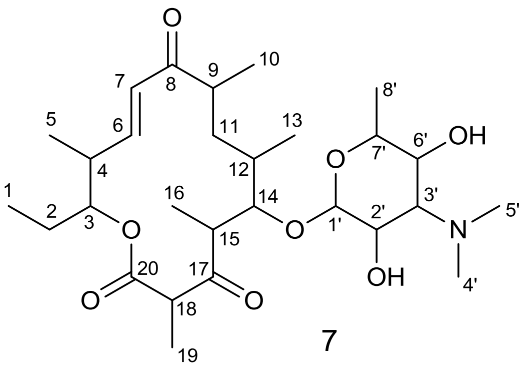

7

Supplementary Table 13 NMR spectroscopic data for potensimicin (7) (700 MHz, in $CDCl_3$)[a].

Position	δ_H (mult.)	δ_C	Position	δ_H (mult.)	δ_C
1	0.79 (*m*)	9.95	14	3.98 (*m*)	78.56
2a	1.43 (*ov*)	22.48	15	2.78 (*m*)	48.57
2b	1.48 (*ov*)		16	1.21 (*ov*)	13.1
3	4.79 (*m*)	77.9	17	-	207.86
4	2.6 (*ov*)	37.77	18	3.75 (*m*)	49.93
5	0.98 (*m*)	11.58	19	1.21 (*ov*)	13.92
6	6.56 (*d*, 13.45)	146.83	20	-	168.82
7	5.99 (*d*, 14.7)	126.84	1'	4.22 (*m*)	103.38
8	-	202.59	2'	3.35 (*m*)	69.04
9	2.59 (*ov*)	41.61	3'	2.53 (*ov*)	70.93
10	0.97 (*m*)	15.12	4'	2.56 (*ov*)	41.21
11a	1.38 (*ov*)	35.94	5'	2.56 (*ov*)	41.21
11b	1.07 (*m*)		6'	3.11 (*m*)	69.66
12	1.5 (*ov*)	35.64	7'	3.22 (*m*)	72.5
13	0.87 (*m*)	16.51	8'	1.16 (*m*)	17.22

[a]Chemical shift δ and (multiplicity, J in Hz).

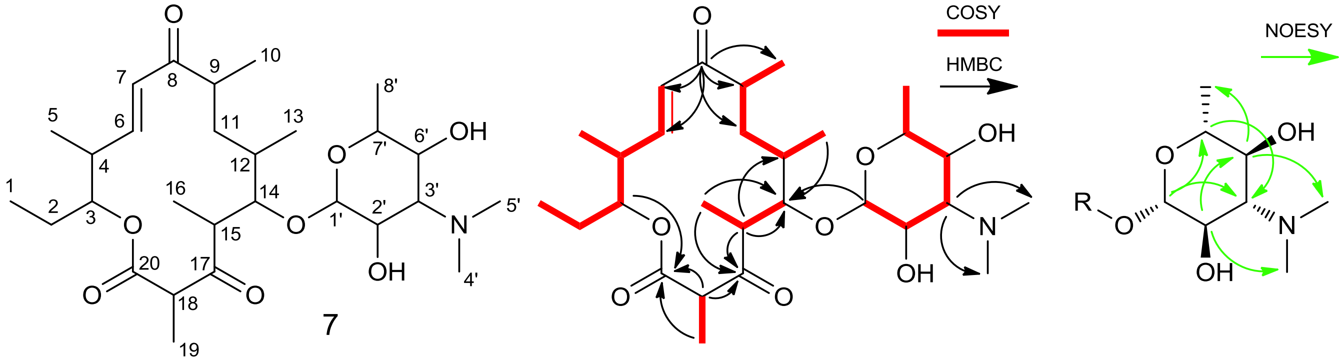

7

Supplementary Table 14 Minimum inhibitory concentrations (MICs) of potensimicin and thanamycin.

	B. subtilis 168	*S. aureus* Newman
Potensimicin	2 μg/mL	8 μg/mL
Erythromycin	0.125 μg/mL	0.5 μg/mL

	S. cerevisiae
Thanamycin	0.0625 μg/mL
Syringomycin E	2 μg/mL

Supplementary Table 15 Detection of natural product standards at low concentrations.

Natural Product	Class	Concentration (μg/mL)	Detected Scans	Avg. P1	Avg. P2
Daptomycin	Lipodepsipeptide	1	3	37.0	26.5
Erythromycin	Glycosylated polyketide	1	6	23.3	17.4
Thiostrepton	Ribosomal thiopeptide	1	3	7.1	12.6
Lincomycin	Lincosamide	1	7	8.8	18.3
Novobiocin	Coumarin	1	4	57.9	23.2
Vancomycin	Glycopeptide	1	7	45.8	49.0
Capreomycin	Cyclic peptide	10	5	7.3	9.5
Nystatin	Glycosylated polyketide	1	4	35	9.7
Bacitracin	Branched cyclic peptide	1	2	22.8	34.2
Gramicidin A	Nonribosomal peptide	1	8	40.9	40.4
Polymyxin B	Branched cyclic peptide	1	3	24.5	30.3
Valinomycin	Cyclic depsipeptide	1	7	24.8	16.0

Supplementary Table 16 Thanamycin gene cluster analysis

Locus	Predicted Function	Strand	Amino Acids
ThaE	Cyclic peptide ABC transporter	-	566
ThaF	SyrP hydroxylase	-	353
ThaC1	NRPS	+	615
ThaC2	Chlorinating enzyme	+	312
ThaD	Thioesterase	+	415
ThaG	Branched chain amino acid permease	+	233
ThaH	Branched chain amino acid permease	+	104
ThaI	AraC family transcription factor	+	172
ThaJ	putative beta hydroxylase	-	204
ThaK	MFS transporter	-	457
ThaL	putative transcription factor	-	335
ThaA	NRPS	+	5380
ThaB	NRPS	+	4236

Supplementary Table 17 NMR spectroscopic data for thanamycin (8) (700 MHz, in DMSO-d_6)[a].

Position	δ_H mult.	δ_C	Position	δ_H mult.	δ_C
1	0.84 (*t*, 6.1)	14	34a	3.36 (*m*)	57.46
2	1.24 (*ov*)	22.17	34b	3.3 (*m*)	
3	1.21 (*ov*)	31.38	35a	2.03 (*m*)	33.33
4-10	1.23 (*ov*)	29.17	35b	1.84 (*ov*)	
11a	1.4 (*ov*)	25.42	36	4.09 (*m*)	51.92
11b	1.2 (*ov*)		37	-	172.78
12a	1.48 (*m*, 11.7)	32.87	38	8.05 (*ov*)	-
12b	1.2 (*ov*)		39	8.97 (*s*)	133.82
13	3.18 (*m*)	73.57	40	7.24 (*s*)	116.9
14	3.56 (*m*)	71.63	41	-	129.4
15a	2.36 (*d*, 13.8)	39.15	42a	3.23 (*m*)	26.96
15b	2.14 (*dd*, 10.6)		42b	2.98 (*m*)	
16	-	171.81	43	4.71 (*ov*)	51.01
17	7.7 (*ov*)	-	44	-	171.85
18a	4.38 (*m*)	63.89	45	8.07 (*ov*)	-
18b	4.16 (*ov*)		46	1.19 (*ov*)	20.61
19	4.66 (*m*)	50.68	47	3.84 (*m*)	66.59
20	-	168.64	48	4.18 (*ov*)	60.17
21	9.04 (*s*)	-	49	-	170.42
22	7.85 (*ov*)	-	50	9.65 (*s*)	-
23a	2.76 (*m*)	38.85	51	1.67 (*d*, 5.6)	13.07
23b	2.76 (*m*)		52	6.47 (*m*)	129.78
24a	1.63 (*ov*)	20.98	53	-	131.7
24b	1.44 (*ov*)		54	-	163.69
25a	1.8 (*m*)	35.33	55	7.62 (*ov*)	-
25b	1.74 (*m*)		56	-	169.47
26	-	81.31	57	4.69 (*ov*)	71.38
27	-	171.8	58	4.94 (*m*)	55.61
28	8.21 (*ov*)	-	59	-	169.33
29	-	173.03	60	7.94 (*ov*)	-
30a	2.71 (*ov*)	36.43	61a	3.51 (*m*)	45.61
30b	2.48 (*ov*)		61b	3.41 (*m*)	
31	4.56 (*m*)	49.67	62	4.2 (*ov*)	71.2
32	-	170.36	63	4.78 (*d*, 7.5)	54.3
33	7.89 (*ov*)	-	64	-	170.54

[a]Chemical shift δ and (multiplicity, *J* in Hz).

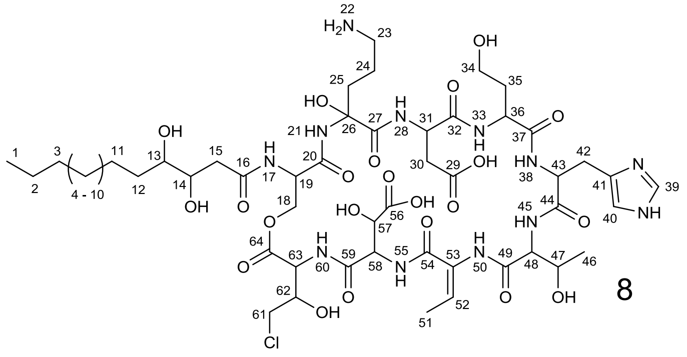

8

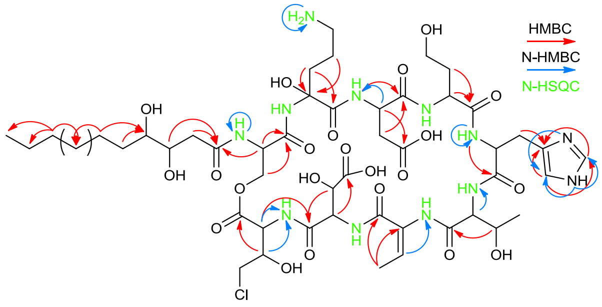

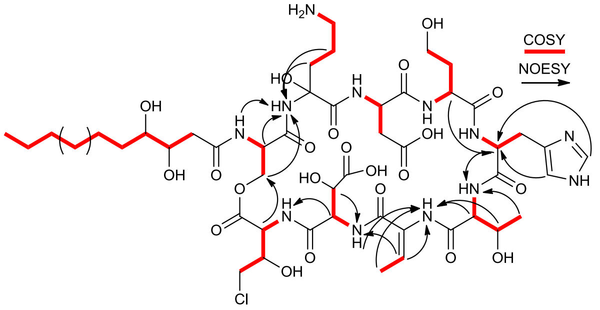

Supplementary Note 1 Structure elucidation

High Resolution Mass Spectra

A stock solution of 20 mg/mL of each compound was diluted to a final concentration of 10 μg/mL in water with 0.1% formic acid. This solution was directly infused at a rate of ~3 μL per min into a Thermo Finnigan LTQ OrbiTrap XL mass spectrometer running Xcaliber 2.07 and TunePlus 2.4 SP1. High resolution MS was acquired using an electrospray ionization source and fragmentation was obtained through collision induced dissociation (CID). The instrument was operated in the positive mode using a maximum resolution of 100, 000. Data was acquired for approximately 1 min.

Mass Spectra Fragmentation

Mass spectral fragmentation patterns are shown for acidobactins, vacidobactins, and variobactin below.

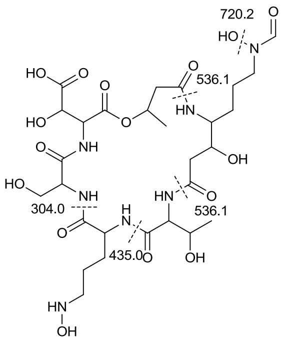

Acidobactin A (2)

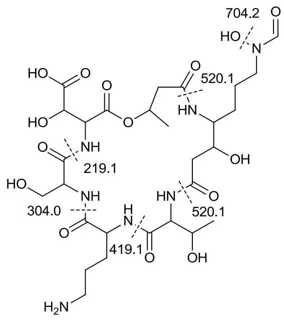

Acidobactin B (3)

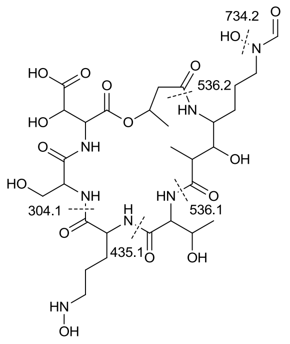

Vacidobactin A (4)

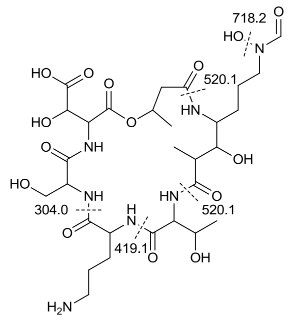

Vacidobactin B (5)

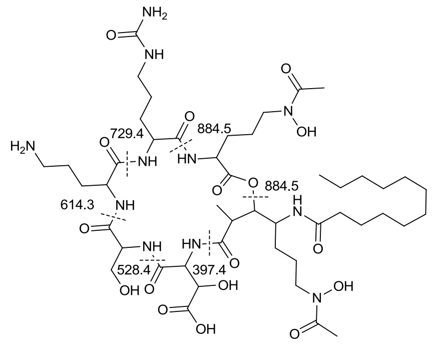

Variobactin A (6)

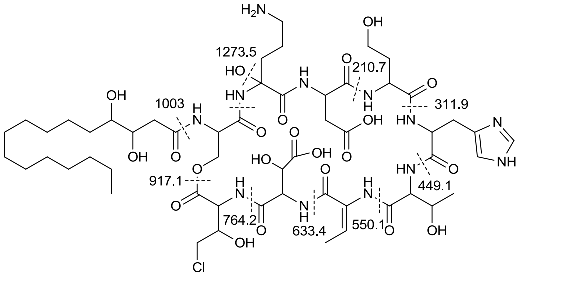

Thanamycin (8)

NMR Methods and Structural Characterization

NMR spectra were measured on a Bruker Avance 700 spectrometer equipped with a 5 mm inverse detection probe and using TMS as an internal standard. Lyophilized samples were dissolved in D_2O,

$CDCl_3$, or d_6-DMSO (*Sigma Aldrich*) and spectra were recorded at 297 K. NMR experiments were processed and analyzed with Bruker TOPSPIN 2.1 or MestReNova 9.0. Chemical shifts (δ) expressed in parts per million (ppm) and coupling constants (J) are reported in Hertz (Hz). Assembly of individual amino acids to form the final linear structure was accomplished by considering long-range ^{1}H-^{1}H NOESY and ROESY correlations, and ^{1}H-^{13}C HMBC correlations from protons adjacent carbonyl carbons, as well as by assignments of 2D ^{1}H-^{1}H COSY and 2D ^{1}H-^{13}C HSQC correlations.

Supplementary note 2 – The sugar prediction algorithm is represented by the following pseudocode:

```
n = number of glycosyltransferases in cluster
for glycosyltransferase in cluster glycosyltransferases
     if glycosyltransferase substrate == hexose
          n--
minimum remaining in pathway = 999;
minimum remaining in cluster = 999;

sugar combinations = get all combinations of sugars of size n

for sugar combination in sugar combinations
     remainingInCluster = number of cluster sugar genes
     remainingInPathway = number of pathway sugar genes

     for pathway sugar gene in pathway sugar genes
          if cluster genes contain pathway sugar gene
               remainingInPathway--

     for cluster sugar gene in cluster sugar genes
          if pathway genes contain cluster sugar genes
               remainingInPathway--
```

```
    if remaining in pathway <= minimum remaining in pathway
            and remaining in cluster <= minimum remaining in cluster
        if remaining in pathway < minimum remaining in pathway
                or remaining in cluster < minimum remaining in cluster
            clear combinatorial sugar set
        minimum remaining in pathway = remaining in pathway
        minimum remaining in cluster = remaining in cluster
        add sugar combination to combinatorial sugar set

return combinatorial sugar set
```
